# Discovery of Substituted Di(pyridin-2-yl)-1,2,4-thiadiazol-5-amines as Novel Macrofilaricidal Compounds for the Treatment of Human Filarial Infections

**DOI:** 10.1021/acs.jmedchem.2c00960

**Published:** 2022-08-16

**Authors:** Natalie Hawryluk, Dale Robinson, Yixing Shen, Graham Kyne, Matthew Bedore, Sanjay Menon, Stacie Canan, Thomas von Geldern, Simon Townson, Suzanne Gokool, Alexandra Ehrens, Marianne Koschel, Nathaly Lhermitte-Vallarino, Coralie Martin, Achim Hoerauf, Geraldine Hernandez, Deepak Dalvie, Sabine Specht, Marc Peter Hübner, Ivan Scandale

**Affiliations:** † Bristol Myers Squibb, San Diego, California 92121, United States; ‡ Zoetis, Kalamazoo, Michigan 49001, United States; § Embedded Consulting, Richmond, Illinois 60071, United States; ∥ Northwick Park Institute for Medical Research, London HA1 3UJ, UK; ⊥ Institute for Medical Microbiology, Immunology & Parasitology, University Hospital Bonn, 53127 Bonn, Germany; # German Center for Infection Research (DZIF), partner site Bonn-Cologne, 53127 Bonn, Germany; ∇ Unité Molécules de Communication et Adaptation des Microorganismes (MCAM, UMR 7245), Muséum national d’Histoire Naturelle, Paris 75005, France; ○ Drugs for Neglected Diseases Initiative, Geneva 1204, Switzerland

## Abstract

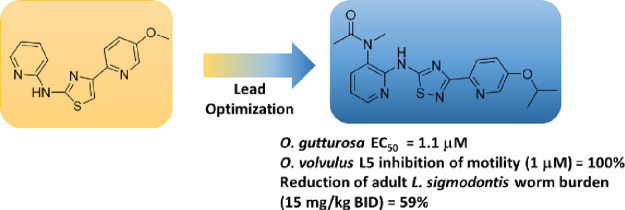

Filarial diseases, including lymphatic filariasis and onchocerciasis, are considered among the most devastating of all tropical diseases, affecting about 145 million people worldwide. Efforts to control and eliminate onchocerciasis are impeded by a lack of effective treatments that target the adult filarial stage. Herein, we describe the discovery of a series of substituted di(pyridin-2-yl)-1,2,4-thiadiazol-5-amines as novel macrofilaricides for the treatment of human filarial infections.

## Introduction

Onchocerciasis and lymphatic filariasis (LF) affect an estimated 145 million people worldwide, creating a serious health burden in endemic areas such as sub-Saharan Africa and India. Filarial nematodes are the pathogens responsible for a number of parasitic diseases; *Onchocerca volvulus* causes onchocerciasis (river blindness), and *Wuchereria bancrofti*, *Brugia malayi*, and *Brugia timori* cause LF (elephantiasis).^1^ Current therapies target the juvenile worms (microfilariae), allowing adult worms to continue producing microfilariae. These treatments only temporarily stop transmission, thereby requiring repeat treatments for up to 15 years, depending on the disease.^2^ The need for repeated drug administration, concerns about serious *Loa loa*-related adverse events,^3^ and the possible emergence of ivermectin-resistant *O. volvulus* have heightened the need for compounds that exhibit adult stage selectivity (macrofilaricidal) or long-lasting sterilizing effects.^4^

Current efforts to control and eliminate onchocerciasis are hindered by the lack of medicines that target the adult worm stage.^2^ In addition, a major challenge hampering drug discovery is finding suitable preclinical animal models as *O. volvulus* can only develop fully in humans and primates. While assays for testing against *O. volvulus* have been established, they have limitations. The use of surrogate parasites is required for both *ex vivo* and *in vivo* evaluation due to limited accessibility of the adult stage of *O. volvulus*.^5^ Widely used surrogate parasites include the bovine filariae *Onchocerca gutturosa* and *Litomosoides sigmodontis* as the most suitable sources of viable adult filariae for high-throughput screening.^6^*L. sigmodontis*, a natural filarial parasite of rodents, has been used as a standard model to validate macrofilaricidal drug candidates in a consortium led by the Bill & Melinda Gates Foundation, Drugs for Neglected Diseases initiative (DNDi), academia, and pharmaceutical companies and has supported the advancement of a few macrofilaricidal candidates through preclinical and clinical development.^7−14^

**Figure 1:**
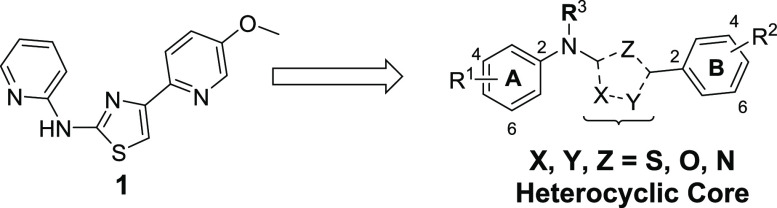
Initial amino thiazole hit molecule, **1**, and lead optimization progression to a novel scaffold.

We recently reported a platform utilizing surrogate nematodes in phenotypic *ex vivo* assays to assess activity across various parasites for the identification of potential novel macrofilaricidal compounds for further drug discovery lead optimization efforts. These efforts led to a series of amino-thiazole molecules demonstrating *ex vivo* killing of adult *O. gutturosa*, *B. malayi*, *B. pahangi*, and *L. sigmodontis*.^15^ Compound **1** (Figure 1) showed a significant 68% reduction of adult *L. sigmodontis* worms in infected mice, thus establishing proof of principle for this series and suggesting the potential for further optimization. Herein, we describe the discovery of a series of substituted di(pyridin-2-yl)-1,2,4-thiadiazol-5-amines as novel macrofilaricides for the treatment of onchocerciasis.

As part of our lead optimization efforts, our initial focus was replacement of the thiazole ring core to address potential metabolic instability promiscuity and/or metabolism to a reactive species.^16−18^

Compound **1** showed sufficient plasma exposure in male CD-1 mice when dosed orally (30 mg/kg); however, dosing was required three times per day (TID) to achieve plasma drug concentrations that would remain continuously above EC_50_ for 24 h. Although multiple compounds in the amino thiazole (AT) series had reasonable S9 stability, plasma exposure could not be maintained for 24 h. This could be explained by reported metabolism of thiazole moieties.^18,19^

## Chemistry

**Scheme 1:**
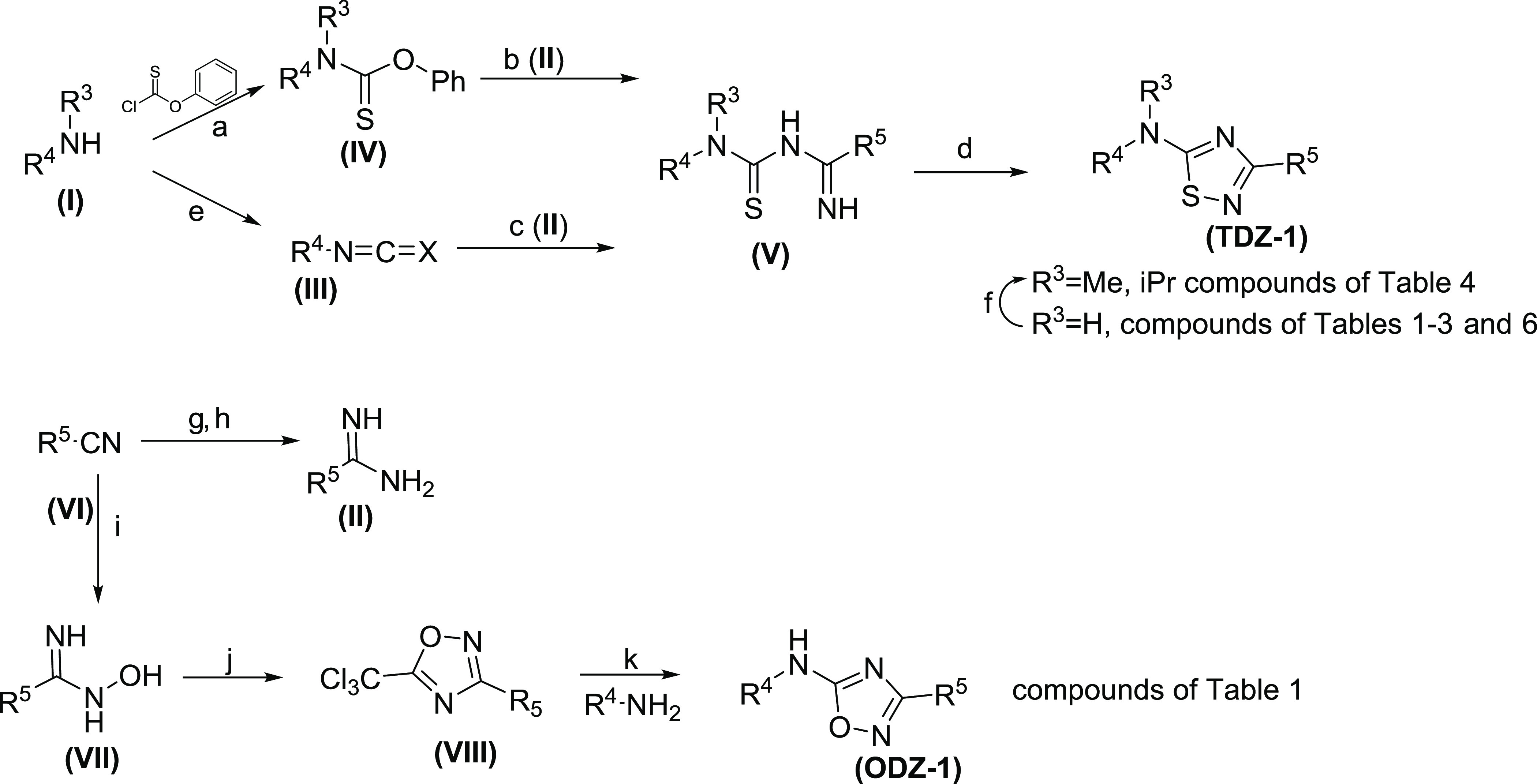
Synthesis of Di(pyridin-2-yl)-1,2,4-thia-diazol-5-amine and Di(pyridin-2-yl)-1,2,4-oxa-diazol-5-amine Reagents and conditions: (a) K_2_CO_3_, THF, 0–25 °C, 16 h, 70–92%, (b) (**II**), DMSO, KOtBu, 25 °C, 16 h 12–72%, (c) Et_3_N, DCM/acetone, 15 °C, 2 h, 17–49%, (d) I_2_, H_2_O_2_, EtOH, 25 °C, 1 h, 14–70%, (e) thiophosgene, DCM, 0 °C, 1 h, 66%, (f) MeI or *i*Pr-I, *n*BuLi, THF, −70 °C, 1–16 h, 31–75% (g) NaOMe, MeOH, 15 °C, 14 h, (h) NH_4_Cl, 70 °C, 2 h (39–93% for panels (g, h)), (i) NH_2_OH HCl, Et_3_NH_2_, EtOH, 80 °C, 16 h, (j) (Cl_3_CO)_2_O, toluene, 110 °C, 16 h. (65–97% for panels (i, j)), (k) NaH, THF, 25 °C, 30 min, 12–25%.

Compounds containing the 1,2,4-thiadiazole core (**TDZ-1**) are synthesized via a convergent synthesis outlined in Scheme 1. Compounds in Tables 1–3 and 6 are formed by cyclization of (**V**), which is formed by the addition of an amidine (**II**) with an isothiocyanate (**III**). Isothiocyanate (**III**) can be obtained by reaction of commercially available amines with thiophosgene. Amidine (**II**) is formed by a Pinner reaction of an appropriately substituted nitrile (**VI**). Compounds in Table 4 are formed by either direct alkylation of (**TDZ-1**) or by addition of carbamothioate (**IV**) to amidine (**II**) to form (**V**). Compounds containing the 1,2,4-oxadiazole core (**ODZ-1**) are synthesized, as described in Scheme 1. Compounds in Table 1 are prepared by the cyclocondensation of amidine oxime (**VII**) and trichloroacetic anhydride. Amidine oxime (**VII**) is formed from the corresponding nitrile (**VI**), and the introduction of the B-ring is achieved via SN_Ar_ chemistry with the analogous amine.

**Table 1:**
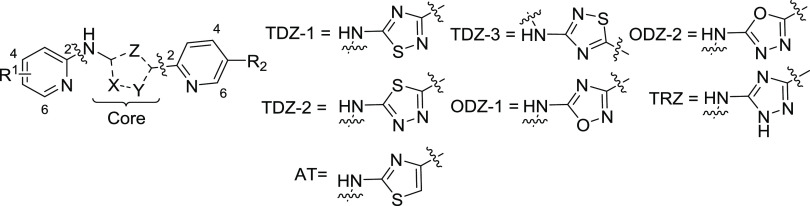

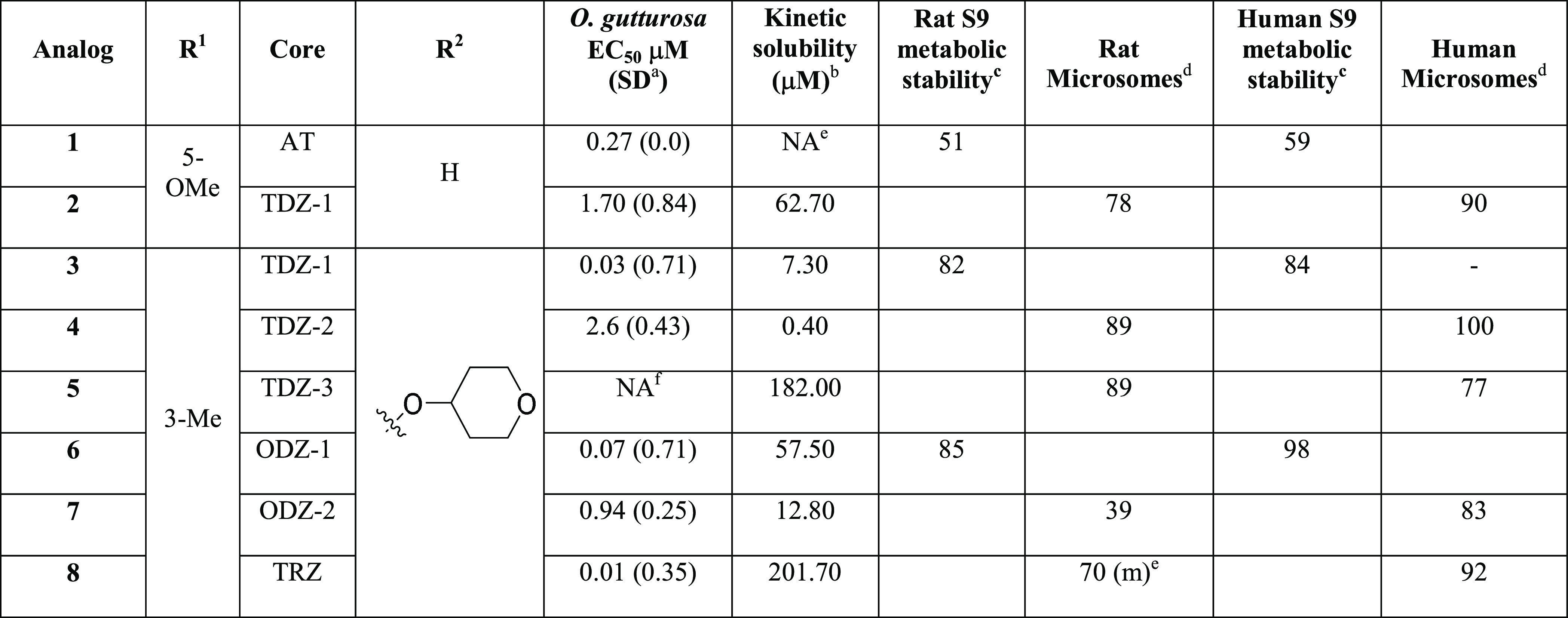
*Ex VivoO. gutturosa* Activity across Heterocyclic Core Variants

a Standard deviation.

b The standard kinetic solubility assay runs on 200 mM concentration range using 10 mM DMSO stock solutions.

c Liver S9 metabolic stability assay at 3 μM, % remaining @ 60 min.

d Liver microsome metabolic stability assay at 3 μM, % remaining measured @ 30 min.

e Tested in the mouse not rat.

f NA = not available due to COVID; however, **5** demonstrated 25% reduction of *L. sigmodontis* adult worm motility at 100 nM.

**Table 2:**
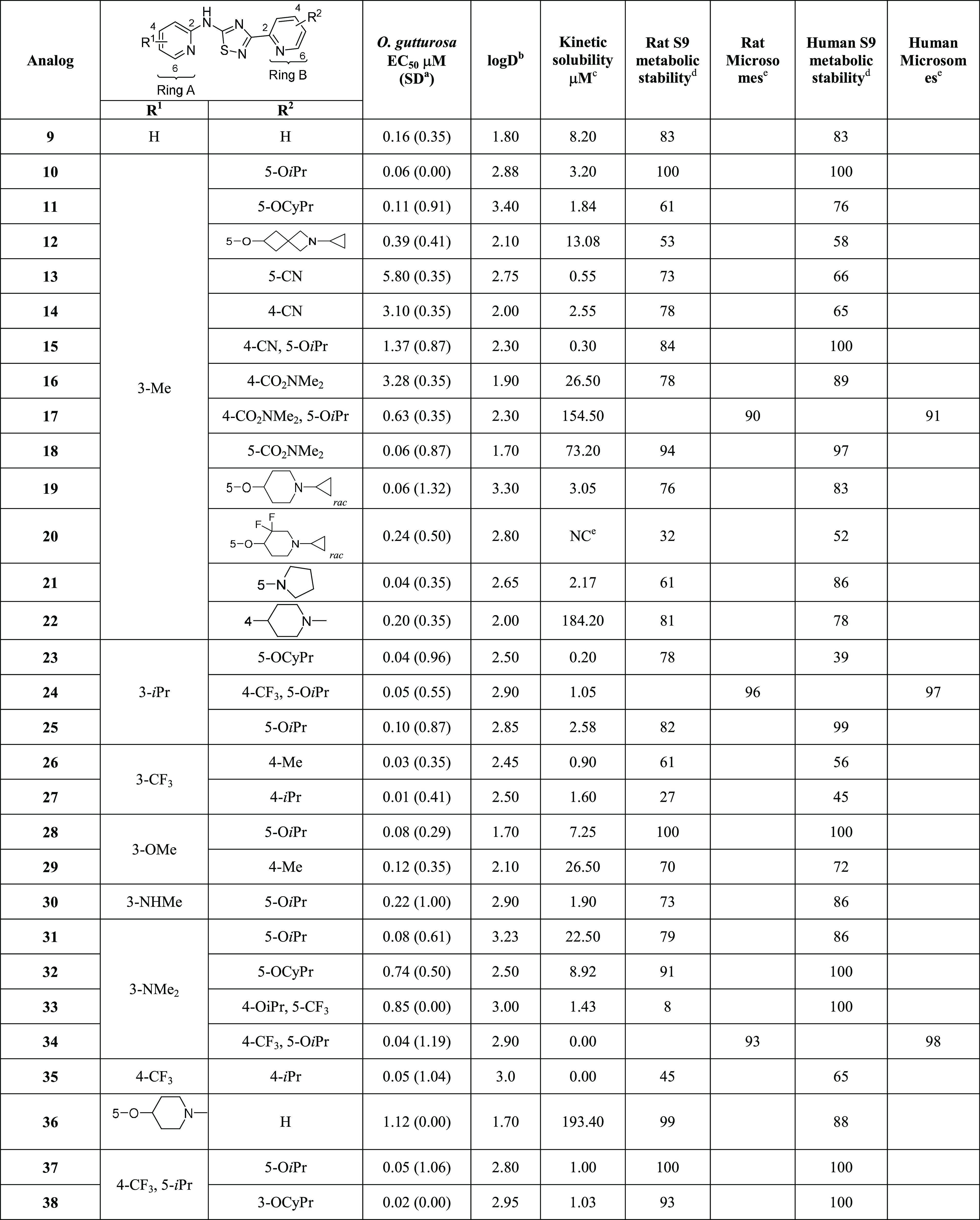
*Ex VivoO. gutturosa* Activity

a Standard deviation.

b The log *D* assay runs on 1 M concentration range using 10 mM DMSO stock solutions measured at pH 7.4.

c The standard kinetic solubility assay runs on 200 mM concentration range using 10 mM DMSO stock solutions.

d The liver S9 metabolic stability assay at 3 μM, % remaining @ 60 min.

e Tested in the mouse and human liver microsome metabolic stability assay at 3 μM, % remaining measured @ 30 min.

f NC = not calculated.

**Table 3:**
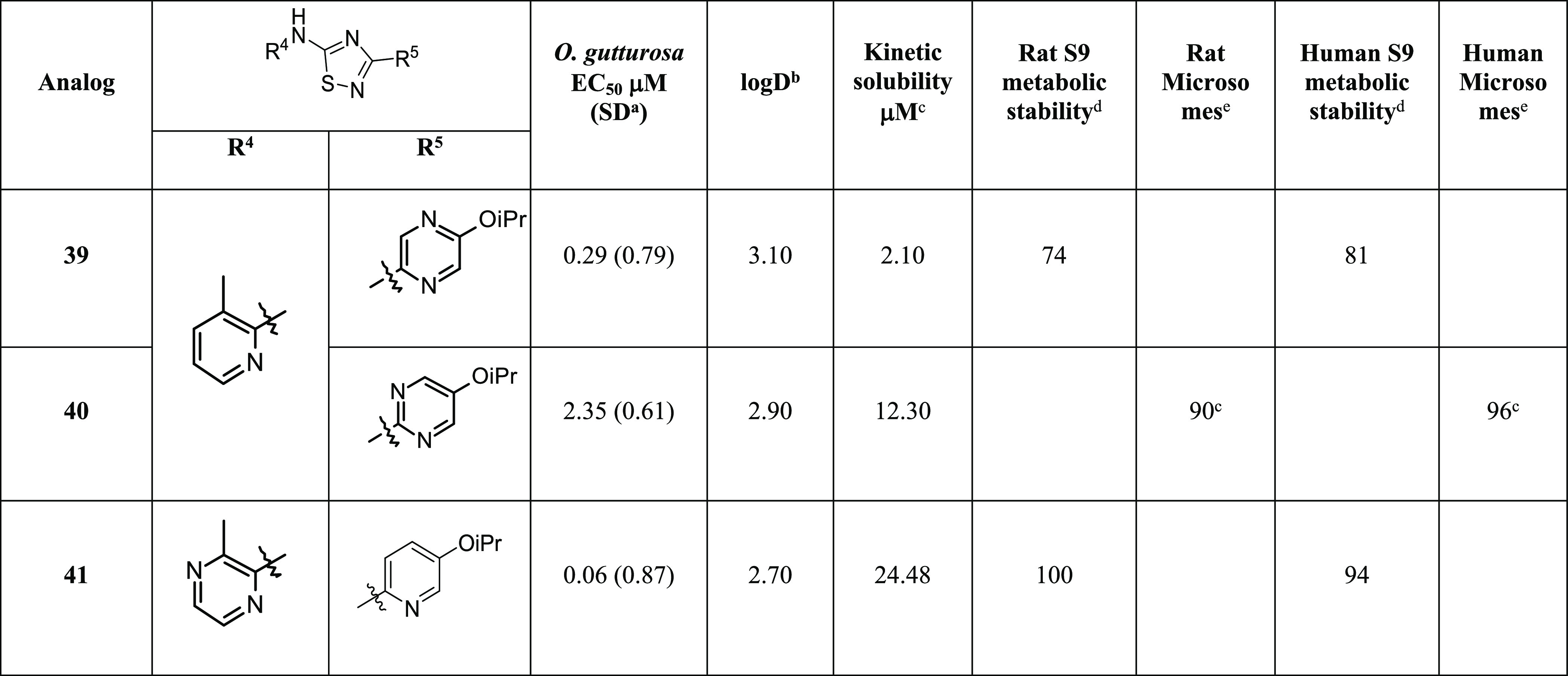
*Ex VivoO. gutturosa* Activity with A- and B-Ring Heterocyclic Replacements

a Standard deviation.

b The log *D* assay runs on 1 M concentration range using 10 mM DMSO stock solutions measured at pH 7.4.

c The standard kinetic solubility assay runs on 200 mM concentration range using 10 mM DMSO stock solutions.

d Liver S9 metabolic stability assay at 3 μM, % remaining @ 60 min.

e Tested in the mouse and human liver microsome metabolic stability assay at 3 μM, % remaining measured @ 30 min.

**Table 4:**
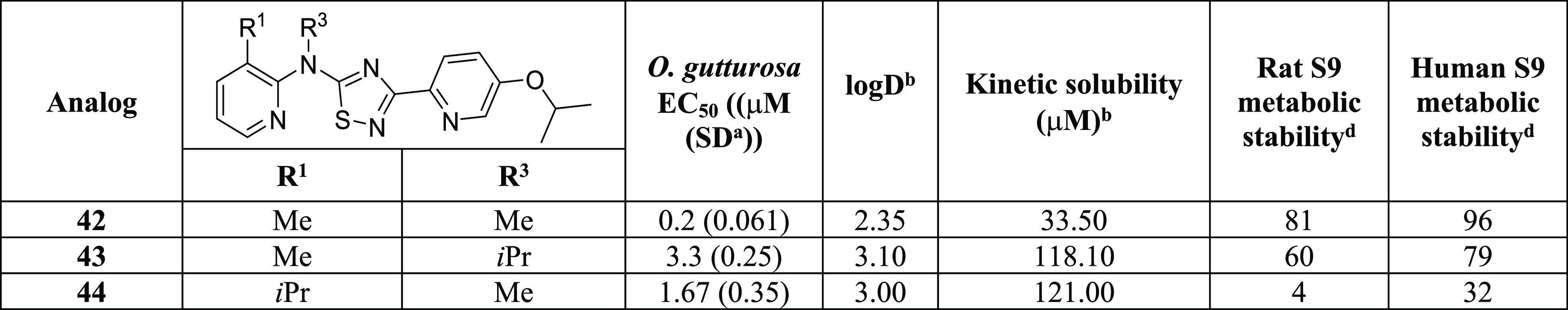
*Ex VivoO. gutturosa* Activity Addition of the R^3^ Substituent

a Standard deviation.

b The standard log *D* assay runs on 1 M concentration range using 10 mM DMSO stock solutions measured at pH 7.4.

c The standard kinetic solubility assay runs on 200 mM concentration range using 10 mM DMSO stock solutions.

d Liver S9 metabolic stability assay at 3 μM, % remaining @ 60 min.

**Scheme 2:**
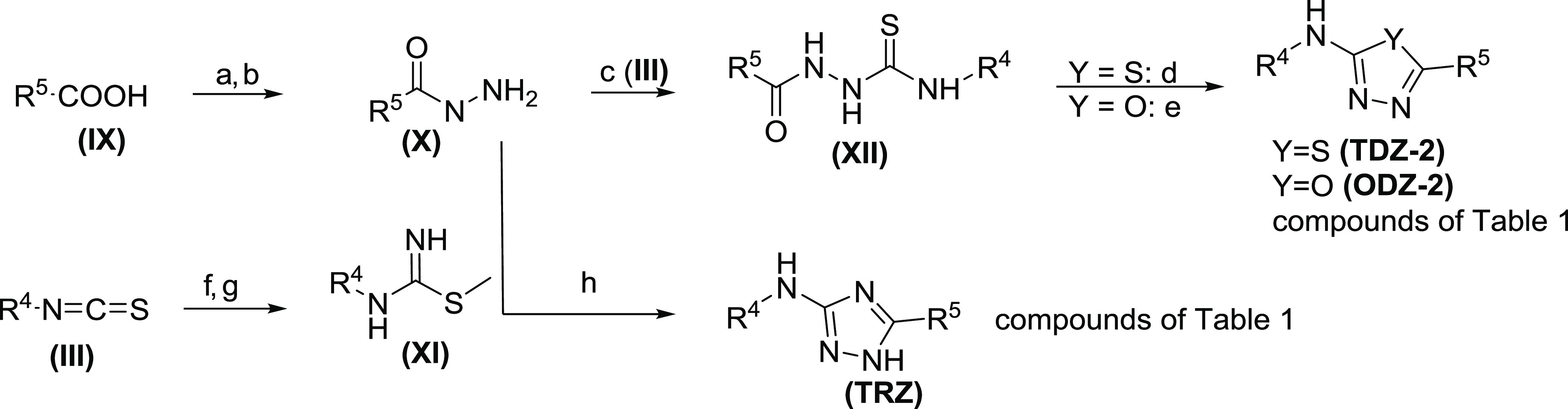
Synthesis of Di(pyridin-2-yl)-1,3,4-thia-oxa-diazol-5-amine and Di(pyridin-2-yl)-1,2,4-triazol-5-amine Reagents and conditions: (a) SOCl_2_, MeOH, 10–70 °C, 3 h, (b) N_2_H_2_ H_2_O, MeOH, 70 °C, 92%, 10 h, (c) (**III**), DCM, 30 °C, 19 h, 16–38% (d) pTsOH, toluene, 100 °C, 6 h, 32–51%, (e) DMSO, EDCI, 60 °C, 2 h, 24–35%, or IBX, DCM, Et_3_N, 0 °C, 1 h, 7%, (f) NH_4_OH, DCM, 30 °C, 1 h, 53%, (g) MeI, CH_3_CN, 40 °C, 16 h, 41%, (h) pyridine, 120 °C, 16 h, 10–29%.

Compounds containing the 1,3,4-thia (**TDZ-2**) and oxadiazole core (**ODZ-2**) in Table 1 are prepared by either dehydrative or desulfurative cyclization of thiosemicarbazide (**XII**) (Scheme 2). The thiosemicarbazide (**XII**) is formed by reacting the isothiocyanate (**III**) with hydrazide (**X**), which is prepared from commercially available carboxylic acid (**IX**). To obtain the 1,3,4-thiazdiazole (**TDZ-2**), thiosemicarbazide (**XII**) is treated with tosic acid, whereas for the formation of the 1,3,4-oxadiazole (**ODZ-2**), 1-ethyl-3-(3-dimethylaminopropyl)-carbodiimide (EDCI) is used.^20^ Alternatively, formation of the 1,3,4-oxadiazole can be accomplished via oxidative desulfurization using *o*-iodoxybenzoic acid (IBX).^21^ Analogs containing the 1,2,4-triazole core (**TRZ**) in Table 1 are synthesized by cyclocondensation of carbamimidothioate (**XI**) and hydrazide (**X**). Hydrazide (**X**) is prepared by esterification of carboxylic acid (**IX**) with SOCl_2_ and subsequent treatment with hydrazine hydrate.

## Results and Discussion

To alleviate the known bioactivation pathway at the C-5 position of the thiazole, we explored a variety of thiadiazoles, oxadiazoles, and triazoles. It had been shown that a number of heteroatoms in a ring, along with their position, have a major role in the metabolism of the heterocyclic ring systems.^22^

**Figure 2:**
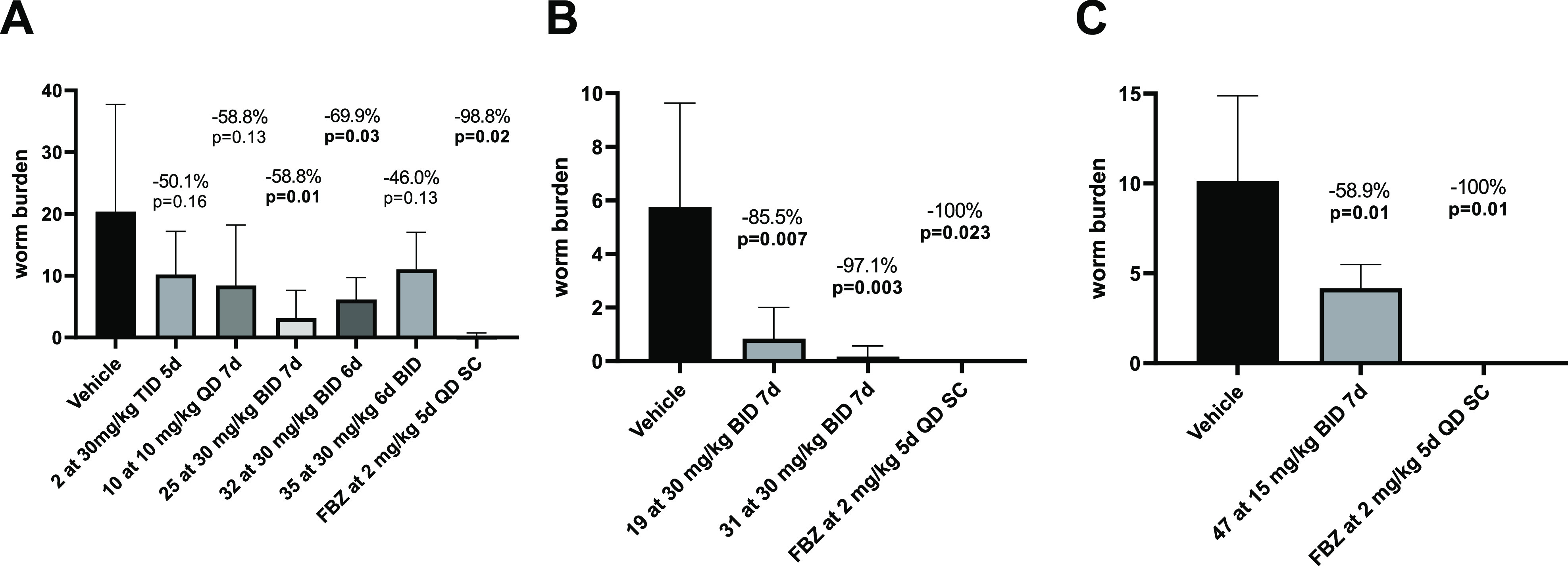
Adult worm burden in *L. sigmodontis*-infected female BALB/c mice and jirds after oral treatment with the candidates. Shown is the adult worm burden at 63–78 days after infection as mean plus standard deviation from the treatment groups and vehicle controls. (A) Mice have been naturally infected with *L. sigmodontis* for 35–37 days post infection and then treated orally. (B) Mice were subcutaneously infected with 40 infective L3 *L. sigmodontis* larvae and treated orally for 30–33 days post infection. (C) Jirds were subcutaneously infected with 40 infective L3 *L. sigmodontis* larvae and treated orally for 35–42 days post infection. Flubendazole (FBZ) was administered in all experiments subcutaneously (SC). Analysis was done using the unpaired two-tailed *t* test with *n* = 5–8 animals per group; FBZ was given to three to four animals.

The 1,2,4-thiadiazoles **2** and **3** maintained good activity as shown by reduction of *O. gutturosa* adult worm motility, whereas the 1,3,4-isomer, (**4**), was less potent by comparison. Overall, all thiadiazole isomers (**3**, **4**, and **5**) showed good metabolic stability (Table 1). A similar trend was observed for the oxadiazole cores (**6** and **7**) that showed good *ex vivo* activity and microsomal stability compared to the aminothiazoles. Both the thiadiazole core (**5**) and the triazole core (**8**) showed improved solubility when compared to the other heterocycles. Additionally, 1,2,4-thiadiazole analog (**2**) of the initial proof of concept compound (**1**) had improved pharmacokinetics (PK) (Table 5) and, although less potent, still demonstrated 50% reduction of adult worm burden *in vivo* (Figure 2A). Based on the consistency of activity^23^ and good preliminary PK and demonstration of *in vivo* efficacy, the 1,2,4-thiadiazole core (**TDZ-1**) was chosen as the primary focus for further exploration.

**Table 5:** Pharmacokinetic Parameters for Selected Compounds in Various Species

analog	species	route	dose (mg/kg)	*C*_max_ (μM)	*T*_max_ (h)	AUC_(0-inf)_ (μM·h)	Cl (mL/min/kg)	*V*_dss_ (mL/kg)	%F	brain/plasma [b]/[p]^a^	efflux ratio (ER)^b^
**1**	mouse^c^	PO^e^	30	0.69 ± 0.18	0.5 ± 0.0	1.58 ± 0.27					
**2**	mouse^c^	PO^e^	30	1.26 ± 0.29	1.3 ± 0.5	3.61 ± 0.86					NC
**10**	mouse^c^	PO^e^	30	8.26 ± 2.6	1.0 ± 0.6	26.0 ± 9.0					1.4
**19**	mouse^c^	PO^e^	30	2.08 ± 1.22	0.5 ± 0.0	6.23 ± 6.16					0.9^k^
**31**	mouse^d^	PO^e^	30	5.18	0.5	4.66 ± 0.54				1.10 (2 h)	1.0
	mouse^c^	PO^e^	10	0.65^h^	0.5^h^	0.94^h^			13^h^		
		IV^f^	2				62.9	3.01			
**25**	mouse^c^	PO^e^	30	2.05 ± 0.27	2.4 ± 3.8	20.0 ± 9.9					
**32**	mouse^c^	PO^e^	30	2.34 ± 0.52	0.5 ± 0.00	3.85 ± 0.76					
**35**	mouse^c^	PO^e^	30	0.21 ± 0.02	1.8 ± 1.7	1.73 ± 0.59					
**47**	mouse^c^	PO^e^	30	13.9^h^	0.50^h^	121^h^				0.08^h^	12
		PO^g^	10	5.27 ± 0.83	4.0 ± 2.8	68.1±NC^j^			57^i^		
		IV^f^	2				3.6 ± 0.22	0.74 ± 0.08			

a Brain to plasma ratio, based on AUC_0-*t*_.

b Permeability measured in a Madin Darby canine kidney cell, monolayer transfected with the multidrug resistance 1 (mdr1) gene encoding Pgp MDR1.

c Male CD-1 (*n* = 4).

d Female BALB/c mouse.

e As a suspension CMC/Tween 0.5% methyl cellulose/0.25% Tween.

f 15% DMA/50% PEG400/35% D5W.

g Suspension in 0.5% MC/0.25% Tween 80 in water.

h Data collected from non-serial sampling, no standard deviation reported.

i AUC extrap was above 23%.

j NC = not calculated.

k Measured in Caco-2 cells.

Having identified a replacement for the thiazole core, incorporation of appropriate substituents to achieve optimal physical, chemical, and pharmacokinetic properties was the next goal. SAR quickly revealed that the combination of small alkyl substituents (R^1^) on the A-ring, preferably in the 3-position, along with alkoxy groups (R^2^) in the 5-position of the B-ring showed a marked effect on inhibition of *O. gutturosa* adult worm motility (Table 2) and viability (data in the Supporting Information) while maintaining moderate solubility (10–50 μM) and good metabolic stability (rat and human liver S9 stability of ≥70% remaining at 60 min)^24^ (Table 2). While many of the new analogs containing the 1,2,4-thiadiazole core demonstrated sub-micromolar inhibition of *O. gutturosa* adult worm motility, the most potent compounds that exhibited *O. gutturosa*, EC_50_ < 100 nM, contained either a CH_3_, *i*Pr, or CF_3_ in the 3-position (R^1^) and an ether in the 5-position (R^2^) (i.e., **3**, **10**, **21**, **23**, **24**, and **27**). Incorporation of polarity into the B-ring was generally not detrimental, with some exceptions such as nitrile (**13**) and certain amides (**16**) showing loss of activity. However, combining the nitrile or amide with O*i*Pr (R^2^) on the B-ring (**15** and **17**) recovered some of the activity. Introduction of basic amines such as alkylated piperidines as either R^1^ or R^2^ improved both aqueous solubility (>10 μM) and metabolic stability while maintaining good activity. Unfortunately, the basic amine imparted a hERG (human ether-a-go-go-related gene) liability (**12** and **19**: hERG IC_50_ = 0.203 and 3.46 μM, respectively). Interestingly, the reported strategy of substituting piperidines with fluorine atoms to improve metabolism did not prove to be fruitful in this series of compounds. Compound **20** showed no improvement in metabolic stability; nevertheless, it did diminish the hERG activity (hERG IC_50_ > 30 μM) likely resulting from reduction of the nitrogen pK_a_.^25,26^ In general, compounds in this series did not harbor a hERG concern (data in the Supporting Information). The combination of a CF_3_ on either of the rings (A or B) with small alkyl substituents (R^1^) on the A-ring provided the most potent compounds, albeit at a cost of aqueous solubility. Compounds such as **23**, **24**, **35**, **37**, and **38** (*O. gutturosa* EC_50_ = 0.04, 0.05, 0.05, 0.05, and 0.02 μM, respectively) showed aqueous solubilities <1 μM or undetectable at the lowest measurable level. The ability to incorporate polarity on the A-ring did not necessarily impair activity and, in some instances, improved aqueous solubility. The 3-dimethyl amine as R^1^ provided good microsomal stability while maintaining potency. It is interesting to note that no lipophilicity requirement for activity was observed for this series of compounds, and they resided in a good property space (log *D*_7.4_ < 4) (Table 2). Ligand efficiency (LE, the measure of potency per atom) and ligand lipophilicity efficiency (LLE, the measure of potency relative to lipophilicity) were assessed to determine the relative efficiency for these compounds based on *O. gutturosaex vivo* data generated for these series. Based on the evaluation of these parameters, the compounds emerged in desirable chemical space LE = 0.35–0.45 and LLE = 3–5, which are consistent with optimized compounds (data in the Supporting Information).^27,28^ There also appeared to be no correlation of measured log *D*_7.4_ with metabolic stability; however, the general trend of increased lipophilicity resulting in poor solubility was observed.

Utilizing the preferred Me (R^1^) and O*i*Pr (R^2^) substituents, alternative six-membered N-containing heterocycles were evaluated on the 1,2,4-thiadiazole core (Table 3). Replacement of the A-ring with a pyrazine in **41** resulted in a ∼8-fold increase in solubility when compared to the pyridine analog **10**. Similarly, replacing the B-ring pyridyl with a pyrimidine (**40**) resulted in a ∼4-fold improvement in solubility over **10** (Table 3) albeit resulting in a decrease in potency. The additional nitrogen in either A- or B-rings did not result in any improvement in activity or offer any additional improvements in microsomal stability.^29^

Derivatization of the NH with small alkyl groups (R^3^) in compounds such as **43** and **44** did not abolish activity and provided good solubility despite the increased log *D*_7.4_ (Table 4)_._ The introduction of an R^3^ substituent likely disrupts planarity leading to improved aqueous solubility.^30^

*O. volvulus* is the parasite responsible for onchocerciasis, and e*x vivo* testing against *O. volvulus* is only possible by recovering L3 infective larvae via the dissection of black flies and inducing *ex vivo* molting into L5 larvae.^5^ This is labor-intensive, low throughput, and not ideal for screening or lead optimization; therefore, only a limited subset of compounds could be tested. Some of the initial potent compounds against *O. gutturosa*, **11**, **31**, **32**, **23**, and **27**, demonstrated 100% motility reduction of *O. volvulus* L5 larvae at 1 μM confirming antiparasitic activity against the human parasite and validating the series effectiveness at targeting the adult stage of the human disease, onchocerciasis (data in the Supporting Information).

At this point in the study, we chose to evaluate several potent compounds for *in vivo* pharmacokinetics and efficacy. When dosed orally at 30 mg/kg, select compounds had mouse plasma exposures that provided sufficient exposure and time above the EC_50_ when dosed twice per day (BID) for efficacy evaluation *in vivo* (Table 5). Prioritization of compounds was based on *in vitro* potency and pharmacokinetic profile. These selected compounds were assessed in female BALB/c mice as oral BID regimens of 30 mg/kg for treatment times ranging from 1 to 7 days with the treatment starting at 30–37 days post infection (dpi), a time point at which adult worms had developed, and necropsies were performed at 63–78 dpi. Reduction of the adult *L. sigmodontis* worm burden was measured in comparison to the untreated/vehicle-treated control. In addition to the first thiadizaole compound tested *in vivo* (**2**, 30 mg/kg TID regimen for 5 days), a spread of efficacy was observed across the compounds tested (Figure 2). Compound **10**, 30-fold more potent and with a 7-fold improved exposure, AUC_(0-inf)_ = 26 μM·h, over **2** showed a 59% reduction of adult *L. sigmodontis* worms, when dosed at 10 mg/kg QD for 7 days. It should be noted that all compounds were tested in mice at a concentration of 30 mg/kg except for compound **10**, which was dosed at a concentration of 10 mg/kg due to tolerability issues. Thus, even though compound **10** showed marked efficacy at a lower dose in comparison to the other compounds, further *in vivo* analysis was not pursued.

Compound **19**, containing a basic nitrogen, demonstrated 86% reduction of adult worm burden at 30 mg/kg given BID for 7 days. Unfortunately, **19** contained a hERG liability (hERG IC_50_ = 3.46 μM) and would not progress any further. Compound **35**, although potent (*O. gutturosa* EC_50_ = 0.05 μM), showed marginal reduction of adult worm burden (46%) at 30 mg/kg given BID for 6 days, which could be indicative of the poor exposure and poor solubility associated with the compound (AUC_(0-inf)_ = 1.7 μM·h). Compound **25** showed reduction of adult worm burden (59%) given at 30 mg/kg BID for 7 days, which could be attributed to the compound’s AUC_(0-inf)_ = 20 μM·h and perhaps the delayed *T*_max_ (2.4 h) relative to the other analogs (*T*_max_ = 0.5 h), providing a more sustained coverage. Containing polar functionality as R^1^ of the A-ring, compounds **31** and **32** demonstrated remarkable reduction of adult worm burden at 30 mg/kg BID, 97% reduction with compound **31** with 6 days of dosing and 70% reduction with compound **32** with 7 days of dosing. Subsequently, **31** was evaluated in chronically *L. sigmodontis*-infected microfilariae-positive jirds, confirming macrofilaricidal efficacy (71% reduction when dosed at 2.5 mg/kg SC for 7 days QD, data in the Supporting Information). The *in vivo* efficacy of compounds **31** and **32** appears to be driven by *C*_max_ as both compounds have a relatively modest pharmacokinetic profile in rodents. From the compounds tested *in vivo*, it appeared that analogs containing an ether, preferably the O*i*Pr (R^2^) substituent in the 5-position and the methyl or di-methyl amine (R^1^) substituents in the 3-position, trended toward *in vivo* efficacy.

Based on ADME (absorption, distribution, metabolism, and excretion), physicochemical properties, and observed efficacy, **31** emerged as one of the most attractive compounds exhibiting acceptable plasma clearance and a low volume of distribution (Vss) across multiple species (Supporting Information). Showing no *in vitro* safety concerns (Table 7), it was progressed into *in vivo* safety evaluation studies. During exploratory toxicological evaluation, CNS (central nervous system)-related observations were noted, and it was determined to halt further development of **31**. It was shown that **31** crosses the blood brain barrier (BBB) efficiently (brain to plasma ratio: 1.1 (at 2 h)); hence, we focused efforts on compounds that would not cross the BBB, thereby limiting brain exposure. Since the mechanism of action of this series of compounds is not known, the best approach was to design analogs that would limit exposure within the brain.

Our approach was to target compounds with efflux ratio (ER) >2.5 in the MDCK cell line transfected with MDR1 (multidrug resistance-1 transfected Madin Darby canine kidney cell line). This MDCK/MDR1 cell line has been shown to identify compounds that are likely to be substrates for P-glycoprotein 1 (Pgp) and are unlikely to cross the BBB as an ER >2.5 is consistent with a brain to plasma ratio [b]/[p] of <0.2 and limited brain exposure.^31,32^ It is suggested that Pgp efflux is much greater in compounds containing more than two to three hydrogen bond donors (HBDs).^33,34^ Our goal was to impart structural elements designed to impart Pgp recognition.

Screening in the MDCK/MDR1 cell line identified compounds that had Pgp substrate potential, indicated by the high efflux ratio (Table 6). Compound **47**, containing a methyl acetamide at the 3-position (R^1^), was rapidly identified with a high efflux ratio of 12 while maintaining good permeability, *P*_appA-B_ = 3.9 × 10^–6^ cm/s, and consequently, **47** showed a brain to plasma ratio of 0.08, suggesting limited brain exposure (Table 5).

**Table 6:**
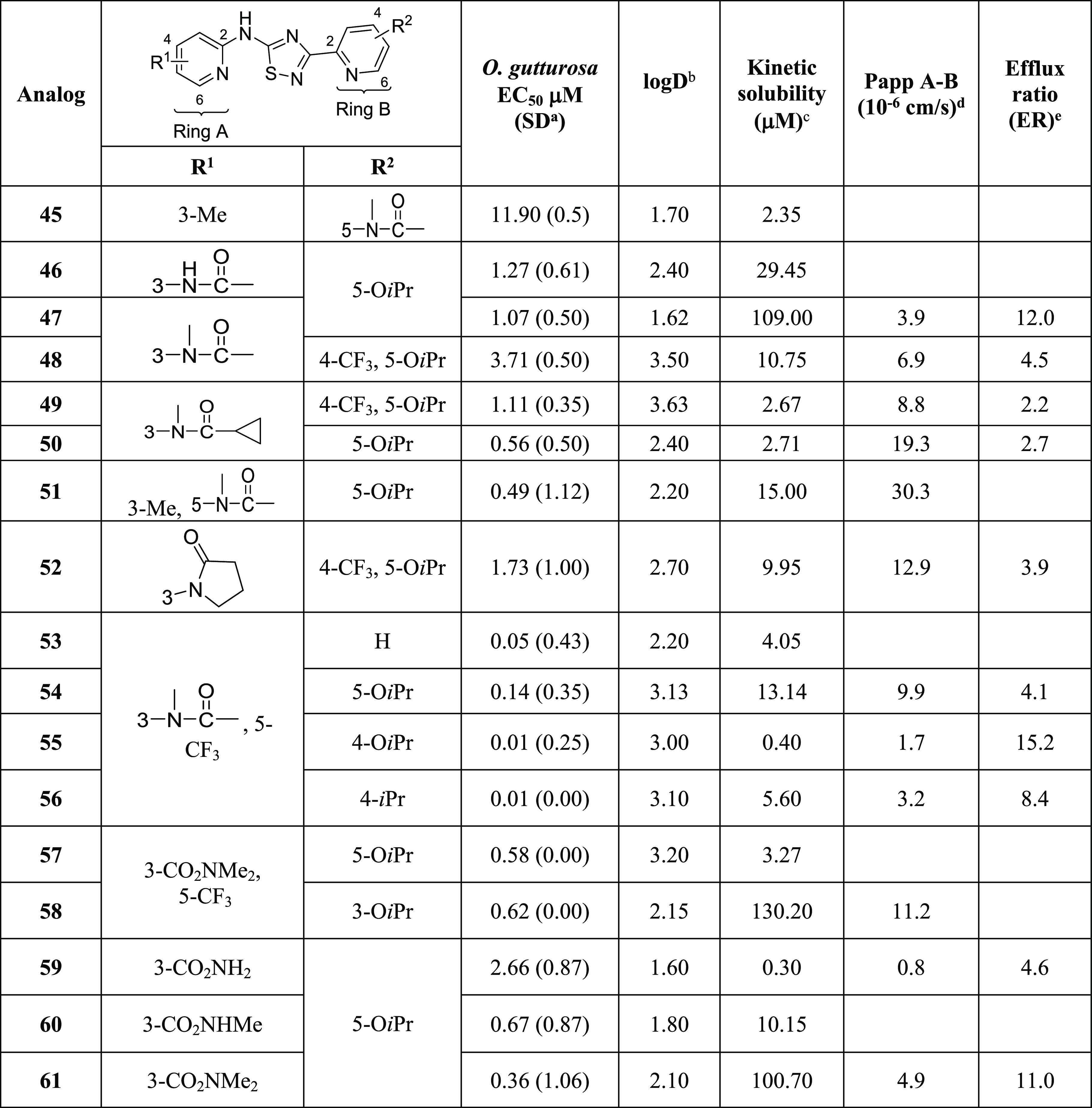
*Ex VivoO. gutturosa* Activity, Permeability, and Pgp Efflux

a Standard deviation.

b The standard log *D* assay runs on 1 M concentration range using 10 mM DMSO stock solutions measured at pH 7.4.

c The standard kinetic solubility assay runs on 200 mM concentration range using 10 mM DMSO stock solutions.

d Permeability measured in a Madin Darby canine kidney cell, monolayer transfected with the multidrug resistance 1 (mdr1) gene encoding Pgp MDR1.

e Efflux ratios (ER) are determined without and with valspodar.

We focused new analogs on increasing polarity to limit brain exposure (Table 6). Cyclizing the acetamide (R^1^) (**52**) resulted in good permeability and solubility with an ER = 3.9, whereas the introduction of the nitrogen-linked acetamide (R^2^) on the B-ring (**45**) resulted in complete loss of activity. Exchanging the methyl on acetamide increased activity (**50**), although the small increase in lipophilicity resulted in decreased aqueous solubility and decreased efflux ratio (ER = 2.7). Compound **50** with ER = 2.7 and *P*_appA-B_ = 19.3 × 10^–6^ cm/s still exhibited limited brain exposure, [b]/[p] = 0.11.

Introduction of carbon-linked amides in the 3-position of R^1^ showed a range of activity and solubility. The primary amide (**59**) had no detectable solubility, poor permeability (*P*_appA-B_ = 0.79 × 10^–6^ cm/s), and less activity (*O. gutturosa* EC_50_ = 2.6 μM), whereas a tertiary amide (**61**) had improved solubility (101 μM), potency (*O. gutturosa* EC_50_ = 0.36 μM), and permeability (*P*_appA-B_ = 4.9 × 10^–6^ cm/s and ER = 11). Combinations of the methyl acetamide with CF_3_ provided some of the most potent compounds, **56** (*O. gutturosa* EC_50_ = 0.01 μM) and **55** (*O. gutturosa* EC_50_ = 0.01 μM). Interestingly, both **56** and **55** contained O*i*Pr (R^2^) groups at the 4-position rather than the previously observed optimal 5-position. These compounds also had good permeability and efflux ratios suggesting limited brain penetration, **56** (ER = 8.4) and **55** (ER = 15). Disappointingly, **55** had an aqueous solubility of <1 μM. Addition of a CF_3_ (R^2^) with the 5-O*i*Pr on the B-ring, **34**, did not add any benefits over **47** and was 10-fold less soluble with ER = 4.5. The addition of an acetamide in the 5-position of an already 3-Me (R^1^) substituted A-ring resulted in ∼8-fold improved solubility, **51** compared to **10**. Most of the analogs within this chemical series shared nearly identical properties as the total counts of HBDs and total polar surface areas (TPSA) remain constant; therefore, the significant changes in their Pgp recognition, efflux ratios, and/or permeability observed imply that further analysis of factors such as H-bond strength and intermolecular H-bonding may be required.

Compounds **47** and **50** showed 100% reduction of*O. volvulus* L5 larvae motility and viability at 1 μM confirming both antiparasitic activity against the human parasite and effectiveness of these new analogs (data in the Supporting Information). Finally, compound **47** was tested in*L. sigmodontis*-infected jirds and showed a significant reduction of 59% when dosed at 15 mg/kg orally for 7 days BID (Figure 2C).

Having identified compounds with limited brain exposure, further profiling was needed to determine whether the inhibition of Pgp would be of consequence for further development. Compound **47** still maintained high permeability in the MDCK wild-type cell line treated with elacridar (a PgP and BCRP inhibitor),^31^**47***P*_appA-B_ = 30 × 10^–6^ cm/s. Compound **47** still maintained good oral exposure having slow plasma clearance in the mouse, 3.6 mL/min/kg. Overall, the brain-limiting analogs maintained the promising ADME and safety profile of the previous analog.

*In vitro* cytotoxicity of select compounds was tested in a “normal” proliferating transformed human liver epithelial (THLE-2) cell line.^35^ Compounds were incubated in THLE-2 cells for 24 and 72 h, and adenosine triphosphate (ATP) levels were used as an indicator of cell viability. Compounds **47** and **31** (Table 7) did not exhibit significant cellular cytotoxity (additional compounds in the Supporting Information).

**Table 7:** *In Vitro* Safety Properties

*in vitro* ADME and safety	**31**	**47**
Plasma Protein Binding % (h/r)	>99/>99	90.7/95
Cytochrome P450s IC_50_ (μM) (3A4, 1A2, 2D6, 2C9, 2C19, 2C8)	22, >30, >30, 5.5, 20, 7.1	>30, >30, 30, 5.7, 29, >30
THLE^a^ (cytotoxicity) ATP LC_50_ (μM)	36	106
hERG binding^b^ (μM)	11.4	>30
CEREP^c^ receptor, ion channel, transporter panel (binding at 10 μM)	5/76	1/76
	>70%	>50%
Kinase selectivity panel at 3 μM	0/259	0/259
AMES-II/mini-AMES^d^^,^^e^	negative	negative
micronucleus^f^		negative

a ″Normal” proliferating transformed human liver epithelial (THLE-2) cell line.

b Cloned hERG potassium channels (encoded by the KCNH2 gene and expressed in HEK293 cells).

c Secondary pharmacology targets were screened in the Eurofins CEREP non-kinase panel.

d Kinase, selectivity was evaluated in Thermo Fisher Scientific’s SelectScreenTM panel.

e Non-mutagentic TA98, TA100, TA1535, TA97, and WP2 uvrA strains either in the presence or absence of metabolic activation (S9 fraction).

f TK6 cells, in the presence and absence of Aroclor 1254-induced rat liver S9 activation.

In line with developing potential drug candidates, compounds were examined for additional ADME and safety properties (Table 7). Although having moderate solubilities, compounds displayed high permeability and moderate to high binding to human plasma proteins. To address any potential off target activity and potential safety concerns, compounds were screened against a panel of 70 secondary pharmacology targets^36^ and up to 259 kinases.^37^ Although compounds **47** and **31** contain the amino pyridine motif, they did not inhibit any kinases.^38^ Only a few targets were observed at 10 μM in the secondary pharmacology binding assay. Low inhibition of hERG was observed, and compounds did not inhibit CYP3A4 and were not mutagenic in an AMES test (Table 7).

## Conclusions

The initially identified amino-thiazole compound (**1**) showed a significant reduction of adult *L. sigmodontis* worm burden and provided a basis for lead optimization efforts. Through several modifications, a series of substituted di(pyridin-2-yl)-1,2,4-thiadiazol-5-amine compounds were discovered having *O. gutturosa* EC_50_ < 100 nM and 100% inhibition (1 μM) against the human parasite *O. volvulus* L5 stage larvae. The series of substituted di(pyridin-2-yl)-1,2,4-thiadiazol-5-amine compounds showed a trend of reducing overall adult worm burden in the *L. sigmodontis* mouse and jird infection model. Additionally, other cores (i.e., TRZ and other TDZ regioisomers) from this series demonstrated favorable physical chemical and pharmacokinetic properties and potencies, which warrant further exploration should resources become available. However, there appears to be a disconnect in the *ex vivo* and *in vivo* efficacy correlation (EVIVC) that can possibly be attributed to several discrepancies, such as the fact that the *L. sigmodontis* and *O. gutturosa* rodent model used for screening are surrogate filarial nematodes to model human filarial diseases. While *O. gutturosa* is used as the screening surrogate of *O. volvulus*, this series of compounds has demonstrated activity against the *L. sigmodontis* parasite.^23^ Last, the location of the parasite (thoracic cavity for *L. sigmodontis* versus subcutaneous and deep nodules for *O. volvulus*) might be of some consequence with regard to compound tissue distribution in this model.

Overall, this series of compounds maintains good ADME and physiochemical properties and these compounds are considered to be BCS Class II (good to high permeability yet poor solubility).^39^ The brain-limiting compound **47** demonstrated efficacy in reducing adult worm burden in the jird *L. sigmodontis* infection model (Figure 2C), confirming macrofilaricidal efficacy.^40^ To date, it is not clear which preclinical model predicts clinical efficacy nor adverse reactions related to the death of filarial parasites. In the future, clinical trials should be used to back translate efficacy in filarial preclinical models. However, based on our observation that adult worm clearance takes several weeks, we do not anticipate severe adverse events.

## Experimental Section

### *O. gutturosa* Adult Worm *Ex Vivo* Assay

Activity against *O. gutturosa* adult male worms was determined using reported procedures.^7^ The EC_50_ values were calculated using Microsoft Office Excel (2010) data analysis software. The tested compound is considered active when a motility reduction of ≥50% is observed by comparison to the untreated controls. *O. gutturosa* adult male worms were obtained postmortem from freshly slaughtered cattle. The worms were dissected from the nuchal ligament connective tissues of naturally infected cattle in Gambia, West Africa; the material was purchased from the International Trypanotolerance Centre, Banjul, from local butchers for use in this study. The adult worms were transferred individually to each well of a sterile 24-well (2 mL) plate (Thermo Fisher Scientific, UK) and maintained for at least 24 h in culture before use. The medium used was a minimum essential medium (MEM) with Earl’s salts and l-glutamine (Life Technologies Ltd., UK) supplemented with 10% heat inactivated newborn calf serum (Life Technologies Ltd., UK), 200 units/mL penicillin, 200 μg/mL streptomycin, and 0.5 μg/mL amphotericin B (Life Technologies Ltd., UK). Only normally active worms were used for the test, and all assays were conducted at 37 °C and 5% CO_2_. The positive control drug used was Immiticide (melarsomine dihydrochloride, Merial, USA). Known amounts of compounds were solubilized in 1 mL of DMSO. All stock solutions were allowed to stand at room temperature prior to use. The test items (four worms/drug concentration) were compared to untreated controls (six worms) and the positive control (two worms/drug concentration). Drug efficacy was assessed by the measurement of mean worm motility scores on a scale of 0 (immotile) to 10 (maximum) every 24 h, terminating at 120 h, using an Olympus inverted microscope. The results are reported as a percentage of the maximum score obtainable (100%) by calculating the motility index scores. The test drug is considered active when a motility reduction of ≥50% is observed by comparison to the untreated controls. The IC_50_ value indicates the drug concentration that inhibited the motility by 50%. In addition, biochemical evaluation of worm viability was assessed using MTT/formazan colorimetry. The MTT assay was carried out after the last motility reading (120 h). Single intact worms were placed in each well of a 48-well plate (Thermo Fisher Scientific, UK) containing 0.5 mL of 0.5 mg/mL MTT (Sigma, UK) in PBS solution and then incubated for 30 min at 37 °C. The worms were removed, blotted carefully, and individually transferred to separate wells of a 96-well microtiter plate, each containing 200 μL of DMSO to solubilize the formazan. After 1 h, the plate was gently agitated to disperse the color evenly and the absorbance value (optical density, OD) of the resulting formazan solution was determined at 490 nm using an absorbance microplate reader (Biotek ELx800, Thermo Fisher Scientific, UK). The results are expressed as the mean OD per drug concentration. Inhibition of formazan formation is correlated with worm damage or death.

### *O. volvulus* L5 *Ex Vivo* Assay

*O. volvulus* larvae (L4) were prepared using cryopreserved *O. volvulus* L3 cultured in a medium containing 1:1 NCTC-109 and IMDM (Iscove’s modified Dulbecco’s medium) supplemented with Glutamax (1×) and 2× Antibiotic-Antimycotic (Life Technologies). This culturing medium was then added in transwell 24-well plates containing human umbilical vein endothelial cell (HUVEC) monolayers, containing the L4 larvae. A week prior to conducting an assay, the cultured *O. volvulus* larvae (L5) were transferred into a Petri dish containing fresh medium. Aliquots of 8–10 worms were retrieved from the Petri dish and placed inside new transwells over newly seeded HUVEC monolayers in the medium described above. After the transfer of *O. volvulus* L5 to the plates, the test compounds were dissolved in DMSO, added to each well, and incubated at 37 °C in a 5% CO_2_. The media containing freshly prepared compounds were partially replaced every 2–3 days for 14 days. After 14 days, the media were completely replaced. The untreated control contains the media described above with a final concentration of 0.05% DMSO. The motility was measured every 2–3 days with the following scale: 100% motility, constant coiling movement; 75% motility, slower coiling; 50% motility, slow and intermittent movement; 25% motility, very slow movement or twitching; and 0% motility, no movement. The motility was measured visually by two operators. On day 28, the viability of *O. volvulus* L5 was measured by MTT. Following washout and incubation during 1 h in PBS under 5% CO_2_, worms were observed under an inverted microscope. Worms were considered dead if no staining or less than 50% within the worm was observed. Otherwise, if worms stained blue or more than 50% stained, then they are considered alive. All test treatments were performed in duplicates. Significance was determined using a *t* test.

### Mouse Pharmacokinetics

All animal experiments were approved by the Institutional Animal Care and Use Committee (IACUC) of Bristol Myers Squibb. All studies were conducted in fed animals (male CD-1 mice) by Charles River Laboratories (Wilmington) Inc. Oral dosing (at specified doses, 10 mL/kg) was as a suspension in 0.5% carboxymethylcellulose, and 0.25% Tween 80 in water or in 0.5% methyl cellulose/0.25% Tween 80 in water and intravenous dosing (at specified doses) was a solution in 15% dimethylacetamide (DMA /50% polyethylene glycol (PEG400)/35% DSW. Plasma samples (at 0.5, 1.5, 3, 5, and 8 h) were analyzed by LC–MS/MS, and the PK parameters were calculated using Phoenix WinNonlin software.

### *In Vivo* Efficacy Using *L. sigmodontis*

All experimental procedures were performed in accordance with EU directive 2010/63/EU and the relevant national legislation, the “Décret no 2013–118, 1er février 2013, Ministère de l’Agriculture, de l’Agroalimentaire et de la Foret”, national license number 75–1415. Animal protocols were approved by the ethical committee of the MNHN (Comité Cuvier, license: 68–002) and by the “Direction de′partementale de la cohésion sociale et de la protection des populations” (DDCSPP) (no. C75-05-15). The animal experiments done at IMMIP were approved by the Landesamt für Natur, Umwelt und Verbraucherschutz, Cologne, Germany (AZ 84-02.04.2015.A507). Female BALB/c mice (obtained from Janvier for IMMIP and Envigo for MNHN) were infected with *L. sigmodontis* at 6–8 weeks of age by natural exposure to the infected mite vector (IMMIP) or by subcutaneous injection of 40 infective L3 larvae (MNHN).^7^ Treatments were initiated at 30–37 days post-infection (dpi) in mice as previously described.^7^ Doses were given by oral gavage (10 mL/kg body weight) in milligrams of drug substance per kilograms of body weight of animals and are indicated in the Results and Discussion section and legend of Figure 2. Mice were sacrificed at 63–78 dpi. Female gerbils *Meriones unguiculatus* were infected at 6–8 weeks of age by natural infection via the mite vector at IMMIP or subcutaneously with 40 L3 larvae at the MNHN. At the IMMIP, microfilariae-positive animals were subcutaneously treated once per day with compound **31** at 2.5 mg/kg for 7 days (*n* = 8). At the MNHN, jirds were treated after the development of adult worms for 30 days post infection. Treatment was done orally for 7 days BID with 15 mg/kg compound **47** (*n* = 6). Controls received vehicle treatments (*n* = 8). As positive control, gerbils and mice were subcutaneously treated with 2 mg/kg flubendazole for 5 days (*n* = 3–4). At the IMMIP, necropsies were performed 12 weeks after treatment started. The recovered *L. sigmodontis* adult worms were counted and analyzed by light microscopy to identify males and females. At the MNHN, necropsy was performed 70 days post infection. Differences in the protocols used between MNHN and IMMIP for the *L. sigmodontis* infection of jirds were caused by the COVID-19 restrictions in France that prevented long-term infections. Adult worm burden reduction was calculated by comparing the mean adult worm burden in vehicle controls to the adult worm burden in the treatment groups. Analysis was done using GraphPad Prism version 9.3.1 and an unpaired two-tailed *t* test with five to eight animals per group.

### General

Compounds were named using ChemDraw Ultra 18.2. All materials were obtained from commercial sources and used without further purification unless otherwise noted. All air-sensitive reactions were carried out under a positive pressure of an inert nitrogen atmosphere. The reactions were monitored by thin-layer chromatography (TLC) carried out on silica gel plates (0.25 mm thick, GF254), and visualization was achieved by UV light. ^1^H NMR (proton nuclear magnetic resonance) spectra and low-resolution electrospray ionization–MS (ESI–MS) were used for characterization. ^1^H NMR spectra were measured on a Bruker or Varian 400 MHz spectrometer, and chemical shifts were reported in parts per million (δ, ppm) relative to the internal reference tetramethylsilane (Me_4_Si). NMR spectra were acquired in CDCl_3_, CD_3_OD, or DMSO-*d*_6_. ^1^H NMR data are reported as follows: chemical shift [multiplicity (s = singlet, d = doublet, t = triplet, q = quartet, dd = doublet of doublets, m = multiplet, brs = broad singlet), *J* = coupling constant(s) (Hz), integration]. Low-resolution ESI–MS data were recorded on a Shimadzu LCMS-2020 mass spectrometer using an ESI source. LCMS conditions were included but not limited to the two as follows: Shimadzu LCMS-2020, Kinetex @ 5 μm EVO C18 30 × 2.1 mm, 5–95% MECN (0.01875% TFA) in water (0.0375% TFA), 1.5 min run, flow rate, 1.5 mL/min, UV detection (λ = 220 and 254 nm) or Shimadzu LCMS-2020, Kinetex EVO C18 2.1 × 30mm, 5 μm, 5–95% MECN in water (0.025% NH_3_·H_2_O), 1.5 min run, flow rate, 1.5 mL/min, UV detection (λ = 220, 254 nm). All the final compounds were purified to >95% purity. Conditions were as follows: Agilent 1200 LC and Agilent 6110 MSD, Phenomenex Gemini 3 μm NX-C18, 4.6 × 50 mm, 0–100%, A: 95% H_2_O + 5% ACN (0.1% FA), B: 5% H_2_O + 95% ACN (0.1% FA), 6 min run, flow rate, 1.0 mL/min, UV detection (λ = 214, 254 nm). Flash column chromatography was carried out using prepacked silica cartridges (from 4 up to 120 g) from Agela and eluted using a Biotage Companion system. All final compounds were purified to have purity higher than 95% by reverse-phase high-performance liquid chromatography (HPLC), normal phase flash chromatography, or crystallization. Reported yields were unoptimized. Calculations were carried out and plotted using Dotmatics Vortex v2016.10.56814.

### General Procedure A for the Synthesis of Compounds in Tables 1–3 and 6

#### 3-(5-Isopropoxypyridin-2-yl)-*N*-(3-methylpyridin-2-yl)-1,2,4-thiadiazol-5-amine (**10**)

##### 5-Isopropoxypicolinonitrile

To a solution of propan-2-ol (1.18 g, 19.7 mmol, 1.49 mL) in *N*,*N*-dimethylformamide (40 mL) was added sodium hydride (983 mg, 24.6 mmol, 60% purity) at 0 °C under nitrogen. The mixture was stirred at 25 °C for 30 min, and 5-fluoropicolinonitrile (2.00 g, 16.4 mmol) was added to the reaction mixture at 0 °C under nitrogen. The mixture was stirred at 25 °C for 4 h and then poured into ice water. The aqueous phase was extracted with EtOAc, and the organic layers were washed with brine, dried over sodium sulfate, filtered, and concentrated to give a residue. The residue was purified by column chromatography to give 5-isopropoxypicolinonitrile (2.10 g, crude).

##### 5**-**Isopropoxypicolinimidamide

To a solution of 5-isopropoxypicolinonitrile (500 mg, 3.08 mmol) in methanol (25 mL) was added sodium methanolate (16.6 mg, 308 μmol), and the mixture was stirred at 25 °C for 8 h. Ammonium chloride (330 mg, 6.16 mmol) was added to the mixture and heated at 70 °C for 4 h. The mixture was concentrated under reduced pressure to give a residue, which was triturated with DCM (30 mL). The mixture was filtered, and the filter cake was dried to remove the solvent to give 5-isopropoxypicolinimidamide hydrochloride (800 mg, crude, HCl).

##### 2-Isothiocyanato-3-methylpyridine

To a solution of thiophosgene (5.32 g, 46.2 mmol) in DCM (80 mL) was added a solution of 3-methylpyridin-2-amine (5 g, 46.2 mmol) in DCM (50 mL) at −5 °C under a nitrogen atmosphere. The mixture was stirred at 25 °C for 3 h, and saturated sodium bicarbonate was added to the mixture. The mixture was extracted with DCM, and the combined organic layers were dried over sodium sulfate, filtered, and concentrated under reduced pressure to give a residue. The residue was purified by silica gel column chromatography to give 2-isothiocyanato-3-methylpyridine (1.00 g, 6.66 mmol, 14% yield).

##### 5-Isopropoxy-*N*-((3-methylpyridin-2-yl)carbamothioyl)-picolinimidamide

To a solution of 2-isothiocyanato-3-methylpyridine (557 mg, 3.71 mmol) and Et_3_N (3.75 g, 37.1 mmol, 5.14 mL) in DCM (15 mL) and acetone (15 mL) was added 5-isopropoxypicolinimidamide hydrochloride (800 mg, 3.71 mmol, HCl). The mixture was stirred at 25 °C for 20 h, and then, the reaction mixture was concentrated under reduced pressure, diluted with water, and extracted with EtOAc. The combined organic layers were washed with brine, dried over sodium sulfate, filtered, and concentrated under reduced pressure to give 5-isopropoxy-*N*-((3-methylpyridin-2-yl)carbamothioyl)picolinimidamide (350 mg, crude).

##### 3-(5-Isopropoxypyridin-2-yl)-*N*-(3-methylpyridin-2-yl)-1,2,4-thiadiazol-5-amine

To a solution of 5-isopropoxy-*N*-((3-methylpyridin-2-yl)carbamothioyl)picolinimidamide (350 mg, 1.06 mmol) in THF (10 mL) was added diisopropyl azodiformate (DIAD) (279 mg, 1.38 mmol). The mixture was stirred at 25 °C for 20 h. The product was isolated and purified via standard methods to give 3-(5- isopropoxypyridin-2-yl)-*N*-(3-methylpyridin-2-yl)-1,2,4-thiadiazol-5-amine (126 mg, 0.385 mmol, 36% yield, 99.3% purity). ^1^H NMR (400 MHz, DMSO-*d*_6_) δ 8.35–8.29 (m, 2H), 8.17 (d, *J* = 8.8 Hz, 1H), 7.67 (d, *J* = 7.4 Hz, 1H), 7.51 (dd, *J* = 8.8, *J* = 2.9 Hz, 1H), 7.03 (dd, *J* = 7.2, *J* = 5.1 Hz, 1H), 4.81–4.75(m, 1H), 2.40 (s, 3H), 1.33–1.32 (m, 6H); MS (ESI): *m*/*z* 328.2 [M + 1]^+^.

##### *N*,3-Di(pyridin-2-yl)-1,2,4-thiadiazol-5-amine (**9**) (100% Purity)

^1^H NMR (400 MHz, DMSO-*d*_6_) δ ppm 12.31–12.51 (m, 1 H), 8.65–8.79 (m, 1 H), 8.41–8.55 (m, 1 H), 8.15–8.28 (m, 1 H), 7.91–8.00 (m, 1 H), 7.81–7.90 (m, 1 H), 7.44–7.52 (m, 1 H), 7.14–7.21 (m, 1 H), 7.05–7.13 (m, 1 H); MS (ESI): *m*/*z* 256.1 [M + 1]^+^.

##### *N*,*N*-Dimethyl-2-(5-((3-methylpyridin-2-yl)amino)-1,2,4-thiadiazol-3-yl)isonicotinamide (**11**) (57% Yield, 99% Purity)

^1^H NMR (400 MHz, DMSO-*d*_6_) δ 11.86 (s, 1H), 8.77 (d, *J* = 4.9 Hz, 1H), 8.33 (d, *J* = 4.3 Hz, 1H), 8.19 (s, 1H), 7.69 (d, *J* = 7.3 Hz, 1H), 7.49 (dd, *J*_1_ = 4.8, *J*_2_ = 1.4 Hz, 1H), 7.05 (dd, *J*_1_ = 7.2, *J*_2_ = 5.1 Hz, 1H), 3.03 (s, 3H), 2.93 (s, 3H), 2.41 (s, 3H); MS (ESI): *m*/*z* 341.3 [M + 1]^+^.

##### *N*,*N*-Dimethyl-6-(5-((3-methylpyridin-2-yl)amino)-1,2,4-thiadiazol-3-yl)nicotinamide (**18**) (52% Yield, 99% Purity)

^1^H NMR (400 MHz, DMSO-*d*_6_) δ 11.92 (brs, 1H), 8.75 (d, *J* = 1.6 Hz, 1H), 8.36–8.25 (m, 2H), 8.01 (dd, *J*_1_*=* 8.0, *J*_2_ = 2.1 Hz, 1H), 7.69 (d, *J* = 7.2 Hz, 1H), 7.05 (dd, *J*_1_*=* 7.2, *J*_2_ = 5.0 Hz, 1H), 3.07–2.95 (m, 6H), 2.42 (s, 3H); MS (ESI): *m*/*z* 341.1 [M + 1]^+^.

##### 3-(3-Methyl-2-pyridyl)-*N*-(5-pyrrolidin-1-yl-2-pyridyl)-1,2,4-thiadiazol-5-amine (**21**) (21% Yield, 97% Purity)

^1^H NMR (400 MHz, CD_3_OD) δ 8.47 (d, *J* = 3.9 Hz, 1H), 7.86–7.77 (m, 2H), 7.41 (dd, *J*_1_ = 7.8, *J*_2_ = 4.8 Hz, 1H), 7.16 (dd, *J*_1_ = 8.9, *J*_2_ = 2.9 Hz, 1H), 7.02 (d, *J* = 8.8 Hz, 1H), 3.35–3.32 (m, 4H), 2.55 (s, 3H), 2.11–2.01 (m, 4H); MS (ESI): *m*/*z* 339.1 [M + 1]^+^.

##### 3-(4-Methylpyridin-2-yl)-*N*-(3-(trifluoromethyl)pyridin-2-yl)-1,2,4-thiadiazol-5-amine (**26**) (10% Yield, 99% Purity)

^1^H NMR (400 MHz, DMSO + D_2_O) δ 8.71 (d, *J* = 4.5 Hz, 1H), 8.54 (d, *J* = 4.9 Hz, 1H), 8.16 (d, *J* = 6.5 Hz, 1H), 8.09 (s, 1H), 7.32 (d, *J* = 4.4 Hz, 1H), 7.18 (d, *J* = 5.6 Hz, 1H), 2.41 (s, 3H); MS (ESI): *m*/*z* 338.1 [M + 1]^+^.

##### *N*-(3-Methoxy-2-pyridyl)-3-(4-methyl-2-pyridyl)-1,2,4-thiadiazol-5-amine (**29**) (27% Yield, 99% Purity)

^1^H NMR (400 MHz, DMSO-*d*_6_) δ 11.85 (s, 1H), 8.54–8.52 (m, 1H), 8.10–8.08 (m, 1H), 8.03 (d, *J* = 4.4 Hz, 1H), 7.48–7.47 (m, 1H), 7.35–7.33 (m, 1H), 7.10 (s, 1H), 3.92 (s, 3H), 2.43 (s, 3H); MS (ESI): *m*/*z* 300.1 [M + 1]^+^.

##### *N*-(5-((1-Methylpiperidin-4-yl)oxy)pyridin-2-yl)-3-(pyridin-2-yl)-1,2,4-thiadiazol-5-amine (**36**) (34% Yield, 99% Purity)

^1^H NMR (400 MHz, CD_3_OD): δ 8.68 (m, 1H), 8.32 (d, *J* = 7.6 Hz, 1H), 8.20 (d, *J* = 2.8 Hz, 1H), 7.95–7.99 (m, 1H), 7.48–7.54 (m, 2H), 7.10 (d, *J* = 8.8 Hz, 1H), 4.48 (brs, 1H), 2.84 (brs, 2H), 2.51 (brs, 2H), 2.40 (s, 3H), 2.05–2.10 (m, 2H), 1.85–1.95 (m, 2H). MS (ESI) *m*/*z* 369.2, 370.2 [M + 1, M + 2]^+^.

### General Procedure B for Compounds in Tables 1–4 and 6

#### 3-(5-Cyclopropoxypyridin-2-yl)-*N*-(3-methylpyridin-2-yl)-1,2,4-thiadiazol-5-amine (**11**)

##### 5-Cyclopropoxy-*N*-((3-methylpyridin-2-yl)carbamothioyl)picolinimidamide

To a solution of 2-isothiocyanato-3-methylpyridine (527 mg, 3.51 mmol) and triethylamine (710 mg, 7.02 mmol) in acetone (15 mL) and DCM (15 mL) was added 5-cyclopropoxypicolinimidamide hydrochloride (500 mg, 2.34 mmol, HCl). The mixture was stirred at 25 °C for 20 h. 2-Isothiocyanato-3-methylpyridine (351 mg, 2.34 mmol) was added to the reaction mixture. The mixture was stirred at 25 °C for 4 h and then diluted with water. The aqueous phase was extracted with DCM, and the combined organic layers were washed with brine, dried over anhydrous sodium sulfate, filtered, and concentrated under reduced pressure to give a residue. The residue was purified by silica gel chromatography to give 5-cyclopropoxy-*N*-((3-methylpyridin-2-yl)carbamothioyl)picolinimidamide (400 mg, 0.782 mmol, 33% yield, 64% purity).

##### 3-(5-Cyclopropoxypyridin-2-yl)-*N*-(3-methylpyridin-2-yl)-1,2,4-thiadiazol-5-amine

To a solution of 5-cyclopropoxy-*N*-((3-methylpyridin-2-yl)carbamothioyl)picolinimidamide (400 mg, 0.782 mmol) in ethanol (10 mL) was added a solution of iodine (39.7 mg, 0.156 mmol) in ethanol (0.5 mL) and hydrogen peroxide (177 mg, 1.56 mmol, 30% purity). The mixture was stirred at 25 °C for 6 h. The mixture was quenched with saturated sodium sulfite, and the aqueous phase was extracted with EtOAc. The combined organic layers were washed with brine, dried over anhydrous sodium sulfate, filtered, and concentrated under reduced pressure to give a residue. The residue was triturated with acetonitrile (10 mL). The filter cake and the filtrate showed a similar purity. The filter cake and filtrate were combined to give the crude product. The crude product was purified by prep-HPLC (column: Phenomenex Synergi C18 150 × 25 × 10um; mobile phase: [water (0.225% FA)-ACN]; B%: 40% 70%,11 min) to give 3-(5-cyclopropoxypyridin-2-yl)-*N*-(3-methyl-pyridin-2-yl)-1,2,4-thiadiazol-5-amine (61.95 mg, 0.187 mmol, 24% yield, 98.2% purity). ^1^H NMR (400 MHz, DMSO-*d*_6_) δ 11.79 (s, 1H), 8.43 (d, *J* = 2.7 Hz, 1H), 8.31 (d, *J* = 4.4 Hz, 1H), 8.21 (d, *J* = 8.7 Hz, 1H), 7.70–7.63 (m, 2H), 7.06–7.01 (m, 1H), 4.05–4.02 (m, 1H), 2.40 (s, 3H), 0.89–0.82 (m, 2H), 0.78–0.71 (m, 2H); MS (ESI): *m*/*z* 326.1 [M + 1]^+^.

##### 3-[5-[(2-Cyclopropyl-2-azaspiro[3.3]heptan-6-yl)oxy]-2-pyridyl]-*N*-(3-methyl-2-pyridyl)-1,2,4-thiadiazol-5-mine (**12**) (12% Yield, 100% Purity)

^1^H NMR (400 MHz, DMSO-*d*_6_) δ 11.79 (s, 1H), 8.31–8.30 (dd, *J*_1_ = 5.2, *J*_2_ = 1.2 Hz, 1H), 8.27 (d, *J* = 2.4 Hz, 1H), 8.18 (s, 1H), 8.17–8.14 (d, *J* = 9.6 Hz, 1H), 7.68 (d, *J* = 7.2 Hz, 1H), 7.40–7.38 (dd, *J*_1_ = 1.2, *J*_2_ = 8.4 Hz, 1H), 7.05–7.02 (dd, *J*_1_ = 7.2 z, *J*_2_ = 5.2 Hz, 1H), 4.78–4.74 (m, 2H), 3.28 (s, 2H), 3.20 (s, 2H), 2.69–2.64 (m, 2H), 2.40(s, 3H), 2.19–2.14 (m, 2H), 1.81–1.79 (m, 2H), 0.30–0.27 (m, 2H), 0.19–1.17 (m, 2H); MS (ESI): *m*/*z* 421.2 [M + 1]^+^.

##### 6-(5-((3-Methylpyridin-2-yl)amino)-1,2,4-thiadiazol-3-yl)nicotinonitrile (**13**) (74% Yield, 96% Purity)

^1^H NMR (400 MHz, DMSO-*d*_6_) δ 11.97 (s, 1H), 9.14 (d, *J* = 1.7 Hz, 1H), 8.49–8.44 (m, 1H), 8.40–8.36 (m, 1H), 8.33 (d, *J* = 4.0 Hz, 1H), 7.70 (d, *J* = 7.3 Hz, 1H), 7.06 (dd, *J*_1_ = 7.3, *J*_2_ = 5.1 Hz, 1H), 2.41 (s, 3H); MS (ESI): *m*/*z* 295.1 [M + 1]^+^.

##### 3-[5-[(1-Cyclopropyl-4-piperidyl)oxy]-2-pyridyl]-*N*-(3-methyl-2-pyridyl)-1,2,4-thiadiazol-5-amine (**19**) (17% Yield, 99% Purity)

^1^H NMR (400 MHz, DMSO-*d*_6_) δ 11.77 (s, 1H), 8.36 (d, *J* = 2.8 Hz, 1H), 8.31 (d, *J* = 3.9 Hz, 1H), 8.17 (d, *J* = 8.7 Hz, 1H), 7.67 (d, *J* = 7.1 Hz, 1H), 7.56 (dd, *J*_1_ = 8.8, *J*_2_ = 2.9 Hz, 1H), 7.03 (dd, *J*_1_ = 7.3, *J*_2_ = 5.1 Hz, 1H), 4.59–4.54 (m, 1H), 2.87–2.77 (m, 2H), 2.47–2.37 (m, 5H), 2.01–1.89 (m, 2H), 1.68–1.56 (m, 3H), 0.46–0.38 (m, 2H), 0.34–0.26 (m, 2H); MS (ESI): *m*/*z* 409.2 [M + 1]^+^.

##### 3-(5-((1-Cyclopropyl-3,3-difluoropiperidin-4-yl)oxy)pyridin-2-yl)-*N*-(3-methylpyridin-2-yl)-1,2,4-thiadiazol-5-amine (**20**) (45% Yield, 98% Purity)

^1^H NMR (400 MHz, methanol-*d*_4_) δ 8.42 (d, *J* = 2.8 Hz, 1 H), 8.32–8.28 (m, 2 H), 7.67–7.62 (m, 2H), 7.00 (dd, *J*_1_ = 7.2, *J*_2_ = 5.2 Hz, 1H), 4.85–4.80 (m, 1H), 3.32–3.30 (m, 1H), 2.92–2.89 (m, 2H), 2.73–2.68 (m, 1H), 2.43 (s, 3H), 2.09–2.03 (m, 2H), 1.86–1.84 (m, 1H), 0.57–0.45 (m, 4H); MS (ESI): *m*/*z* 445.2 [M + 1]^+^.

##### 3-(5-Cyclopropoxypyridin-2-yl)-*N*-(3-isopropylpyridin-2-yl)-1,2,4-thiadiazol-5-amine (**23**) (19% Yield, 95% Purity, HCl)

^1^H NMR (400 MHz, DMSO-*d*_6_) δ 11.86 (s, 1H), 8.44 (d, *J =* 2.8 Hz, 1H), 8.33 (d, *J* = 3.6 Hz, 1H), 8.22 (d, *J =* 8.8 Hz, 1H), 7.78 (d, *J =* 7.2 Hz, 1H), 7.66 (dd, *J*_1_*=* 8.8 Hz, *J*_2_ = 2.8 Hz, 1H), 7.10 (d, *J =* 4.8 Hz, 1H), 4.05–4.02 (m, 1H), 3.57–3.35 (m, 1H), 1.22–1.21 (m, 6H), 0.86–0.83 (m, 2H), 0.76–0.72 (m, 2H). MS (ESI): *m*/*z* 354.2 [M + 1]^+^.

##### 3-(5-Isopropoxy-4-(trifluoromethyl)pyridin-2-yl)-*N*-(3-isopropylpyridin-2-yl)-1,2,4-thiadiazol-5-amine (**24**) (19% Yield, 99% Purity)

^1^H NMR (400 MHz, DMSO-*d*_6_) 11.88 (s, 1H), 8.82 (s, 1H), 8.39 (s, 1H), 8.33 (dd, *J*_1_ = 4.9, *J*_2_ = 1.5, 1H), 7.79 (dd, *J*_1_ = 7.6, *J*_2_ = 1.3, 1H), 7.12 (dd, *J*_1_ = 7.5, *J*_2_ = 5.0, 1H), 5.15–5.06 (m, 1H), 3.60–3.50 (m, 1H), 1.37 (d, *J* = 6.0 Hz, 6H), 1.22 (d, *J* = 6.7 Hz, 6H). MS (ESI) *m*/*z* 424.2[M + 1]^+^.

##### 3-(5-Isopropoxy-2-pyridyl)-*N*-(3-isopropyl-2-pyridyl)-1,2,4-thiadiazol-5-amine (**25**) (71% Yield, 97% Purity)

^1^H NMR (400 MHz, DMSO-*d*_6_) δ 8.89 (brs, 1H), 8.40 (d, *J* = 2.8 Hz, 1H), 8.33 (dd, *J*_1_ = 5.0, *J*_2_ = 1.5 Hz, 1H), 8.25 (d, *J* = 8.8 Hz, 1H), 7.61 (dd, *J*_1_ = 7.6, *J*_2_ = 1.1 Hz, 1H), 7.31–7.28 (m, 1H), 7.02 (dd, *J*_1_ = 7.6, *J*_2_ = 5.0 Hz, 1H), 4.72–4.63 (m, 1H), 3.01–2.91 (m, 1H), 1.41–1.34 (m, 12H); MS (ESI): *m*/*z* 356.2 [M + 1]^+^.

##### 3-(5-Isopropoxypyridin-2-yl)-*N*-(3-methoxypyridin-2-yl)-1,2,4-thiadiazol-5-amine (**28**) (31% Yield, 99% Purity)

^1^H NMR (400 MHz, DMSO-*d*_6_) δ 11.78 (brs, 1H), 8.33 (d, *J* = 2.8 Hz, 1H), 8.17 (d, *J* = 8.4 Hz, 1H), 8.02 (dd, *J*_1_ = 1.2 Hz, *J*_2_ = 5.2 Hz, 1H), 7.52–7.47 (m, 2H), 7.08 (dd, *J*_1_ = 5.2 Hz, *J*_2_ = 8 Hz, 1H), 4.80–4.77 (m, 1H), 3.91 (s, 3H), 1.34–1.32 (m, 6H). MS (ESI): *m*/*z* 344.2 [M + 1]^+^.

##### *N*^2^-(3-(5-Isopropoxypyridin-2-yl)-1,2,4-thiadiazol-5-yl)-*N*3,*N*3-dimethylpyridine-2,3-diamine (**31**) (15% Yield, 96% Purity)

^1^H NMR (400 MHz, CD_3_OD) δ 8.35–8.26 (m, 2H), 8.15 (d, *J* = 4.9 Hz, 1H), 7.65–7.54 (m, 2H), 7.06 (dd, *J*_1_ = 7.7, *J*_2_ = 5.0 Hz, 1H), 4.83–4.73 (m, 1H), 2.78 (s, 6H), 1.40 (d, *J =* 6.0 Hz, 6H); MS (ESI): *m*/*z* 357.1 [M + 1]^+^.

##### 3-(4-Isopropylpyridin-2-yl)-*N*-(4-(trifluoromethyl)pyridin-2-yl)-1,2,4-thiadiazol-5-amine (**35**) (17%Yield, 98% Purity) as Off-White Solid

^1^H NMR (400 MHz, DMSO-*d*_6_) δ 12.5 (brs, 1H), 8.74 (d, *J* = 5.6 Hz, 1H), 8.60 (d, *J* = 4.8 Hz, 1H), 8.09 (s, 1H), 7.42–7.38 (m, 3H), 3.03–2.98 (m, 1H), 1.27 (s, 6H). MS (ESI): *m*/*z* 366.1 [M + 1]^+^.

##### 3-(3-Cyclopropoxypyridin-2-yl)-*N*-(5-isopropyl-4-(trifluoromethyl)pyridin-2-yl)-1,2,4-thiadiazol-5-amine (**38**) (13% Yield, 97% Purity)

^1^H NMR (400 MHz, CDCl_3_) δ 12.18 (s, 1H), 8.62 (s, 1H), 8.26 (dd, *J*_1_ = 4.4, *J*_1_ = 1.2 Hz, 1H), 7.67 (dd, *J*_1_ = 8.4, *J*_1_ = 1.2 Hz, 1H), 7.32 (dd, *J*_1_ = 8.4, *J*_1_ = 4.8 Hz, 1H), 6.82 (s, 1H), 3.72–3.58 (m, 1H), 3.27–3.20 (m, 1H), 1.34 (d, *J* = 6.8 Hz, 6H), 0.76–0.65 (m, 4H); MS (ESI) *m*/*z* 422.1 [M + 1]^+^.

##### 3-(5-Isopropoxypyrazin-2-yl)-*N*-(3-methyl-2-pyridyl)-1,2,4-thiadiazol-5-amine (**39**) (30% Yield, 92% Purity) as Yellow Solid

^1^H NMR (400 MHz, DMSO-*d*_6_) δ 8.95 (s, 1H), 8.32 (s, 2H), 7.67 (d, *J* = 7.2 Hz, 1H), 7.04–7.01 (m, 1H), 5.36–5.30 (m, 1H), 2.40 (s, 3H), 1.36 (d, *J* = 6.0 Hz, 1H); MS (ESI): *m*/*z* 329.1 [M + 1]^+^.

##### 3-(5-Isopropoxypyrimidin-2-yl)-*N*-(3-methylpyridin-2-yl)-1,2,4-thiadiazol-5-amine (**40**) (34% Yield, 98% Purity)

^1^H NMR (400 MHz, DMSO-*d*_6_) δ = 11.89 (brs, 1H), 8.65 (s, 2H), 8.32 (dd, *J* = 0.9, 5.0 Hz, 1H), 7.68 (dd, *J* = 0.6, 7.3 Hz, 1H), 7.04 (dd, *J* = 5.0, 7.3 Hz, 1H), 4.98–4.86 (m, 1H), 2.40 (s, 3H), 1.35 (d, *J* = 6.0 Hz, 6H). MS (ESI): *m*/*z* 329.3 [M + 1]^+^.

##### 3-(5-Isopropoxy-2-pyridyl)-*N*-(3-methylpyrazin-2-yl)-1,2,4-thiadiazol-5-amine (**41**) (18% Yield, 96% Purity)

^1^H NMR (400 MHz, DMSO-*d*_6_) δ 12.22 (brs, 1H), 8.34 (d, *J* = 2.4 Hz, 2H), 8.21–8.15 (m, 2H), 7.52 (dd, *J*_1_ = 8.8, *J*_2_ = 2.9 Hz, 1H), 4.82–4.75 (m, 1H), 2.66 (s, 3H), 1.33 (d, *J* = 6.0 Hz, 6H), MS (ESI): *m*/*z* 329.1 [M + 1]^+^.

##### 3-(5-Isopropoxypyridin-2-yl)-*N*-methyl-*N*-(3-methylpyridin-2-yl)-1,2,4-thiadiazol-5-amine (**42**) (13% Yield, 99% Purity)

^1^H NMR (400 MHz, DMSO-*d*_6_) δ 8.45–8.26 (m, 2H), 8.13 (d, *J* = 8.8 Hz, 1H), 7.83 (d, *J* = 7.2 Hz, 1H), 7.49 (dd, *J*_1_ = 8.8, *J*_2_ = 2.8 Hz, 1H), 7.27 (dd, *J*_1_ = 7.6, *J*_2_ = 4.8 Hz, 1H), 4.82–4.73 (m, 1H), 3.81 (s, 3H), 2.47 (s, 3H), 1.32 (d, *J* = 6.0 Hz, 6H); MS (ESI) *m*/*z* 342.2 [M + 1]^+^.

##### 3-(5-Isopropoxypyridin-2-yl)-*N*-isopropyl-*N*-(3-methylpyridin-2-yl)-1,2,4-thiadiazol-5-amine (**43**) (54% Yield, 99% Purity Formic Acid)

^1^H NMR (400 MHz, DMSO-*d*_6_) δ 8.47 (d, *J* = 3.2 Hz, 1H), 8.31 (d, *J* = 2.8 Hz, 1H), 8.17 (s, 1H), 8.04 ( d, *J* = 8.8 Hz, 1H), 7.48–7.45 (m, 2H), 4.79–4.73 (m, 1H), 4.57–4.52 (m, 1H), 2.27 (s, 3H), 1.37 (s, 3H), 1.35 (s, 3H), 1.32 (s, 3H), 1.30 (s, 3H); MS (ESI): *m*/*z* 370.1 [M + 1]^+^.

##### 3-(5-Isopropoxypyridin-2-yl)-*N*-(3-isopropylpyridin-2-yl)-*N*-methyl-1,2,4-thiadiazol-5-amine (**44**) (55% Yield, 95% Purity)

^1^H NMR (400 MHz, CD_3_OD) 8.42 (dd, *J*_1_ = 4.8, *J*_2_ = 1.7 Hz, 1H), 8.27 (d, *J* = 2.6 Hz, 1H), 8.19 (d, *J* = 8.7 Hz, 1H), 8.06 (dd, *J*_1_ = 7.9, *J*_2_ = 1.7 Hz, 1H), 7.56–7.44 (m, 2H), 4.80–4.74 (m, 1H), 3.70 (s, 3H), 3.24–3.14 (m, 1H), 1.39 (d, *J* = 6.1 Hz, 6H), 1.30 (d, *J* = 6.8 Hz, 6H); MS (ESI) *m*/*z* 370.2 [M + 1]^+^.

##### *N*-(2-((3-(5-Isopropoxypyridin-2-yl)-1,2,4-thiadiazol-5-yl)amino)pyridin-3-yl)-*N*-methylacetamide (**47**) (70% Yield, 98% Purity)

^1^H NMR (400 MHz, CDCl_3_) 8.50 (d, *J* = 3.7 Hz, 1H), 8.39 (d, *J* = 2.4 Hz, 1H), 8.23 (d, *J* = 8.7 Hz, 1H), 7.58 (dd, *J*_1_ = 7.6, *J*_2_ = 1.2, 1H), 7.29 (dd, *J*_1_ = 8.8, *J*_2_ = 2.9, 1H), 7.13 (dd, *J*_1_ = 7.6, *J*_2_ = 5.0, 1H), 4.71–4.64 (m, 1H), 3.39 (s, 0.3H), 3.26 (s, 2.7H), 2.39 (s, 0.3H), 1.87 (s, 2.7H), 1.40 (d, *J* = 6.0 Hz, 6H); MS (ESI) *m*/*z* 385.2 [M + 1]^+^.

##### *tert*-Butyl Methyl(6-(5-((3-methylpyridin-2-yl)amino)-1,2,4-thiadiazol-3-yl) pyridin-3-yl)carbamate (**45**) (21% Yield, 99% Purity)

^1^H NMR (400 MHz, DMSO-*d*_6_) 11.84 (s, 1H), 8.69 (d, *J* = 2.1 Hz, 1H), 8.33 (d, *J* = 4.3 Hz, 1H), 8.28 (d, *J* = 8.3 Hz, 1H), 7.96 (d, *J* = 7.6 Hz, 1H), 7.68 (d, *J* = 7.2 Hz, 1H), 7.04 (dd, *J*_1_ = 7.1, *J*_2_ = 5.1 Hz, 1H), 3.29–3.22 (m, 3H), 2.41 (s, 3H), 1.94 (s, 3H); MS (ESI): *m*/*z* 341.2 [M + 1]^+^.

##### *N*-(2-((3-(5-Isopropoxy-4-(trifluoromethyl)pyridin-2-yl)-1,2,4-thiadiazol-5-yl)amino)pyridine-3-yl)-*N*-methylcyclopropanecarboxamide (**49**) (45% Yield, 99% Purity)

^1^H NMR (400 MHz, DMSO-*d*_6_) δ 12.85–12.04 (m, 1H), 8.81 (s, 1H), 8.50 (dd, *J*_1_ = 4.9, *J*_2_ = 1.3 Hz, 1H), 8.37 (s, 1H), 7.90 (d, *J* = 6.6 Hz, 1H), 7.26–7.12 (m, 1H), 5.16–5.07 (m, 1H), 3.45 (s, 0.4H), 3.12 (s, 2.5H), 1.37 (d, *J* = 6.0 Hz, 6H), 1.18–1.08 (m, 1H), 0.94–0.51 (m, 4H); MS (ESI): *m*/*z* 479.1 [M + 1]^+^.

##### *N*-(2-((3-(5-Isopropoxypyridin-2-yl)-1,2,4-thiadiazol-5-yl)amino)pyridin-3-yl)-*N*-methylcyclopropanecarboxamide (**50**) (98% Yield, 99% Purity)

^1^H NMR (400 MHz, CDCl_3_) δ ppm 8.45–8.56 (m, 1 H), 8.35–8.42 (m, 1 H), 8.18–8.29 (m, 1 H), 7.59–7.69 (m, 1 H), 7.27–7.33 (m, 1 H), 7.06–7.19 (m, 1 H), 4.57–4.79 (m, 1 H), 3.20–3.32 (m, 3 H), 1.36–1.44 (m, 6 H),1.08–1.22 (m, 2 H), 0.97–1.05 (m, 1 H), 0.60–0.72 (m, 2 H): MS(ESI): *m*/*z* 411.2 [M + 1]^+^.

##### 1-(2-((3-(5-Isopropoxy-4-(trifluoromethyl)pyridin-2-yl)-1,2,4-thiadiazol-5-yl)amino)pyridin-3-yl)pyrrolidin-2-one (**52**) (32% Yield, 98% Purity)

^1^H NMR (400 MHz, DMSO-*d*_6_) δ 12.08 (s, 1H), 8.82 (s, 1H), 8.45 (dd, *J*_1_ = 4.8 Hz, *J*_2_ = 1.3 Hz, 1H), 8.38 (s, 1H), 7.85–7.83 (m, 1H), 7.21–7.18 (m, 1H), 5.15–5.08 (m, 1H), 3.74 (t, *J* = 7.0 Hz, 2H), 2.48–2.42 (m, 2H), 2.25–2.20 (m, 2H), 1.37 (s, 3H), 1.36 (s, 3H); MS (ESI): *m*/*z* 465.2 [M + 1]^+^.

##### *N*-(2-((3-(5-Isopropoxypyridin-2-yl)-1,2,4-thiadiazol-5-yl)amino)-5-(trifluoromethyl)pyridin-3-yl)-*N*-methylacetamide (**54**) (25% Yield, 99% Purity)

^1^H NMR (400 MHz, DMSO-*d*_6_) δ 13.00–12.51 (m, 1H), 8.91–8.83 (m, 1H), 8.38–8.32 (m, 1.5H), 8.18–8.16 (m, 1.5H), 7.52 (d, *J*_1_ = 8.8, *J*_2_ = 3.2 Hz, 1H), 4.81–4.76 (m, 1H), 3.31 (s, 1.9 H), 3.10 (s, 1.3H), 2.22 (s, 1.6 H), 1.73 (s, 1.4H), 1.32 (d, *J* = 6.0 Hz, 6H); MS (ESI): *m*/*z* 453.3 [M + 1]^+^.

##### *N*-(2-((3-(4-Isopropoxypyridin-2-yl)-1,2,4-thiadiazol-5-yl)amino)-5-(trifluoromethyl)pyridin-3-yl)-*N*-methylacetamide (**55**) (40% Yield, 95% Purity)

^1^H NMR (400 MHz, DMSO-*d*_6_) δ 13.13–12.68 (m, 1H), 8.97–8.88 (m, 1H), 8.59 (d, *J* = 6.3 Hz, 1H), 8.45–8.24 (m, 1H), 7.84 (dd, *J*_1_ = 8.7, *J*_2_ = 2.6 Hz, 1H), 7.34–7.32 (m, 1H), 5.02–4.94 (m, 1H), 3.32 (s, 1.6H), 3.11 (s, 1.4H), 2.23 (s, 1.6H), 1.74 (s, 1.4H), 1.38 (d, *J* = 6.0 Hz, 6H); MS (ESI): *m*/*z* 453.3 [M + 1]^+^.

##### *N*-(2-((3-(4-Isopropylpyridin-2-yl)-1,2,4-thiadiazol-5-yl)amino)-5-(trifluoromethyl)pyridin-3-yl)-*N*-ethylacetamide (**56**) (30% Yield, 96% Purity)

^1^H NMR (400 MHz, DMSO-*d*_6_) 13.14–12.48 (m, 1H), 8.95–8.83 (m, 1H), 8.58 (d, *J* = 5.0 Hz, 1H), 8.39 (s, 0.5H), 8.19 (d, *J* = 2.1 Hz, 0.5H), 8.13–8.12 (m, 1H), 7.39 (dd, *J* = 1.6, 5.0 Hz, 1H), 3.31 (s, 1.6H), 3.10 (s, 1.4H), 3.04–2.97 (m, 1H), 2.23 (s, 1.6H), 1.73 (s, 1.4H), 1.26 (d, *J* = 7.0 Hz, 6H), MS (ESI): *m*/*z* 437.3 [M + 1]^+^.

##### 2-[[3-(3-Isopropoxy-2-pyridyl)-1,2,4-thiadiazol-5-yl]amino]-*N*,*N*-dimethyl-5-(trifluoromethyl)pyridine-3-carboxamide (**58**) (>100% Yield, 94% Purity)

^1^H NMR (400 MHz, DMSO-*d*_6_) δ = 12.65–12.29 (m, 1H), 8.94 (d, *J* = 1.1 Hz, 1H), 8.25–8.17 (m, 2H), 7.65 (d, *J* = 8.6 Hz, 1H), 7.47 (dd, *J*_1_ = 4.5, *J*_2_ = 8.4 Hz, 1H), 4.66–4.56 (m, 1H), 3.01 (s, 3H), 2.87 (s, 3H), 1.21 (d, *J* = 6.1 Hz, 6H). LCMS (ESI): *m*/*z* 453.1 [M + 1]^+^.

##### 2-((3-(5-Isopropoxypyridin-2-yl)-1,2,4-thiadiazol-5-yl)amino)nicotinamide (**59**) (55% Yield, 95% Purity)

^1^H NMR (400 MHz, DMSO-*d*_6_) 12.92 (s, 1H), 8.64 (dd, *J*_1_ = 4.9, *J*_2_ = 1.3 Hz, 1H), 8.60 (s, 1H), 8.45 (dd, *J*_1_ = 7.8, *J*_2_ = 1.5 Hz, 1H), 8.33 (d, *J* = 2.7 Hz, 1H), 8.17 (d, *J* = 8.8 Hz, 1H), 8.11 (s, 1H), 7.50 (dd, *J*_1_ = 8.8, *J*_2_ = 2.9 Hz, 1H), 7.25 (dd, *J*_1_ = 7.8, *J*_2_ = 5.0 Hz, 1H), 4.82–4.76 (m, 1H), 1.33 (d, *J* = 6.0 Hz, 6H); MS (ESI): *m*/*z* 357.3 [M + 1]^+^.

##### 2-((3-(5-Isopropoxypyridin-2-yl)-1,2,4-thiadiazol-5-yl)amino)-*N*-methylnicotinamide (**60**) (69% Yield, 96% Purity)

^1^H NMR (400 MHz, DMSO-*d*_6_) 12.75 (s, 1H), 9.06 (s, 1H), 8.63 (d, *J* = 3.5 Hz, 1H), 8.41–8.28 (m, 2H), 8.18 (d, *J* = 8.8 Hz, 1H), 7.58–7.47 (m, 1H), 7.26–7.24 (m, 1H), 4.83–4.76 (m, 1H), 2.87 (d, *J* = 4.4 Hz, 3H), 1.33 (d, *J* = 6.1 Hz, 6H); MS (ESI): *m*/*z* 371.1 [M + 1]^+^.

##### 2-((3-(5-Isopropoxypyridin-2-yl)-1,2,4-thiadiazol-5-yl)amino)-*N*,*N*-dimethylnicotinamide (**61**) (77% Yield, 99% Purity)

^1^H NMR (400 MHz, DMSO-*d*_6_) 11.91 (s, 1H), 8.52 (dd, *J*_1_ = 5.0 Hz, *J*_2_ = 1.6 Hz, 1H), 8.33 (d, *J* = 2.8 Hz, 1H), 8.16 (d, *J* = 8.7 Hz, 1H), 7.80 (dd, *J*_1_ = 7.5 Hz, *J*_2_ = 1.7 Hz, 1H), 7.52 (dd, *J*_1_ = 8.7 Hz, *J*_2_ = 2.9 Hz, 1H), 7.17 (dd, *J*_1_ = 7.4 Hz, *J*_2_ = 5.1 Hz, 1H), 4.83–4.73 (m, 1H), 3.02 (s, 3H), 2.88 ( s, 3H), 1.32 (d, *J* = 6.0 Hz, 6H); MS (ESI): *m*/*z* 385.2 [M + 1]^+^.

### Procedures for Compounds in Table 1 Containing Cores B, D, E, and F

#### 3-Methyl-*N*-{5-[5-(oxan-4-yloxy)pyridin-2-yl]-1,3,4-thiadiazol-2-yl}pyridin-2-amine (**4**)

##### 5-(Tetrahydro-pyran-4-yloxy)-pyridine-2-carboxylic Acid Hydrazide

To the stirred solution of 5-(tetrahydro-pyran-4-yloxy)-pyridine-2-carboxylic acid methyl ester (600 mg, 2.53 mmol) in methanol (10 mL), hydrazine hydrate (506 mg, 10.11 mmol) was added and the resulting mixture was heated at reflux for 2 h. After completion, the reaction mixture was concentrated under reduced pressure to provide 5-(tetrahydro-pyran-4-yloxy)-pyridine-2-carboxylic acid hydrazide (565 mg, 94% yield). MS (ESI): *m*/*z* 238.2 [M + 1]^+^.

##### *N*-{[(3-Methylpyridin-2-yl)carbamothioyl]amino}-5-(oxan-4-yloxy)pyridine-2-carboxamide

To a stirred solution of 5-(tetrahydro-pyran-4-yloxy)-pyridine-2-carboxylic acid hydrazide (500 mg, 2.17 mmol) in DCM (10 mL), 2-isothiocyanato-3-methyl-pyridine (379.8 mg, 2.53 mmol) was added and the reaction mixture was stirred at room temperature for 19 h. The reaction mixture was concentrated under reduced pressure to provide *N*-{[(3-methylpyridin-2-yl)carbamothioyl]amino}-5-(oxan-4-yloxy)pyridine-2-carboxamide (610 mg, 75% yield), which was used in the next step without further purification.

##### 3-Methyl-*N*-{5-[5-(oxan-4-yloxy)pyridin-2-yl]-1,3,4-thiadiazol-2-yl}pyridin-2-amine

To a stirred solution of *N*-{[(3-methylpyridin-2-yl)carbamothioyl]amino}-5-(oxan-4-yloxy)pyridine-2-carboxamide (200 mg, 0.52 mmol) in toluene (5 mL), *p*-toluene sulfonic acid (98 mg, 0.57 mmol) was added at 25 °C and the resulting mixture was heated at 100 °C for 16 h. The reaction mixture was quenched with water and extracted with 10% IPA-DCM. Combined organic layers were dried over anhydrous sodium sulfate, filtered, and evaporated under reduced pressure. The residue was purified by prep-HPLC to afford 3-methyl-*N*-{5-[5-(oxan-4-yloxy)pyridin-2-yl]-1,3,4-thiadiazol-2-yl}pyridin-2-amine (35 mg, 18% yield, 99% purity). ^1^H NMR (400 MHz, DMSO-*d*_6_): δ 10.86 (brs, 1H), 8.35 (d, *J* = 2.4 Hz, 1H), 8.21 (d, *J* = 4 Hz, 1H), 8.09 (d, *J* = 8.2 Hz, 1H), 7.62–7.57 (m, 2H), 6.69–6.93 (m, 1H), 4.75–4.71 (m, 1H), 3.86–3.83 (m, 2H), 3.50–3.45 (m, 2H), 2.34 (s, 3H), 2.01–1.98 (m, 2H), 1.65–1.57 (m, 2H); MS (ESI): *m*/*z* 370.2 [M + 1]^+^.

#### *N*-(3-Methylpyridin-2-yl)-3-(5-((tetrahydro-2*H*-pyran-4-yl)oxy)pyridin-2-yl)-1,2,4-oxadiazol-5-amine (**6**)

##### *N*-Hydroxy-5-((tetrahydro-2*H*-pyran-4-yl)oxy)picolinimidamide

To a solution of 5-((tetrahydro-2*H*-pyran-4-yl)oxy)picolinonitrile (1.50 g, 7.34 mmol) and triethylamine (2.23 g, 22.0 mmol) in ethanol (30.0 mL) was added hydroxylamine (510 mg, 7.34 mmol, HCl). The mixture was stirred at 80 °C for 16 h and then concentrated. The residue was diluted with 150 mL of ethyl acetate and 50 mL of brine. The organic phase was separated and dried over anhydrous sodium sulfate, filtered, and concentrated under vacuum to give *N*-hydroxy-5-((tetrahydro-2*H*-pyran-4-yl)oxy)picolinimidamide (1.60 g, crude).

##### 3-(5-((Tetrahydro-2*H*-pyran-4-yl)oxy)pyridin-2-yl)-5-(trichloromethyl)-1,2,4-oxadiazole

To a solution of *N*-hydroxy-5-((tetrahydro-2*H*-pyran-4-yl)oxy)picolinimidamide (1.60 g, 6.74 mmol) in toluene (30 mL) was added trichloroacetic anhydride (4.16 g, 13.5 mmol). The mixture was stirred at 110 °C for 24 h. The mixture was concentrated and diluted with 100 mL of EtOAc. The organic phase was washed with saturated sodium bicarbonate and brine, dried over anhydrous sodium sulfate, filtered, and concentrated under vacuum. The residue was purified by column chromatography to give 3-(5-((tetrahydro-2*H*-pyran-4-yl)oxy)pyridin-2-yl)-5-(trichloromethyl)-1,2,4-oxadiazole (550 mg, 1.24 mmol, 18% yield, 82% purity). ^1^H NMR (400 MHz, CDCl_3_) δ 8.50 (d, *J* = 2.8 Hz, 1H), 8.11 (d, *J* = 8.8 Hz, 1H), 7.34 (dd, *J*_1_*=* 8.8, *J*_2_ = 2.8 Hz, 1H), 4.70–4.61(m, 1H), 4.05–3.97 (m, 2H), 3.66–3.60 (m, 2H), 2.13–2.02 (m, 2H), 1.90–1.81 (m, 2H).

##### (3-Methyl-pyridin-2-yl)-{5-[5-(tetrahydro-pyran-4-yloxy)-pyridin-2-yl]-[1,3,4]oxadiazol-2-yl}-amine (**7-**)

To a stirred solution of *N*-{[(3-methylpyridin-2-yl)carbamothioyl]amino}-5-(oxan-4-yloxy)pyridine-2-carboxamide (400 mg, 1.03 mmol) in DCM (12 mL) was added triethylamine (0.3 mL, 2.06 mmol) and 2-iodoxybenzoic acid (IBX) (376.2 mg, 1.34 mmol) at 0 °C and stirred for 1 h under cooling conditions. Upon completion, the reaction mixture was diluted with DCM and washed with saturated NaHCO_3_ solution, brine, dried over anhydrous sodium sulfate, filtered, and evaporated under reduced pressure. The residue was purified by repeated prep-TLC to provide the title compound (25 mg, 6.84% yield, 95.8% purity). ^1^H NMR (400 MHz, DMSO-*d*_6_): δ 13.26 (brs, 1H), 8.43 (s, 1H), 8.00 (d, *J* = 8.8 Hz, 1H), 7.96 (brs, 1H), 7.64–7.63 (m, 2H), 6.69 (m, 1H), 4.81–4.77 (m, 1H), 3.90–3.85 (m, 2H), 3.53–3.48 (m, 2H), 2.24 (s, 3H), 2.03–2.01 (m, 2H), 1.67–1.59 (m, 2H); MS (ESI): *m*/*z* 353.9 [M + 1]^+^.

#### 3-Methyl-*N*-{5-[5-(oxan-4-yloxy)pyridin-2-yl]-4*H*-1,2,4-triazol-3-yl}pyridin-2-amine (**8**)

##### 1-Benzoyl-3-(3-methylpyridin-2-yl)thiourea

To a stirred solution of 3-methylpyridin-2-amine (3.0 g, 27.14 mmol) in acetone (50 mL) was added benzoyl isothiocyanate (4.7 g, 28.85 mmol) at 0 °C and stirred for 1 h. The reaction mixture was warmed to room temperature and stirred for 2 h. The resultant solid was filtered, washed with cold water and Et_2_O, and dried under vacuum to provide 1-benzoyl-3-(3-methylpyridin-2-yl)thiourea (3.2 g, 42% yield). MS (ESI): *m*/*z* 272.2 [M + 1]^+^.

##### (3-Methylpyridin-2-yl)thiourea

A 10% aqueous NaOH solution (10 mL) was added to 1-benzoyl-3-(3-methylpyridin-2-yl)thiourea (3.2 g, 11.79 mmol) and heated to 80 °C for 40 min. The reaction mixture was cooled to 0 °C and acidified with 1(N) HCl to pH ∼4. The mixture was then basified with saturated solution of KHCO_3_ (pH ∼8). The resulting solid was filtered, washed with cold water and Et_2_O, and dried under vacuum to get (3-methylpyridin-2-yl)thiourea (1.8 g, 91% yield). MS (ESI): *m*/*z* 168.2 [M + 1]^+^.

##### *N*-(3-Methylpyridin-2-yl)(methylsulfanyl)methanimidamide

To a stirred solution of (3-methylpyridin-2-yl)thiourea (1.2 g, 7.17 mmol) in acetone (30 mL) was added MeI (4.5 mL, 71.76 mmol) at room temperature under an argon atmosphere. The resulting reaction mixture was stirred at RT for 18 h. The resulting solid was filtered, washed with acetone, and dried to afford the title compound (1.2 g, 92% yield). MS (ESI): *m*/*z* 182.3 [M + 1]^+^.

##### 3-Methyl-*N*-{5-[5-(oxan-4-yloxy)pyridin-2-yl]-4*H*-1,2,4-triazol-3-yl}pyridin-2-amine

To a stirred solution in a sealed tube was added 5-(oxan-4-yloxy)pyridine-2-carbohydrazide (450 mg, 1.89 mmol) in anhydrous pyridine (9 mL) *N*-(3-methylpyridin-2-yl)(methylsulfanyl)methanimidamide (360 mg, 1.99 mmol) and heated to 140 °C for 5 h. The reaction mixture was concentrated under reduced pressure, and the residue was purified by column chromatography followed by trituration with 5% EtOAc ether to provide 3-methyl-*N*-{5-[5-(oxan-4-yloxy)pyridin-2-yl]-4*H*-1,2,4-triazol-3-yl}pyridin-2-amine (40 mg, 6% yield, 99% purity). ^1^H NMR (400 MHz, DMSO-*d*_6_): δ 13.37 (brs, 1H), 9.91 (s, 1H), 8.36 (s, 1H), 8.15 (s, 1H), 7.95 (d, *J* = 8.2 Hz, 1H), 7.54 (d, *J* = 6.8 Hz, 2H), 6.89 (m, 1H), 4.73–4.71 (m, 1H), 3.90–3.85 (m, 2H), 3.53–3.47 (m, 2H), 2.30 (s, 3H), 2.03–2.00 (m, 2H), 1.67–1.58 (m, 2H); MS (ESI): *m*/*z* 353.2 [M + 1]^+^.

## Supporting Information Available

Full experimental details for preparation of intermediates and compounds **1**, **5**, **14**, **15**, **17**, **22**, **27**, **30**, **32**, **34**, **37**, **43**, **46**, **48**, **51**, and **57** and NMR spectra and LCMS traces for key compounds; additional method details for log *D* and kinetic solubility measurement, metabolic stability, and permeability assays; molecular formula strings (SMILES); *O. gutturosa* motility and MTT viability inhibition EC_50_, hERG IC_50_, and *O. volvulus* L5 % inhibition; and MTT/formazan viability data and additional figures illustrating motility EC_50_ curves and THLE-2 cytotoxicity data (PDF)
Excel file featuring the compounds used in this work (CSV)

## ABBREVIATIONS

ADME: absorption, distribution, metabolism, and excretion
ACN: acetonitrile
AT: aminothiazole
AUC: area under the plasma drug concentration time curve
BBB: blood brain barrier
CL: total clearance of drug from plasma
*C*_max_: maximum plasma drug concentration during a dosing interval
DCM: dichloromethane
DMF: dimethylformamide
DMSO: dimethylsulfoxide
EtOAc: ethyl acetate
FA: formic acid
HOAc: glacial acetic acid
IPA: isopropyl alcohol
L5: young adult worms
dpi: days post infection
MDR1-MDCK: multidrug resistance-1 transfected Madin Darby canine kidney cell line
MeOH: methanol
MTT: 3-(4,5-dimethylthiazol-2-yl)-2,5-diphenyltetrazolium bromide
*P*_app_: apparent permeability
PK: pharmacokinetic
PPB: plasma protein binding
*rac*: racemic
S9: supernatant from 9000 g × 20 min fractionation of liver preparation
THF: tetrahyrofuran
Vss: volume of distribution
