# Supplementary material for: Discovery of Substituted Di(pyridin-2-yl)-1,2,4-thiadiazol-5-amines as Novel Macrofilaricidal Compounds for the Treatment of Human Filarial Infections
Source: J Med Chem. 2022 Aug 16;65(16):11388–403. doi: 10.1021/acs.jmedchem.2c00960 (PMC9421654; doi:10.1021/acs.jmedchem.2c00960)
Supplement: Supplementary file 1 — jm2c00960_si_001.pdf [file jm2c00960_si_001.pdf]

Supporting information: Discovery of Substituted Di(pyridin-2-yl)-1,2,4-thiadiazol-5-amines as Novel Macrofilaricidal Compounds for the Treatment of Human Filarial Infections.

Natalie Hawryluk*[1], Dale Robinson[1], Yixing Shen[1], Graham Kyne[2], Matthew Bedore[2], Sanjay Menon[2], Stacie Canan[1], Thomas von Geldern[3], Simon Townson[4], Suzanne Gokool[4], Alexandra Ehrens[5,6], Marianne Koschel[5], Nathaly Lhermitte-Vallarino[7], Coralie Martin[7], Achim Hoerauf[5,6], Geraldine Hernandez[1], Deepak Dalvie[1], Sabine Specht[5,8], Marc Peter Hübner[5,6], Ivan Scandale[8]

[1]Bristol Myers Squibb, San Diego, CA, USA 92121

[2]Zoetis, Kalamazoo, MI 49001

[3]Embedded Consulting, Richmond, IL 60071

[4]Northwick Park Institute for Medical Research, London, UK, HA1 3UJ

[5]Institute for Medical Microbiology, Immunology & Parasitology, University Hospital Bonn, 53127 Bonn, Germany

[6]German Center for Infection Research (DZIF), partner site Bonn-Cologne, 53127 Bonn, Germany

[7]Unité Molécules de Communication et Adaptation des Microorganismes (MCAM, UMR 7245), Muséum national d'Histoire naturelle, Paris, France 75005

[8]Drugs for Neglected Diseases initiative, Geneva, Switzerland 1204

* nataliehawryluk@gmail.com

Supporting Information Table of Contents

Table S1, Compound **31** cross species PK data	page S2
Table S2, THLE-2 Cytotoxicity data	page S3
Data S1, *O. gutturosa* motility EC_{50} curves	page S4-S9
Data S2, Compound **31,** *L. sigmodontis*-infected jird *in vivo* model	page S9
Data S3. *O. gutturosa* motility and MTT viability inhibition EC_{50}, hERG IC_{50}, *O. volvulus* L5 % inhibition. MTT/formazan viability data	page S10-S11
Methods Procedures S1	page S12-S13
Experimental Procedures S1	page S13-S32
NMR spectra Data S4	page S32-S46
LCMS traces Data S5	pages S47-S67
References	page S68

Table S1. Compound **31**cross species PK data.

**Pharmacokinetic Properties Compound 31**		Rat p.o. 10 mg/kg i.v. 2 mg/kg (n = 4)	Dog p.o. 3 mg/kg i.v. 2 mg/kg (n = 3)
		Mean (±SD) Value	Mean (±SD) Value
iv	CL (mL/min/kg)	8.22±2.72	18.8 ± 2.7
	Vss (L/kg)	2.07±0.92	3.08± 0.03
po	Cmax (μM)	6.28±0.90	1.41±0.53
	Tmax (hr)	2.0±0.0	1.2±0.8
	$AUC_{(0\text{-}inf)}$ (μM·hr)	38.1±5.1	4.02±1.03
	F (%)	63±20	55±21

IV formulation vehicle for rat: 15% DMA / 50%PEG / 35% D5W; for dog: 5% DMA / 10%EtOH / 40% PEG400 / 45% D5W

PO formulation vehicle for mouse and rat: a suspension in 0.5% CMC / 0.25% Tween80, as a suspension; for dog: a suspension in 0.5% methyl cellulose / 0.25% Tween80

THLE-2 Cytotoxicity Assay: THLE-2 cells (ATCC, catalog #CRL-2706) were plated in LHC-8 Media (Invitrogen, catalog #12679015) supplemented with fetal bovine serum (GE, catalog #SH30088.03), 70 ng/mL phosphoethanolamine (Sigma, catalog #P0503), 5 ng/mL EGF (Sigma, catalog #E9644), 1X penicillin/streptomycin (Gibco, catalog #15140148), and 1X glutamine (Invitrogen, catalog #25030024) in 384-well clear-bottom, black walled plates at a density of 3750 cells/well (15µL/well). After plating, cells were incubated overnight at 37°C in an incubator with 5% CO_2 and 90% humidity. Compounds were first serially diluted in dimethyl sulfoxide then diluted in LHC-8 media growth media to 2X concentration. Cells were dosed with 15µL of each dose for final concentrations between 0.01 – 300 µM. Cells were incubated with compounds for 24 or 72 hours. Cytotoxicity was determined with Cell Titer Glo Luminescent ATP Assay (Promega, catalog #G7573) according to the manufacturer's instructions. Tamoxifen (Sigma, catalog #BC9353476) was used as a positive control. ATP levels at each dose were normalized to that of cells dosed with dimethyl sulfoxide vehicle alone and IC_{50} values were calculated from non-linear regression fits of dose-response curves plotted in Graph Pad Prism.

Table S2. THLE data for select compounds

Compound	THLE IC_{50}* (µM)	Compound	THLE IC_{50}* (µM)
1	72.44*	49	18.3
2	>300*	48	8.3
10	38.2*	61	32.5
26	65.6*	54	6.1
11	>200*		
19	120.6*		
31	36.0*		
32	0.8		
28	7.7		
12	5.2		
35	2.3		
30	17.5		
25	3.2		
50	7.4		

*indicates 24hr timepoint

Data S1. *Onchocerca gutturosa* adult male worms EC_{50} Curves

Plotted using Graph Pad Prism 7.03, least square original fit, sigmoidal, 4PL
The measurement of mean worm motility every 24 h, terminating at 120 h, serial 1 in 4 drug dilutions to find activity endpoint and produce EC_{50} values (geomean, n=2) for motility reduction. (4 worms per drug concentration)

1

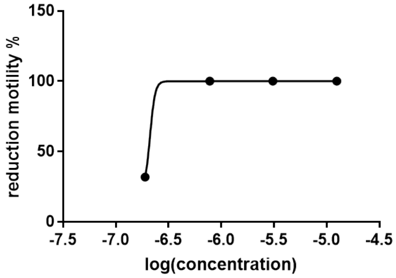

2

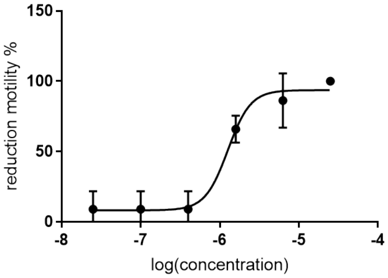

3

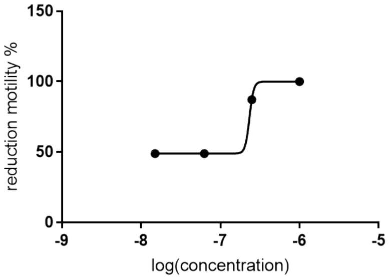

4

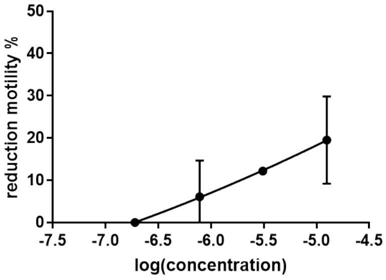

6

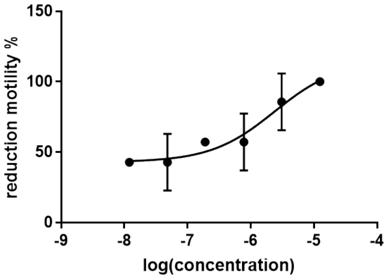

7

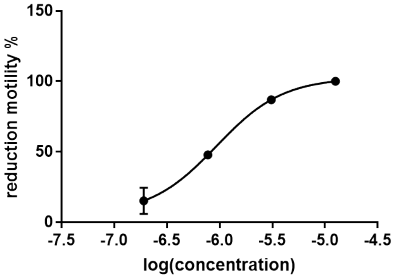

8

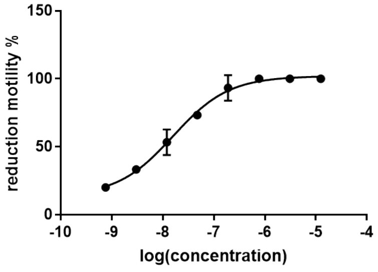

9

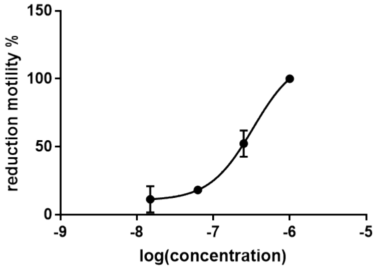

10

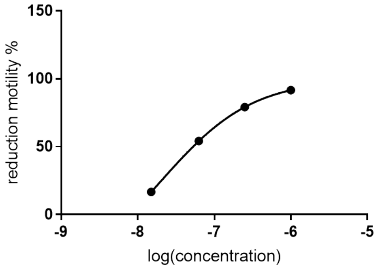

11

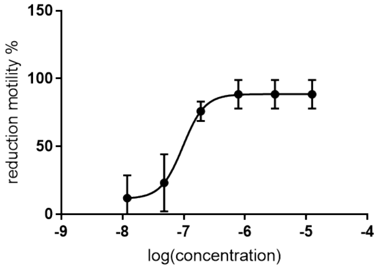

12

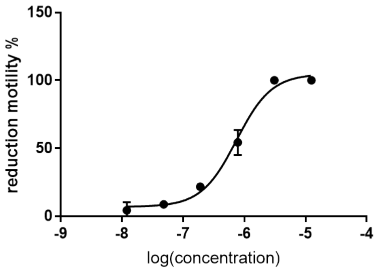

13

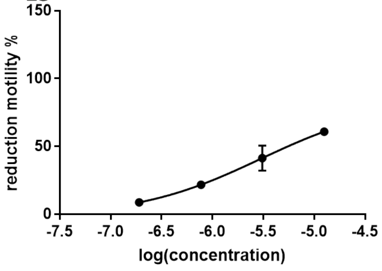

14

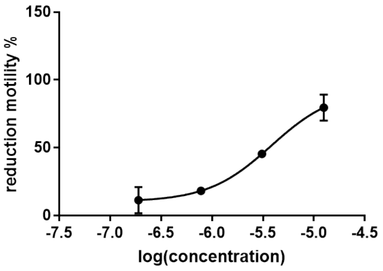

15

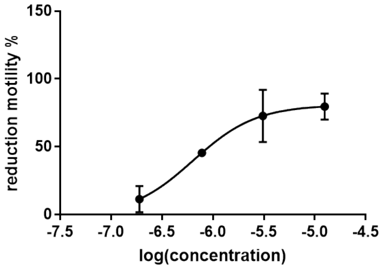

16

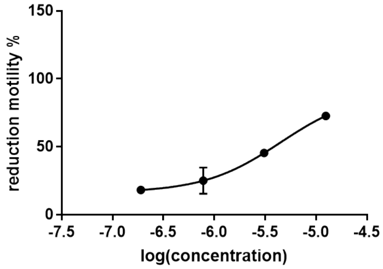

17

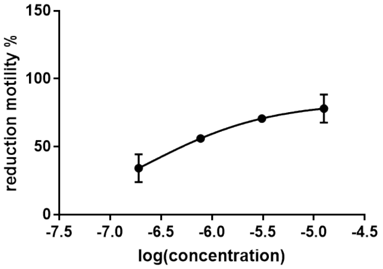

18

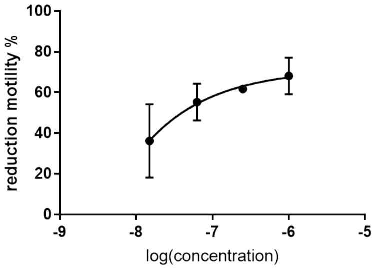

19

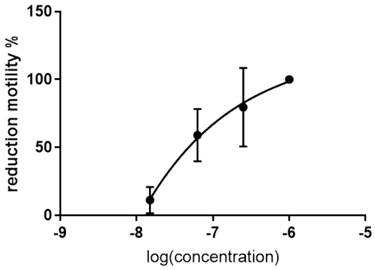

20

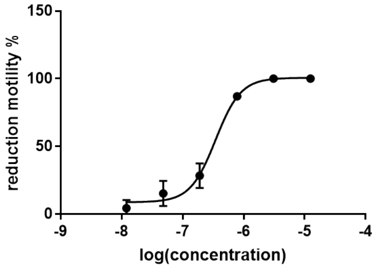

21

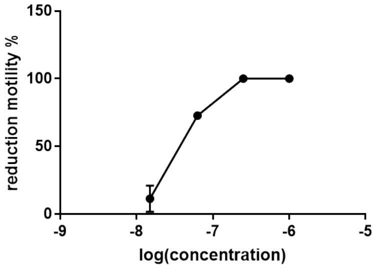

22

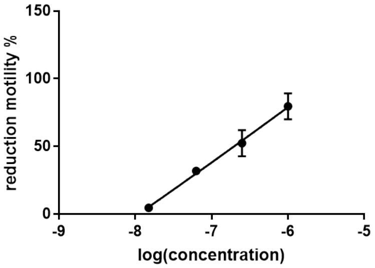

23

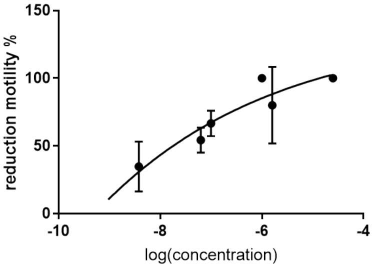

24

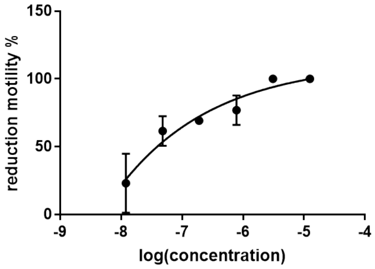

25

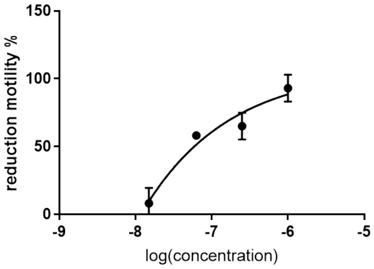

26

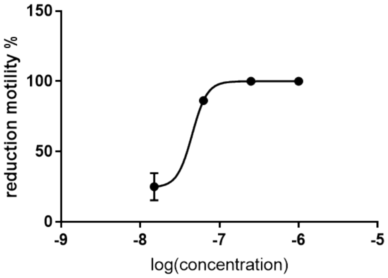

27

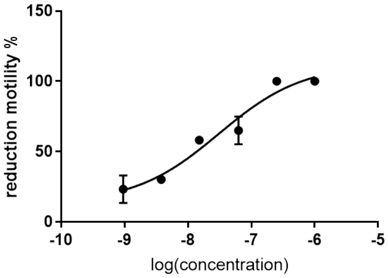

28

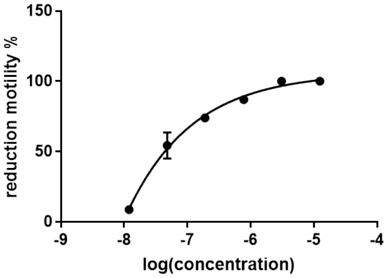

29

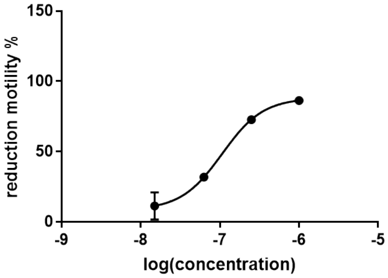

30

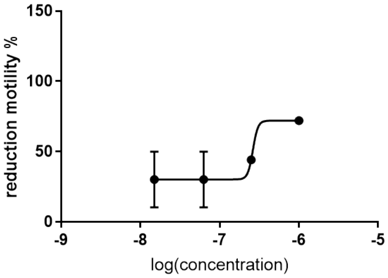

31

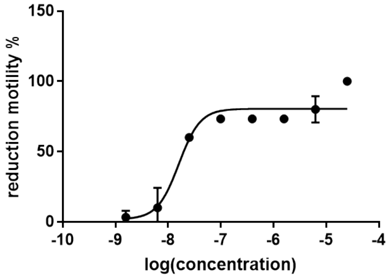

32

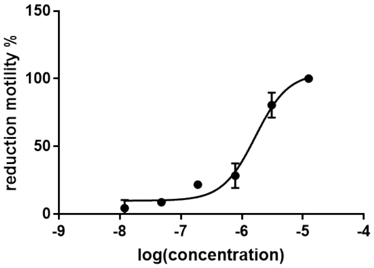

33

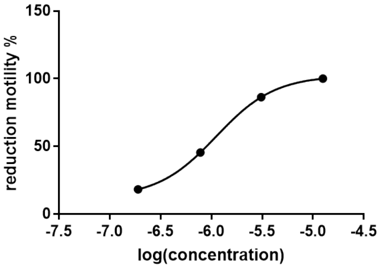

34

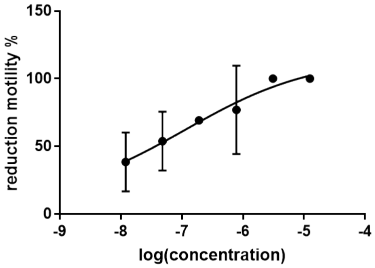

35

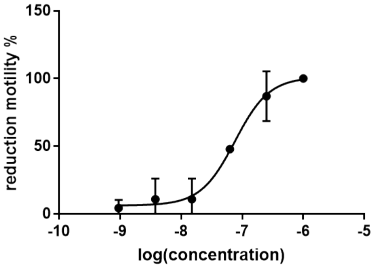

36

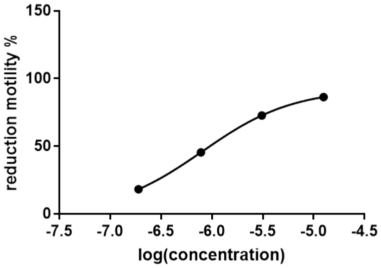

37

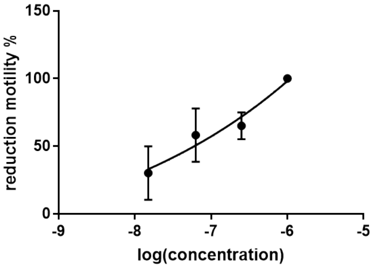

38

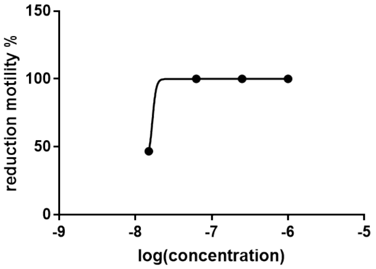

39

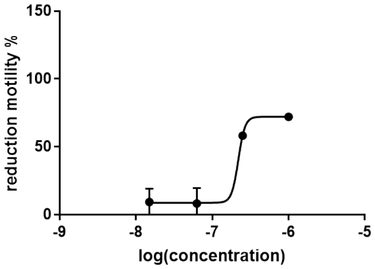

40

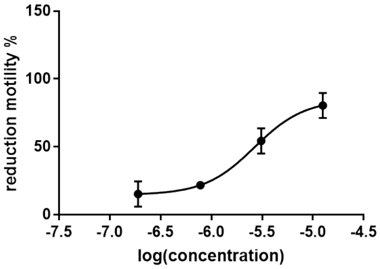

41

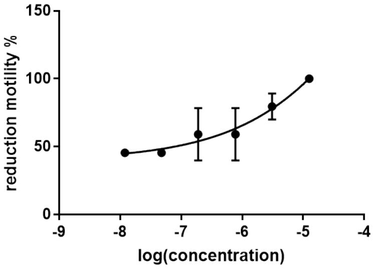

42

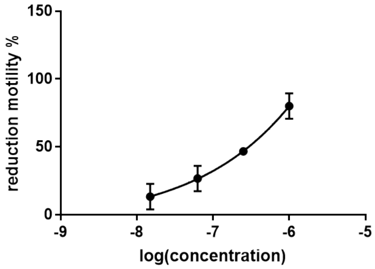

43

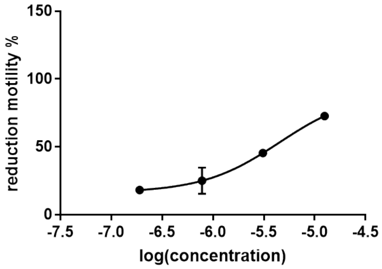

44

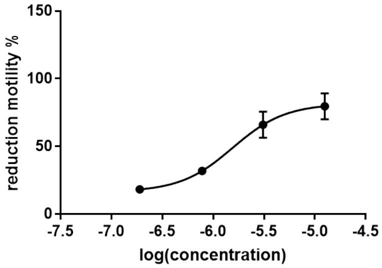

45

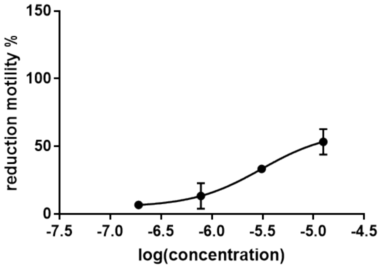

46

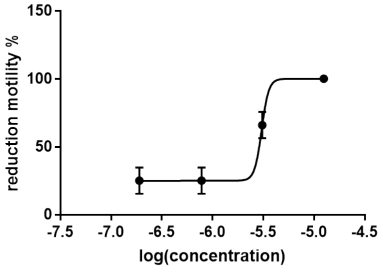

47

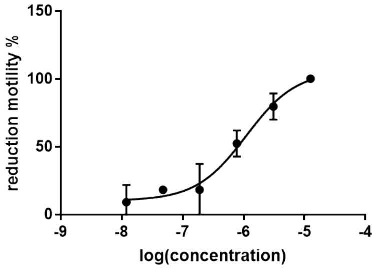

48

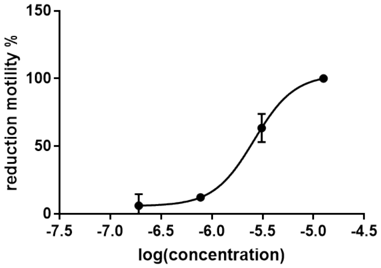

49

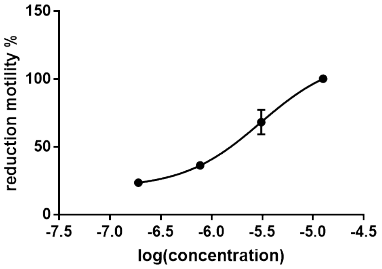

50

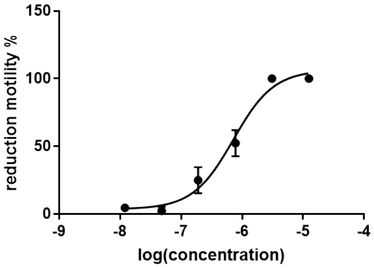

51

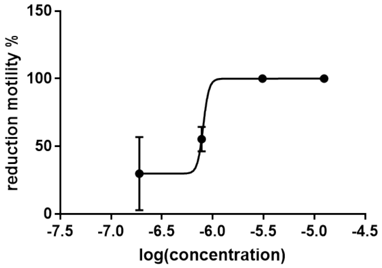

52

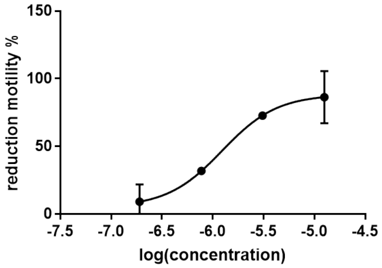

53

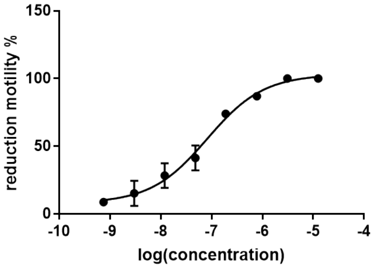

55

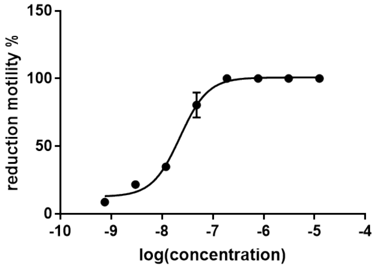

56

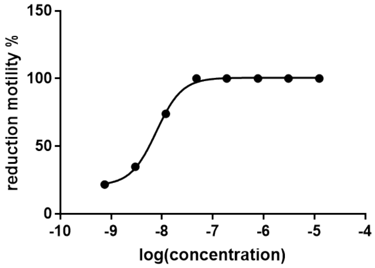

50

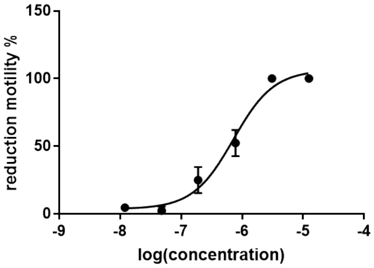

51

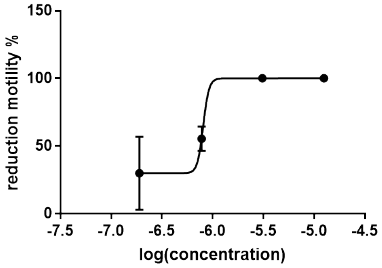

52

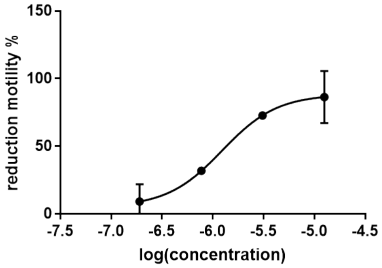

53

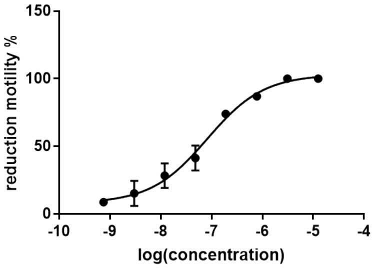

55

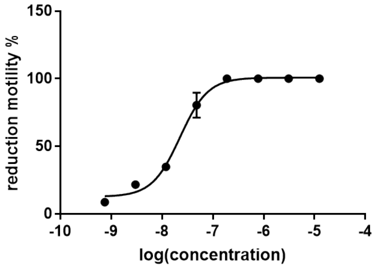

56

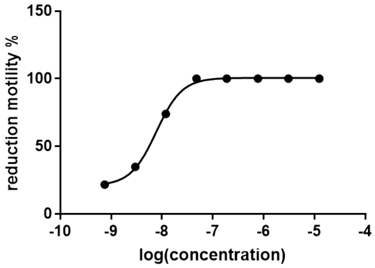

Data S2. Compound 31**,** Reduction of adult worm burden in patent *L. sigmodontis*-infected jirds

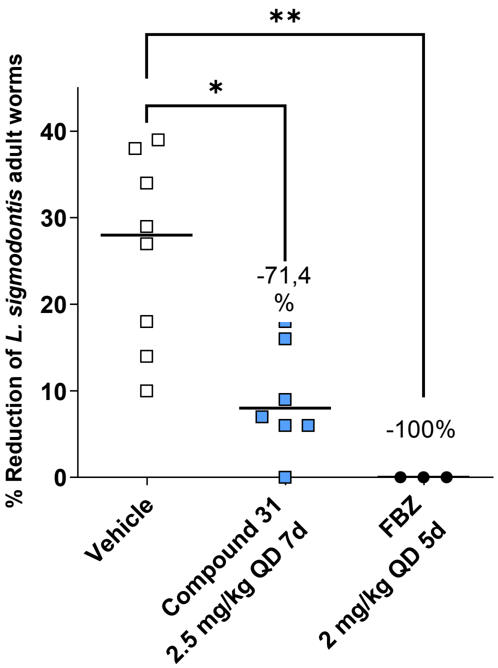

Plotted using Graph Pad Prism 7.03. Jirds have been naturally infected with *L. sigmodontis* for 12 weeks and then treated with vehicle for 7 days (7d) or subcutaneously once per day with 2.5mg/kg of 31 for 7 days or 2mg/kg flubendazole (FBZ) for 5 days. Percent reduction was determined from the mean of treatment groups and compared to mean of untreated controls. Analysis was done using unpaired 2 tailed t-test.

Data S3. ***O. gutturosa*** **motility and MTT viability inhibition EC_{50}, hERG IC_{50},** ***O. volvulus*** **L5 % inhibition. MTT/formazan viability data.**

Analog	*O. gutturosa* EC_{50} (uM) motility inhibition (SD)[a]	*O. gutturosa* EC_{50} (uM) MTT viability inhibition (SD)[a]	measured logD[b]	Kinetic solubility[c] (uM)	hERG IC_{50} (uM)	*O. volvulus* L5 % inhibition of motility Day 21 (1uM)	*O. volvulus* L5 % inhibition of viability Day 21 (1uM)	LE	LLE
1	0.27 (0.0)	0.94 (0.01)	NA	NA				0.45	
2	1.70 (0.84)	1.5 (0.02)		62.7				0.39	
3	0.03 (0.71)	0.24 (0.03)	2.2	7.3	19			0.40	5.37
4	>12.00 (0.43)	2.8 (0.02)	2.7	0.4				0.26	2.22
5	NA	NA	2.15	182				0.00	
6	0.07 (0.71)	4.1 (0.04)	1.7	57.5				0.37	5.44
7	0.94 (0.25)	6.5 (0.03)	1.55	12.8				0.32	4.48
8	0.01 (0.35)	0.004 (0.01)	2	201.7				0.42	6.05
9	0.16 (0.35)	1.1 (0.01)	1.8	8.2				0.52	5.01
10	0.06 (0.00)	0.01 (0.01)	2.88	3.2	>30			0.43	4.38
11	0.11 (0.91)	0.02 (0.02)	3.4	1.84	>30	100	100	0.41	3.57
12	0.39 (0.41)	0.03 (0.02)	2.1	13.08	0.20			0.29	4.31
13	5.80 (0.35)	20 (0.01)	2.75	0.55				0.34	2.49
14	3.10 (0.35)	4.5 (0.01)	2	2.55				0.36	3.51
15	1.37 (0.87)	0.71 (0.03)	2.3	0.3	>30			0.32	3.56
16	3.28 (0.35)	1.1 (0.02)	1.9	26.5				0.31	3.58
17	0.63 (0.35)	0.5 (0.02)	2.3	154.5				0.30	3.90
18	0.06 (0.87)	0.09 (0.03)	1.7	73.2	>30			0.41	5.51
19	0.06 (1.32)	0.19 (0.04)	3.3	3.05	3.46			0.34	3.89
20	0.24 (0.50)	0.3 (0.02)	2.8	NC	>30			0.29	3.83
21	0.04 (0.35)	0.14 (0.02)	2.65	2.17				0.42	4.71
22	0.20 (0.35)	13.1 (0.02)	2	184.2				0.35	4.70
23	0.04 (0.96)	0.012 (0.03)	2.5	0.2		100	100	0.40	4.92
24	0.05 (0.55)	0.75 (0.02)	2.9	1.05				0.34	4.39
25	0.10 (0.87)	0.54 (0.04)	2.85	2.58				0.38	4.15
26	0.03 (0.35)	0.07 (0.01)	2.45	0.9	>30			0.45	5.09
27	0.01 (0.41)	0.07 (0.03)	2.5	1.6				0.44	5.50
28	0.08 (0.29)	0.62 (0.01)	1.7	7.25				0.40	5.42
29	0.12 (0.35)	0.88 (0.02)	2.1	26.5				0.45	4.82

30	0.22 (1.00)	0.59 (0.05)	2.9	1.9	16			0.38	3.76
31	0.08 (0.61)	0.24 (0.01)	3.23	22.5	8	100	100	0.39	3.85
32	0.74 (0.50)	0.56 (0.04)	2.5	8.92		100	83	0.33	3.63
33	0.85 (0.00)	0.83 (0.04)	3	1.43				0.29	3.07
34	0.04 (1.19)	0.53 (0.03)	2.9	0				0.35	4.54
35	0.05 (1.04)	0.15 (0.04)	3	0				0.40	4.30
36	1.12 (0.00)	1.4 (0.01)	1.7	193.4				0.31	4.25
37	0.05 (1.06)	0.3 (0.03)	2.8	1				0.34	4.48
38	0.02 (0.00)	<0.02 (0.01)	2.95	1.03				0.37	4.82
39	0.29 (0.79)	1.1 (0.02)	3.1	2.1				0.39	3.44
40	2.35 (0.61)	2.9 (0.02)	2.9	12.3				0.33	2.73
41	0.06 (0.87)	<0.19 (0.01)	2.7	24.48				0.43	4.54
42	0.21 (0.61)	0.36 (0.01)	2.35	33.5				0.38	4.32
43	3.3 (0.25)	3.4 (0.02)	3.1	118.1				0.29	2.38
44	1.67 (0.35)	1.4 (0.02)	3	121				0.30	2.78
45	11.90 (0.5)	5.7 (0.01)	1.7	2.35				0.28	3.22
46	1.27 (0.61)	1.37 (0.02)	2.4	29.5	>30			0.31	3.50
47	1.07 (0.50)	1.9 (0.00)	1.62	109	>30	100	100	0.30	4.35
48	3.71 (0.50)	1.05 (0.02)	3.5	10.8	26			0.24	1.93
49	1.11 (0.35)	3.7 (0.04)	3.63	2.67	>30			0.25	2.32
50	0.56 (0.50)	2.3 (0.03)	2.4	2.71		100	100	0.29	3.86
51	0.49 (1.12)	0.46 (0.05)	2.2	15				0.31	4.11
52	1.73 (1.00)	1.95 (0.04)	2.7	9.95				0.25	3.06
53	0.05 (0.43)	0.15 (0.4)	2.2	4.05				0.37	5.13
54	0.14 (0.35)	0.15 (0.01)	3.13	13.14	>30			0.30	3.73
55	0.01 (0.25)	0.04 (0.02)	3	0.4				0.35	4.89
56	0.01 (0.00)	0.01 (0.01)	3.1	5.6	10			0.38	5.30
57	0.58 (0.00)	1.3 (0.05)	3.2	3.27				0.27	3.04
58	0.62 (0.00)	1.5 (0.01)	2.15	130.2				0.27	4.06
59	2.66 (0.87)	3.4 (0.02)	1.6	0.3	>30			0.30	3.98
60	0.67 (0.87)	2.4 (0.02)	1.8	10.15				0.32	4.37
61	0.36 (1.06)	2.1 (0.03)	2.1	100.7	>30			0.33	4.35

[a] Standard deviation, [b] LogD assay runs on 1M concentration range using 10 mM DMSO stock solutions [c] standard kinetic solubility assay runs on 200 mM concentration range using 10 mM DMSO stock solutions

Methods Procedures S1

LogD Measurement: Measurement of octanol-water distribution coefficient of samples using a partition factor. Standard LogD assay runs on 1M concentration range using 10 mM DMSO stock solutions for plating. Aqueous Phase: 100mM Buffer, pH=7.4 (0.1M Potassium phosphate buffer, diluted from Sigma 1M P3619-1GA to 0.1 M with MilliQ water), pH=2.2 (0.1M- NaCl pH adjusted with 0.1N HCL), pH=6.8 (0.1M - KH_2PO_4/K_2HPO_4), pH=5 (4 .04g of NaOH (Pellets), 8.65g of Glacial Acetic Acid, 11.874g of NaCl. In in 1 liter water. Adjusted to pH to 5 with either 1N NaOH or 1N HCl); Organic Phase: 1-Octanol (O021). Sample Concentration: 1000uM, 25 ml 10 mM DMSO stock solution diluted in 112.5 ml of organic Phase and 112.5 ml of aqueous Phase. DMSO content: 10%. The controls were plated from the standard 10 mM DMSO stock and run on the standard concentration (1M) range of the assay. Incubation time: Sonication for 30 minutes followed by 24 hours shaking at 750 rpm in 96 deep well plate (VWR cat# 40002-009) at room temperature. After incubation, centrifugation of the assay plate at 3600 rpm for 30 minutes.
Kinetic Solubility Measurement: The standard kinetic solubility assay runs on 200 mM concentration range using 10 mM DMSO stock solutions for plating. Single point calibration assay runs in duplicates. Concentration of standards: 100 mM, 100 mM DMSO solutions prepared by diluting 10 mM stock, solution with DMSO. Concentration of samples: 200 µM, 4 µl 10 mM DMSO stock solution diluted with the appropriate buffer to 200 ml. DMSO content: 2%. Buffers: pH=7.4 (0.1M Potassium phosphate buffer, diluted from Sigma 1M P3619-1GA to 0.1 M with MilliQ water), pH=2.2 (0.1M- NaCl pH adjusted with 0.1N HCL), pH=6.8 (0.1M - KH_2PO_4/K_2HPO_4), pH=5 (4 .04g of NaOH (Pellets), 8.65g of Glacial Acetic Acid, 11.874g of NaCl. In in 1 liter water. Adjusted to pH to 5 with either 1N NaOH or 1N HCl). The controls were plated from the standard 10 mM DMSO stock and run on the standard concentration (200µM) range of the assay. Incubation time: 24 hours shaking at 4500 rpm in Millipore Multiscreen HTS filter plate for Aqueous Solubility Assay (MSSLBPC50), on room temperature. After incubation time filtration with positive pressure manifold was performed.
Liver S9 Metabolic Stability Assay: Incubations were performed at 37 °C in a Dubnoff Shaking water bath using 2 mL 96-well incubation plates. Rat and human S9 protein concentrations were 0.75 mg/ml and 1.2 mg/ml, respectively. The final concentrations of NADPH, UDPGA, and GSH were 1, 0.5, and 2.5 mM, and the final concentration of PAPS was 0.05 mg/ml. Substrate concentrations were 3µM, incubation time was 60 minutes, and all tests were done in triplicate. The incubation was conducted in 200 mM Tris buffer containing 2 mM magnesium chloride, pH=7.4 and the total incubation volume was 0.5 ml. Samples were analysed via LC-MS/MS and reported as percentage remaining after incubation for 60 minutes.
Liver Microsome Metabolic Stability Assay: Incubations were performed at 37 °C in a Dubnoff Shaking water bath using 2 mL 96-well incubation plates. Liver microsome protein concentration was 0.3 mg/mL the final concentration of NADPH was 1 mM. Substrate concentrations are 3µM, incubation time is 30 minutes, and all tests are done in triplicate. The incubation was conducted in 200 mM Phosphate buffer containing 2 mM magnesium chloride, pH=7.4 and the total incubation volume was 0.5 mL. Samples were analyzed via LC-MS/MS and reported as percentage remaining after 30minute incubation, which can then be converted to half-life and intrinsic clearance.
MDCK-wt Permeability Assay: A ready-to use cell culture system that provides a 7-day cell barrier in integrated HTS Transwell®-96 plates purchased from ADMEcell were used for the

MDCK-wt assay. Polarized cultures of MDCKII cells were provided on polycarbonate micro-porous filters in HTS Transwell® plates (0.14 cm^2 area and 0.4 μm pore diameter). The transport medium used for the permeability studies was Hank's balanced salt solution (HBSS) buffer containing 1% BSA and 25 mM HEPES. Prior to the experiment, each monolayer was washed twice with warm buffer and treated for 30 minutes with elacridar at 2 μM to inhibit any efflux transporters. The concentration of test compound in this assay was 10 μM and all measurements were performed in duplicate. Lucifer yellow served as a quality control check for monolayer integrity of all wells and two control compounds were run with each assay (Atenolol and Metoprolol). Studies were initiated by adding an appropriate volume of buffer containing test compound to either the apical or basolateral side of the monolayer. The monolayers were placed into a standard cell culture incubator (5%CO_2, 37°C) for two hours. Samples were taken from both the apical and basolateral compartments at the end of the two-hour incubation and compound concentration was analyzed by LC-MS/MS. Permeability of compounds was determined as the coefficient of apparent permeability (Papp, measured in cm/s) calculated according to the following formula: Papp = dQ/ (dt·A·C0), where dQ/dt is the amount of compound present in the receiver compartment as a function of time; A is the area of the Transwell (cm^2); and C_0 is the initial concentration of compound applied in the donor compartment.

Experimental Procedures S1

Scheme S1 Synthetic routes to prepare additional compounds of Table 1.

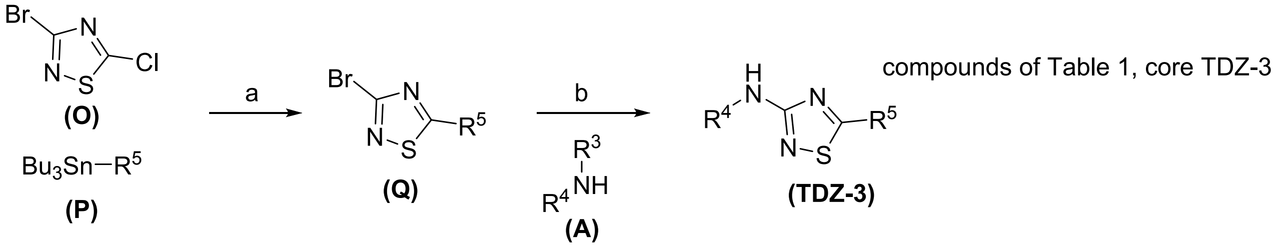

[a]Reagents and Conditions: a) $Pd(PPh_3)_4$, 2 eq CuI, Dioxane, 20 °C, 16 h, 44% b) Brettphos-Pd-G3, Cs_2CO_3, Dioxane, 90 °C, 16 h, 13%.

N-(3-Methylpyridin-2-yl)-5-(5-((tetrahydro-2h-pyran-4-yl)oxy)pyridin-2-yl)-1,2,4-thiadiazol-3-amine (5)

5-((Tetrahydro-2H-pyran-4-y (l)oxy)-2-(tributylstannyl)pyridine. To a solution of 2-bromo-5-((tetrahydro-2H-pyran-4-yl)oxy)pyridine (1. g, 3.87 mmol), tricyclohexylphosphine (108.65 mg, 0.390 mmol) in 1,4-dioxane (10 mL) was added tris(dibenzylideneacetone)dipalladium(0) (177.39 mg, 0.190 mmol) under nitrogen. 1,1,1,2,2,2-hexabutyldistannane (3.14 g, 5.42 mmol) was added to the mixture dropwise under nitrogen and the mixture was stirred at 100 °C for 16 h. The mixture was poured into water and the aqueous phase was extracted with ethyl acetate. The combined organic phases were washed with brine, dried over anhydrous sodium sulfate, filtered

and concentrated under vacuum. The residue was purified by silica gel chromatography to give crude 5-((tetrahydro-2H-pyran-4-yl)oxy)-2-(tributylstannyl)pyridine (250 mg, 0.5339 mmol, 13.8% yield). LCMS (ESI): m/z 470.2 $[M+1]^+$

3-Bromo-5-(5-((tetrahydro-2H-pyran-4-yl)oxy)pyridin-2-yl)-1,2,4-thiadiazole. To a solution of 5-((tetrahydro-2H-pyran-4-yl)oxy)-2-(tributylstannyl)pyridine (1.4 g, 2.99 mmol) in 1,4-dioxane (10 mL) was added 3-bromo-5-chloro-1,2,4-thiadiazole (596.34 mg, 2.99 mmol) followed by de-gassing with nitrogen for 3 min. Tetrakis[triphenylphosphine]palladium(0) (345.49 mg, 0.300 mmol) and CuI (1.14 g, 5.98 mmol) were added to the mixture and stirred at 20 °C for 16 h under nitrogen. The mixture was concentrated under vacuum. The residue was purified by flash silica gel chromatography to give 3-bromo-5-(5-((tetrahydro-2H-pyran-4-yl)oxy)pyridin-2-yl)-1,2,4-thiadiazole (500 mg, 1.30 mmol, 43.4% yield). LCMS (ESI): m/z 341.8 $[M+1]^+$

N-(3-Methylpyridin-2-yl)-5-(5-((tetrahydro-2H-pyran-4-yl)oxy)pyridin-2-yl)-1,2,4-thiadiazol-3-amine. To a solution of 3-bromo-5-(5-((tetrahydro-2H-pyran-4-yl)oxy)pyridin-2-yl)-1,2,4-thiadiazole (200 mg, 0.580 mmol) and 3-methylpyridin-2-amine (126.4 mg, 1.17 mmol) in 1,4-dioxane (5 mL) were added methanesulfonato(2-dicyclohexylphosphino-3,6-dimethoxy-2,4,6-tri-i-propyl-1,1-biphenyl)(2-amino-1,1-biphenyl-2-yl)palladium(II) (105.96 mg, 0.120 mmol) and cesium carbonate (571 mg, 1.75 mmol) under nitrogen. The mixture was stirred at 90 °C for 16 h. and then filtered through a pad of silica gel (100-200 mesh). The filtrate was purified using prep-HPLC (column: Phenomenex Gemini-NX C18 75*30mm*3um;mobile phase: [water(0.225%FA) -ACN];B%: 12%-42%,10min), and dried by lyophilization to give N-(3-methylpyridin-2-yl)-5-(5-((tetrahydro-2H-pyran-4-yl)oxy)pyridin-2-yl)-1,2,4-thiadiazol-3-amine (43.35 mg, 0.114 mmol, 19.4 % yield). ^{1}H NMR (400 MHz, DMSO-d_6) δ 9.65 (s, 1H), 8.43 (d, J = 2.7 Hz, 1H), 8.16 (d, J = 3.5 Hz, 1H), 7.98 (d, J = 8.7 Hz, 1H), 7.67 (dd, J_1 = 8.8, J_2 = 2.8 Hz, 1H), 7.60 (d, J = 6.6 Hz, 1H), 7.04 (dd, J_1 = 7.3, J_2 = 4.8 Hz, 1H), 4.83-4.78 (m, 1H), 3.88-3.85 (m, 2H), 3.53-3.48 (m, 2H), 2.25 (s, 3H), 2.04-2.01 (m, 2H), 1.66-1.63 (m, 2H). LCMS (ESI): m/z 370.2 $[M+1]^+$

2-(5-((3-Methylpyridin-2-yl)amino)-1,2,4-thiadiazol-3-yl)isonicotinonitrile (14)

Methyl 2-(imino(methoxy)methyl)isonicotinate. To a solution of methyl 2-cyanoisonicotinate (4 g, 24.7 mmol) in methanol (60 mL) was added sodium methanolate (400 mg, 7.40 mmol). The mixture was stirred at 25 °C for 16 h. and then used in the next step without workup or purification (4.60 g, crude) in methanol (60 mL).

Methyl 2-carbamimidoylisonicotinate hydrochloride. To the mixture of methyl 2-(imino(methoxy)methyl)isonicotinate and 2-(imino-(methoxy)methyl)isonicotinic acid (4.60 g, crude) in methanol (60 mL) was added ammonium chloride (723 mg, 13.5 mmol) at 70 °C and stirred for 2 h. The mixture was then filtered and the filtrate was concentrated under reduced pressure to remove the solvent to give methyl 2-carbamimidoylisonicotinate hydrochloride (6.00 g, crude, HCl).

Methyl 2-(5-((3-methylpyridin-2-yl)amino)-1,2,4-thiadiazol-3-yl)isonicotinate. To a solution of methyl 2-carbamimidoylisonicotinate hydrochloride (1.50 g, 6.96 mmol, HCl) in dichloromethane (20 mL) and acetone (20 mL) were added 2-isothiocyanato-3-methylpyridine

(2.09 g, 13.9 mmol) and triethylamine (2.11 g, 20.9 mmol) at 25 °C and stirred for 16 h. Triethylamine (1.41 g, 13.9 mmol) was then added to the reaction mixture and stirred at 25 °C for an additional 5 h. Triethylamine (3.52 g, 34.8 mmol) was added to the reaction mixture and stirred at 25 °C for 3 h. The reaction mixture was concentrated under reduced pressure to give a residue which was diluted with water and ethyl acetate. The mixture was filtered and the filter cake was dried to give 0.3 g of cyclized product methyl 2-(5-((3-methylpyridin-2-yl)amino)-1,2,4-thiadiazol-3-yl)isonicotinate. The filtrate was extracted with ethyl acetate. The combined organic layers were washed with brine, dried over anhydrous sodium sulfate, filtered, and concentrated under reduced pressure to give a crude product. The crude product was purified by column chromatography to give 0.8 g of cyclized product methyl 2-(5-((3-methylpyridin-2-yl)amino)-1,2,4-thiadiazol-3-yl)isonicotinate and coupling product methyl 2-(N-((3-methylpyridin-2-yl)carbamothioyl)carbamimidoyl)isonicotinate (280 mg, 0.553 mmol, 8% yield, 65% purity). The two portions of cyclized product were combined to give methyl 2-(5-((3-methylpyridin-2-yl)amino)-1,2,4-thiadiazol-3-yl)isonicotinate (1.10 g, 2.69 mmol, 39% yield, 80% purity).

2-(5-((3-Methylpyridin-2-yl)amino)-1,2,4-thiadiazol-3-yl)isonicotinamide. To a mixture of methyl 2-(5-((3-methylpyridin-2-yl)amino)-1,2,4-thiadiazol-3-yl)isonicotinate (320 mg, 0.978 mmol) in dimethylsulfoxide (5 mL) was added ammonium hydroxide (4.55 g, 38.9 mmol, 5 mL, 30% purity). The mixture was stirred at 50 °C for 16 h and then filtered. The filtered cake was triturated with methanol (5 mL) to give 2-(5-((3-methylpyridin-2-yl)amino)-1,2,4-thiadiazol-3-yl)isonicotinamide (180 mg, 0.346 mmol, 35% yield, 60% purity).

2-(5-((3-Methylpyridin-2-yl)amino)-1,2,4-thiadiazol-3-yl)isonicotinonitrile. To a solution of 2-(5-((3-methylpyridin-2-yl)amino)-1,2,4-thiadiazol-3-yl)isonico-tinamide (180 mg, 0.346 mmol) in dichloromethane (5 mL) was added triethylamine (350 mg, 3.46 mmol, 0.480 mL) at 0 °C followed by trifluoroacetic anhydride (363 mg, 1.73 mmol, 0.240 mL) under nitrogen. The mixture was stirred at 25 °C for 3 h. Saturated sodium bicarbonate was added to the reaction mixture and then diluted with water and dichloromethane. The organic phase was separated, washed with brine, dried over anhydrous sodium sulfate, filtered, and concentrated under reduced pressure to give a residue. The residue was triturated with acetonitrile (5 mL) and filtered. The filter cake was dried under reduced pressure and was diluted with acetonitrile (3 mL) and water (20 mL) and dried by lyophilization to give 2-(5-((3-methylpyridin-2-yl)amino)-1,2,4-thiadiazol-3-yl)isonicotinonitrile (89.76 mg, 0.293 mmol, 85% yield, 96.1% purity). ^{1}H NMR (400MHz, DMSO-d_6) δ 11.93 (s, 1H), 8.95 (dd, J_1 = 5.0, J_2 = 0.8 Hz, 1H), 8.53-8.50 (m, 1H), 8.34 (dd, J_1 = 5.0, J_2 =0.9 Hz, 1H), 7.96 (dd, J_1 = 4.9, J_2 = 1.5 Hz, 1H), 7.73-7.67 (m, 1H), 7.06 (dd, J_1 = 7.3, J_2 = 5.0 Hz, 1H), 2.41 (s, 3H); MS (ESI): *m/z* 295.2 $[M+1]^+$.

5-Isopropoxy-2-(5-((3-methylpyridin-2-yl)amino)-1,2,4-thiadiazol-3-yl)isonicotinonitrile (15)

5-Isopropoxy-2-(5-((3-methylpyridin-2-yl)amino)-1,2,4-thiadiazol-3-yl)isonicotinonitrile. To a solution of 5-isopropoxy-2-(5-((3-methylpyridin-2-yl)amino)-1,2,4- thiadiazol-3-yl)isonicotinamide (0.4 g, 0.864 mmol) and triethylamine (72.70 mg, 0.718 mmol, 0.1 mL) in dichloromethane (20 mL) was added trifluoroacetic anhydride (755.00 mg, 3.59 mmol, 0.5 mL) at 0 °C. The mixture was stirred at 25 °C for 4 h. and then at 40 °C for 2 h. To the mixture was

added saturate sodium bicarbonate and the aqueous phase was extracted with dichloromethane. The combined organic phase was concentrated under vacuum to give a residue and the residue was purified by prep-HPLC (column: Phenomenex Synergi C18 150*25*10um;mobile phase: [water(0.225%FA)-ACN];B%: 45%-75%,10min) follow by lyophilization to give 5-isopropoxy-2-(5-((3-methylpyridin-2-yl)amino)-1,2,4-thiadiazol-3-yl)isonicotinonitrile (162.97 mg, 0.462 mmol, 53.5% yield, 99.9% purity). ^{1}H NMR (400MHz, DMSO-d_6) δ 11.83 (s, 1H), 8.80 (s, 1H), 8.41 (s, 1H), 8.32 (d, J = 4.0 Hz, 1H), 7.69 (d, J = 7.2 Hz, 1H), 7.05 (dd, J_1 = 7.3, J_2 = 5.1 Hz, 1H), 5.15-5.04 (m, 1H), 2.40 (s, 3H), 1.40 (d, J = 6.0 Hz, 6H), MS (ESI): m/z 353.3 [M+1]$^+$.

5-Isopropoxy-N,N-dimethyl-2-(5-((3-methylpyridin-2-yl)amino)-1,2,4-thiadiazol-3-yl)isonicotinamide (**17**)

2-Bromo-5-isopropoxy-N,N-dimethylisonicotinamide. To a mixture of 2-bromo-5-isopropoxyisonicotinic acid (2 g, 7.69 mmol) and oxalyl chloride (2.17 g, 17.14 mmol, 1.50 mL) in DCM (30 mL) was added DMF (9.50 mg, 0.129 mmol, 0.01 mL) at 25 °C. The mixture was stirred at 25 °C for 1 h. and was concentrated under vacuum to give a residue. To the residue in DCM (30 mL) was added TEA (2.33 g, 23.07 mmol, 3.21 mL) and dimethylamine hydrochloride (0.8 g, 9.81 mmol, 0.898 mL) at 0 °C, the mixture was stirred at 25 °C for 1 h and was concentrated under vacuum to give a residue. The residue was purified by silica gel column chromatography to give 2-bromo-5-isopropoxy-N,N-dimethylisonicotinamide (2.1 g, crude).

2-Cyano-5-isopropoxy-N,N-dimethylisonicotinamide. To a mixture of 2-bromo-5-isopropoxy-N,N-dimethylisonicotinamide (2 g, crude), zinccyanide (1.64 g, 13.93 mmol) and zinc (90.00 mg, 1.38 mmol) in DMF (30 mL) was added tetrakis(triphenylphosphine)palladium (1.61 g, 1.39 mmol), and the mixture was stirred at 120 °C for 14 h under nitrogen. The mixture was filtered through a pad of celite and the filtrate was poured into water and extracted with EtOAc. The combined organic phases were washed with brine, and concentrated under vacuum to give a residue. The residue was purified by prep-HPLC (column: Phenomenex Synergi Max-RP 250*50mm*10 µm;mobile phase: [water(0.225%FA)-ACN];B%: 25ACN%-55ACN%, 28min, 60%min) and followed by lyophilization to give 2-cyano-5-isopropoxy-N,N-dimethylisonicotinamide (0.8 g, 3.43 mmol, 49.2% yield).

2-Carbamimidoyl-5-isopropoxy-N,N-dimethylisonicotinamide. A mixture of sodium (47 mg, 2.04 mmol) in MeOH (10 mL) was stirred at 25 °C for 10 min. To the mixture was added 2-cyano-5-isopropoxy-N,N- dimethylisonicotinamide (0.8 g, 3.43 mmol) at 25 °C, the mixture was stirred at 40 °C for 4 h. Then to the mixture was added ammonium chloride (0.29 g, 5.42 mmol), the mixture was stirred at 70 °C for 1.5 h and was concentrated under vacuum to give a residue. The residue was triturated with MTBE (20 mL), the precipitate was collected by filtration and dried under high vacuum to give 2-carbamimidoyl-5-isopropoxy-N,N-dimethylisonicotinamide hydrochloride (0.98 g, 3.42 mmol, 99.6% yield).

5-Isopropoxy-N,N-dimethyl-2-(N-((3-methylpyridin-2-yl)carbamothioyl)carbamimidoyl)isonicotinamide. To a mixture of 2-carbamimidoyl-5-isopropoxy-N,N-dimethylisonicotinamide (0.3 g, 1.05 mmol) and 2-isothiocyanato-3-methylpyridine (0.19 g, 1.14 mmol) in acetone (30 mL) and DCM (30 mL) was added TEA (1.06 g, 10.46 mmol, 1.46 mL). The mixture was stirred at 25 °C for 3 h. To the mixture was added 2-isothiocyanato-3-methylpyridine (0.08 g, 0.53 mmol) and TEA (1.06 g, 10.46 mmol, 1.46 mL). Then the mixture was stirred at 25 °C for 12 h. The mixture was then stirred at 40 °C for 6 h and concentrated

under vacuum to give a crude product. The crude product was used directly in the next step. 5-isopropoxy-N,N-dimethyl-2-(N-((3-methyl-pyridin-2-yl)carbamothioyl)carbamimidoyl)isonicotinamide (0.7 g, crude).

5-Isopropoxy-N,N-dimethyl-2-(5-((3-methylpyridin-2-yl)amino)-1,2,4-thiadiazol-3-yl)isonicotinamide. To a mixture of 5-isopropoxy-N,N-dimethyl-2-(N-((3-methyl-pyridin-2-yl)carb-amothioyl)carbamimidoyl)isonicotinamide (0.7 g, 1.75 mmol) in EtOH (40 mL) was added iodine (88.00 mg, 346.72 μmol) and hydrogen peroxide (413.0 mg, 3.64 mmol, 0.35 mL, 30% purity) at 0 °C. The mixture was stirred at 25 °C for 40 min. The mixture was quenched with saturate sodium sulfite (20 mL) and concentrated under vacuum to give an aqueous phase. The aqueous phase was extracted with DCM. The combined organic phases were concentrated under vacuum to give a residue. The product was isolated and purified by column chromatography to give 5-isopropoxy-N,N-dimethyl-2-(5-((3-methylpyridin-2-yl)amino)-1,2,4-thiadiazol-3-yl)isonicotinamide (134.84 mg, 335.68 μmol, 19.2% yield, 99.2% purity). ^{1}H NMR (400MHz, DMSO-d_6) δ 11.73 (s, 1H), 8.54 (s, 1H), 8.31 (d, J = 4.0 Hz, 1H), 8.02 (s, 1H), 7.68 (d, J = 6.6 Hz, 1H), 7.04 (dd, J_1 = 7.3 Hz, J_2 = 5.0 Hz, 1H), 4.94-4.88 (m, 1H), 3.01 (s, 3H), 2.81 (s, 3H), 2.40 (s, 3H), 1.32 (d, J = 6.0 Hz, 6H). MS (ESI): m/z 399.1 $[M+1]^+$.

3-(5-Methoxypyridin-2-yl)-N-(5-((tetrahydro-2H-pyran-4-yl)oxy)pyridin-2-yl)-1,2,4-thiadiazol-5-amine (22)

1'-Methyl-1',2',3',6'-tetrahydro-[4,4'-bipyridine]-2-carbonitrile. To a mixture of 4-bromopicolinonitrile (1 g, 5.46 mmol) and 1-methyl-4-(4,4,5,5-tetramethyl-1,3,2-dioxaborolan-2-yl)-1,2,3,6-tetrahydropyridine (1.46 g, 6.55 mmol) in water (16 mL) and dioxane (80 mL) were added sodium carbonate (1.45 g, 13.7 mmol) and palladium triphenylphosphine (316 mg, 0.273 mmol) under nitrogen. The mixture was stirred at 80 °C for 16 h. The mixture was poured into water (100 mL). The aqueous phase was extracted with ethyl acetate. The combined organic phase was washed with brine, dried over anhydrous sodium sulfate, filtered, and concentrated in vacuo. The residue was purified by silica gel chromatography to give 1'-methyl-1',2',3',6'-tetrahydro-[4,4'-bipyridine]-2-carbonitrile (1.8 g, 9.03 mmol, 83% yield).

1'-Methyl-1',2',3',6'-tetrahydro-[4,4'-bipyridine]-2-carboximidamide hydrochloride. To a solution of 1'-methyl-1',2',3',6'-tetrahydro-[4,4'-bipyridine]-2-carbonitrile (1.30 g, 6.52 mmol) in methanol (65 mL) was added sodium methanolate (106 mg, 1.96 mmol). The mixture was stirred at 25 °C for 16 h under nitrogen. Ammonium chloride (698 mg, 13.0 mmol) was added into the mixture and stirred at 70 °C for 4 h. The mixture was concentrated in vacuo and the residue triturated with dichloromethane. The mixture was filtered, and the filter cake was dried in vacuo to give 1'-methyl-1',2',3',6'-tetrahydro-[4,4'-bipyridine]-2-carboximidamide (1.5 g, crude, HCl).

4-(1-Methylpiperidin-4-yl)picolinimidamide. To a solution of 1'-methyl-1',2',3',6'-tetrahydro-[4,4'-bipyridine]-2-carboximidamide hydrochloride (1.90 g, 7.52 mmol) in methanol (50 mL) was added Pd/C (190 mg, 15% purity). The mixture was stirred at 50 °C for 6 h under hydrogen balloon (15psi) and then filtered through celite. The filtrate was concentrated in vacuo to give 4-(1-methylpiperidin-4-yl)picolinimidamide hydrochloride (1.25 g, crude) 1H NMR (400 MHz, DMSO-*d6*) δ 8.76 (d, J = 5.0 Hz, 1H), 8.30 (s, 1H), 7.69 (d, J = 4.8 Hz, 1H), 3.16-2.92 (m, 5H), 2.77 (s, 3H), 2.16-1.97 (m, 4H). The obtained compound in methanol (50 mL) was adjusted to pH ~10 by addition of AMBERLYST A 26 (CAS 39339-85-0). The mixture was stirred at 25 °C

for 3 h and then filtered. The filtrate was concentrated to give 4-(1-methylpiperidin-4-yl)picolinimidamide (1.25 g, crude) which was used directly in the next step.

4-(1-Methylpiperidin-4-yl)-N-((3-methylpyridin-2-yl)carbamothioyl) picolinimidamide. To a mixture of 4-(1-methylpiperidin-4-yl)picolinimidamide (917 mg, 4.20 mmol) and triethylamine (4.25 g, 42.0 mmol) in acetone (15 mL) and dichloromethane (15 mL) was added 2-isothiocyanato-3-methylpyridine (757 mg, 5.04 mmol). The mixture was stirred at 25°C for 16 h under nitrogen and then concentrated in vacuo. The residue was diluted with water and the aqueous phase was extracted with ethyl acetate. The combined organic phase was washed with brine, dried over anhydrous sodium sulfate, filtered, and concentrated in vacuo to give the crude product. The crude product was purified by prep-TLC to give a mixture of 4-(1-methylpiperidin-4-yl)-N-((3-methylpyridin-2-yl)carbamothioyl) picolinimidamide and 3-(4-(1-methylpiperidin-4-yl)pyridin-2-yl)-N-(3-methylpyridin-2-yl)-1,2,4-thiadiazol-5-amine (590 mg, 0.442 mmol, 21% yield, 55% purity).

3-(5-Methoxypyridin-2-yl)-N-(5-((tetrahydro-2H-pyran-4-yl)oxy)pyridin-2-yl)-1,2,4-thiadiazol-5-amine. To a solution of 5-methoxy-N-((5-((tetrahydro-2H-pyran-4-yl)oxy)pyridin-2 yl)carbamothioyl) picolinimidamide and 3-(5-methoxypyridin-2-yl)-N-(5-((tetrahydro-2H-pyran-4-yl)oxy)pyridin-2-yl)-1,2,4-thiadiazol-5-amine (490 mg, 0.367 mmol) in ethanol (25 mL) was added hydrogen peroxide (83.1 mg, 0.733 mmol, 30% purity) and iodine (18.6 mg, 0.073 mmol). The mixture was stirred at 25 °C for 3 h. The residue was diluted with water and saturated aqueous sodium sulfite and stirred at 25 °C for an additional 0.5 h. The reaction was checked by potassium iodide-starch test paper to determine hydrogen peroxide consumption. The aqueous phase was extracted with ethyl acetate and the combined organic phases were dried over anhydrous sodium sulfate, filtered, and concentrated under vacuum. The residue was purified by prep-HPLC to give 3-(4-(1-methylpiperidin-4-yl)pyridin-2-yl)-N-(3-methylpyridin-2-yl)-1,2,4-thiadiazol-5-amine (277.82 mg, 0.645 mmol, 88% yield, 95.7% purity, FA). ^{1}H NMR (400 MHz, CD_3OD) δ 8.62 (d, J = 5.0 Hz, 1H), 8.54 (s, 0.4H), 8.38-8.27 (m, 2H), 7.65 (d, J = 7.3 Hz, 1H), 7.43 (d, J = 5.0 Hz, 1H), 7.11-6.97 (m, 1H), 3.39-3.32 (m, 2H), 2.98-2.82 (m, 1H), 2.75-2.60 (m, 5H), 2.44 (s, 3H), 2.14-2.05 (m, 2H), 2.03-1.85 (m, 2H); MS (ESI): m/z 367.1 $[M+1]^+$.

3-(4-Isopropylpyridin-2-yl)-N-(3-(trifluoromethyl)pyridin-2-yl)-1,2,4-thiadiazol-5-amine (27)

4-(Prop-1-en-2-yl)picolinonitrile. To a mixture of 4-bromopicolinonitrile (2.00 g, 10.9 mmol) and potassium trifluoro(prop-1-en-2-yl)borate (3.23 g, 21.9 mmol) in 1,4-dioxane (20 mL) and water (2 mL) was added potassium carbonate (943 mg, 6.82 mmol) and dichloro(1,1'-bis(diphenylphosphanyl)ferrocene)palladium(II) chloroform complex (800 mg, 1.09 mmol) under nitrogen. The mixture was stirred at 80 °C for 4 h. The mixture was poured into water. The aqueous phase was extracted with ethyl acetate and the combined organic phase was washed with brine, dried over anhydrous sodium sulfate, filtered, and concentrated in vacuo. The residue was purified by column chromatography to give 4-(prop-1-en-2-yl)picolinonitrile (1.44 g, 9.99 mmol, 91.3% yield).

4-(Prop-1-en-2-yl)picolinimidamide hydrochloride. To a mixture of 4-(prop-1-en-2-yl)picolinonitrile (1.65 g, 11.4 mmol) in methanol (15 mL) was added sodium methanolate (185 mg, 3.43 mmol). The mixture was stirred at 30 °C for 16 h under nitrogen. Ammonium chloride

(796 mg, 14.9 mmol) was added into the above mixture. The mixture was stirred at 70 °C for 3 h. The mixture was concentrated in vacuo and the residue was diluted with ethanol (80 mL). The mixture was refluxed at 80 °C for 15 min. The mixture was filtered, and the filter cake was triturated with dichloromethane (30 mL). The filter cake was dried in vacuo to give 4-(prop-1-en-2-yl)picolinimidamide hydrochloride (1.70 g, 8.60 mmol, 75% yield).

4-Isopropylpicolinimidamide hydrochloride. To a solution of 4-(prop-1-en-2-yl)picolinimidamide hydrochloride (1.60 g, 8.09 mmol) in 2,2,2-trifluoroethanol (80 mL) was added Pd/C (480 mg). The mixture was stirred at 20 °C for 2.5 h under hydrogen balloon (15 Psi). The mixture was filtered through celite and the filtrate was concentrated in vacuo to give 4-isopropylpicolinimidamide hydrochloride (1.60 g, 8.01 mmol, 99.1% yield).

4-Isopropylpicolinimidamide. To a solution of 4-isopropylpicolinimidamide hydrochloride (1.80 g, 9.01 mmol) in acetonitrile (75 mL) and water (15 mL) was added AMBERLYST A 26 (CAS 39339-850). The pH of the mixture was adjusted to 10-11 and stirred at 20 °C for 4 h. The reaction mixture was filtered and the filtrate was concentrated in vacuo followed by lyophilization to give 4-isopropylpicolinimidamide (1.40 g, 8.49 mmol, 94 % yield, 99% purity).

4-Isopropyl-N**-((3-(trifluoromethyl)pyridin-2-yl)carbamothioyl)picolinimidamide.** To a solution of 4-isopropylpyridine-2-carboxamidine (500 mg, 3.03 mmol) in dichloromethane (5 mL) and acetone (5 mL) were added triethylamine (3.07 g, 30.30 mmol) and 2-isothiocyanato-3-(trifluoromethyl)pyridine (635 mg, 3.03 mmol). The mixture was stirred at 15 °C for 1 h and then concentrated. The residue was poured into water, and aqueous phase was extracted with ethyl acetate. The combined organic phases were dried over anhydrous sodium sulfate, filtered, and concentrated under vacuum. The crude product 4-isopropyl-N-((3-(trifluoromethyl)pyridin-2-yl)carbamothioyl)picolinimidamide (1.00 g, 1.90 mmol, 62.6% yield, 69.7% purity) was used in the next step without further purification.

3-(4-Isopropylpyridin-2-yl)-N-(3-(trifluoromethyl)pyridin-2-yl)-1,2,4-thiadiazol-5-amine. To a solution of 4-isopropyl-N-((3-(trifluoromethyl)pyridin-2-yl)carbamothioyl) picolinimidamide (1.00 g, 1.90 mmol) in ethanol (20 mL) was added iodine (96 mg, 0.379 mmol) and hydrogen peroxide (129 mg, 3.79 mmol). The mixture was stirred at 15 °C for 1 h under N_2. The mixture was diluted with water mL and saturated aqueous sodium sulfite and stirred at 0 °C for another 0.5 h. The aqueous phase was extracted with ethyl acetate and the combined organic phase was dried over anhydrous sodium sulfate, filtered, and concentrated under vacuum. The residue was purified by prep-HPLC to give 3-(4-isopropyl-2-pyridyl)-N-[3-(trifluoromethyl)-2-pyridyl]-1,2,4-thiadiazol-5-amine (373.04 mg, 0.975 mmol, 51.3% yield, 95.5% purity). ^{1}H NMR (400MHz, DMSO-d_6) δ 8.71 (d, J = 4.0 Hz, 1H), 8.58 (d, J = 5.0 Hz, 1H), 8.19-8.11 (m, 2H), 7.38 (d, J = 3.7 Hz, 1H), 7.15 (s, 1H), 3.08-2.94 (m, 1H), 1.27 (d, J = 7.0 Hz, 6H); MS (ESI): m/z 366.1 $[M+1]^+$.

N2-(3-(5-isopropoxypyridin-2-yl)-1,2,4-thiadiazol-5-yl)-N3-methylpyridine-2,3-diamine (30).

tert-Butyl N-tert-butoxycarbonyl-N-(2-nitro-3-pyridyl)carbamate. To a solution of 2-nitropyridin-3-amine (5 g, 35.94 mmol, 1 eq) in THF (150 mL) was added NaHMDS (1 M, 53.91 mL) at 0 °C. The mixture was stirred at 20 °C for 1 h. Then di-tert-butyl dicarbonate (8.63

g, 39.54 mmol) was added at 0 °C. The mixture was stirred at 25 °C for 16 h. The mixture was poured into ice-water. The aqueous phase was extracted with ethyl acetate. The combined organic phase was dried over anhydrous sodium sulfate, filtered and concentrated under vacuum. The residue was purified by column chromatography to give tert-butyl N-tert-butoxycarbonyl-N-(2-nitro-3-pyridyl)carbamate (7 g, 18.57 mmol, 51.65% yield, 90% purity).

tert-Butyl (2-nitropyridin-3-yl)carbamate. To a solution of tert-butyl N-tert-butoxycarbonyl-N-(2-nitro-3-pyridyl)carbamate (7 g, 20.63 mmol, 1 eq) in THF (30 mL) was added sodium hydroxide/water (2 M, 100.05 mL). The mixture was stirred at 50 °C for 16 h. The mixture was poured into water (100 mL). The aqueous phase was extracted with dichloromethane. The combined organic phase was dried over anhydrous sodium sulfate, filtered and concentrated under vacuum. The residue was purified by column chromatography to give tert-butyl (2-nitropyridin-3-yl)carbamate (3 g, 12.54 mmol, 60.8% yield).

tert-Butyl methyl(2-nitropyridin-3-yl)carbamate. To a solution of tert-butyl N-(2-nitro-3-pyridyl)carbamate (2.2 g, 9.20 mmol) in DMF (25 mL) was added sodium hydrate (735 mg, 18.39 mmol, 60% purity) at 0 °C in portions. The mixture was stirred at 25 °C for 0.5 h. Then methyl iodide (1.96 g, 13.79 mmol) was added at 0 °C. The mixture was stirred at 25 °C for 2.5 h. The mixture was poured into ice-water (100 mL) .The aqueous phase was extracted with ethyl acetate. The combined organic phase was dried over anhydrous sodium sulfate, filtered and concentrated under vacuum. The residue was purified by silica gel chromatography to give tert-butyl methyl(2-nitropyridin-3-yl)carbamate (2.3 g, 9.08 mmol, 98.8% yield).

tert-Butyl (2-aminopyridin-3-yl)(methyl)carbamate. To a solution of tert-butyl methyl(2-nitropyridin-3-yl)carbamate (2.2 g, 8.69 mmol) in methanol (30 mL) was added Pd/C (300 mg, 10% purity). The mixture was stirred at 25 °C for 1.5 h under hydrogen (15 psi). The mixture was filtered and the filtrate was concentrated to give tert-butyl (2-aminopyridin-3-yl)(methyl)carbamate (2.1 g, 8.28 mmol, 95.28% yield, 88% purity). MS (ESI) m/z 224.1 $[M+1]^+$.

tert-Butyl (2-isothiocyanatopyridin-3-yl)(methyl)carbamate. To a solution of thiophosgene (1.81 g, 15.77 mmol, 1.21 mL) in dichloromethane (30 mL) was added a mixture of tert-butyl (2-aminopyridin-3-yl)(methyl)carbamate (2 g, 7.88 mmol) in dichloromethane (20 mL) at -5 °C. The mixture was stirred at 5 °C for 1 h. The mixture was diluted with 50 mL of dichloromethane. The organic phase was washed with sodium bicarbonate (20 mL), dried over anhydrous sodium sulfate, filtered and concentrated under vacuum. The residue was purified by silica gel chromatography to give tert-butyl N-(2-isothiocyanato-3-pyridyl)-N-methyl-carbamate (1.8 g, 6.78 mmol, 86.1% yield).

tert-Butyl (2-(3-(imino(5-isopropoxypyridin-2-yl)methyl)thioureido) pyridin-3-yl)(methyl)carbamate. To a solution of tert-butyl (2-isothiocyanatopyridin-3-yl)(methyl)carbamate (1.8 g, 6.78 mmol) and 5-isopropoxypyridine-2-carboxamidine (1.22 g, 6.78 mmol) in dichloromethane (30 mL) and acetone (30 mL) was added triethylamine (2.06 g, 20.35 mmol, 2.83 mL). The mixture was stirred at 25 °C for 3 h under nitrogen. The mixture was concentrated. The residue was poured into water (50 mL). The aqueous phase was extracted with ethyl acetate.The combined organic phase was dried over anhydrous sodium sulfate, filtered and concentrated under vacuum to give tert-butyl N-[2-[(5-isopropoxypyridine-2-

carboximidoyl)carbamothioylamino]-3-pyridyl]-N-methyl-carbamate (3 g, 5.40 mmol, 79.58% yield, 80% purity). MS (ESI) m/z 445.2 $[M+1]^+$.

tert-Butyl (2-((3-(5-isopropoxypyridin-2-yl)-1,2,4-thiadiazol-5-yl)amino) pyridin-3-yl)(methyl)carbamate. To a solution of tert-butyl (2-(3-(imino(5-isopropoxypyridin-2-yl)methyl) thioureido)pyridin-3-yl)(methyl)carbamate (3 g, 5.40 mmol, 1 eq) in ethanol (50 mL) were added iodine (274 mg, 1.08 mmol) and hydrogen peroxide (1.22 g, 10.80 mmol, 1.04 mL, 30% purity). The mixture was stirred at 25 °C for 0.5 h. The reaction was quenched by 30 mL of saturated sodium sulfite. The mixture was concentrated to remove the organic solvent. The mixture was diluted with 100 mL of water, yellow solid formed. The mixture was filtered to give a solid. The solid was triturated with acetonitrile (20 mL) to give tert-butyl (2-((3-(5-isopropoxypyridin-2-yl)-1,2,4-thiadiazol-5-yl) amino)pyridin-3-yl)(methyl)carbamate (1.7 g, 3.69 mmol, 68.31% yield, 96% purity). ^{1}H NMR (400MHz, DMSO-d_6) 12.30 (s, 1H), 8.38 (s, 1H), 8.32 (d, J = 2.7 Hz, 1H), 8.16 (d, J = 8.7 Hz, 1H), 7.79-7.72 (m, 1H), 7.51 (dd, J=2.7, 8.7 Hz, 1H), 7.12 (dd, J_1 = 7.3, J_2 = 5.1 Hz, 1H), 4.81-4.74 (m, 1H), 3.33 (s, 3H), 3.10 (s, 3H), 1.57-1.12 (m, 15H).

N2-(3-(5-isopropoxypyridin-2-yl)-1,2,4-thiadiazol-5-yl)-N3-methylpyridine-2,3-diamine. To a solution of tert-butyl (2-((3-(5-isopropoxypyridin-2-yl)-1,2,4-thiadiazol-5-yl) amino)pyridin-3-yl)(methyl)carbamate (700 mg, 1.52 mmol) in ethyl acetate (15 mL) was added hydrochloride / ethyl acetate (4 M, 21.00 mL) at 0°C. The mixture was stirred at 25°C for 22h. The mixture was filtered and the filter cake was collected to give the crude product. The crude product was triturated with MTBE (30 mL) to give N2-(3-(5-isopropoxypyridin-2-yl)-1,2,4-thiadiazol-5-yl)-N3-methylpyridine-2,3-diamine (433.5 mg, 1.11 mmol, 73.09% yield, 97% purity, HCl). ^{1}H NMR (400MHz, CD_3OD) 8.65 (d, J = 9.2 Hz, 1H), 8.45 (d, J = 2.4 Hz, 1H), 8.24 (dd, J_1 = 9.1, J_2 = 2.5 Hz, 1H), 7.98 (d, J =4.9 Hz, 1H), 7.31 (d, J = 7.8 Hz, 1H), 7.14 (dd, J_1 = 7.8, J_2 = 5.3 Hz, 1H), 5.02-4.97 (m, 1H), 2.99 (s, 3H), 1.47 (d, J = 6.0 Hz, 6H); MS (ESI) m/z 343.1 $[M+1]^+$.

N2-(3-(5-cyclopropoxypyridin-2-yl)-1,2,4-thiadiazol-5-yl)-N3,N3-dimethylpyridine-2,3-diamine (32)

N,N-Dimethyl-2-nitropyridin-3-amine. To a mixture of 3-fluoro-2-nitropyridine (30 g, 211.0 mmol) and potassium carbonate (116.7 g, 8445 mmol) in acetonitrile (500 mL) was added dimethylamine hydrochloride (25.8 g, 317.0 mmol). The reaction mixture was stirred at 20 °C for 2 h. and then concentrated to remove acetonitrile and then diluted with water and ethyl acetate. The aqueous phase was extracted with ethyl acetate and the combined organic phase was dried with anhydrous sodium sulfate, filtered, and concentrated to give N,N-dimethyl-2-nitropyridin-3-amine (34 g, 201.0 mmol, 95.2% yield, 98.8% purity).

N3,N3-Dimethylpyridine-2,3-diamine. To a mixture of N,N-dimethyl-2-nitropyridin-3-amine (30 g, 180 mmol) in methyl alcohol (200 mL) and glycol dimethyl ether (200 mL) was added Pd/C (3 g, 180 mmol, 10% purity) under N_2. The reaction was degassed under N_2 and purged with H_2 three times. The reaction mixture was then stirred at 30°C under H_2 (50 psi) for 5 h. and then filtered, concentrated and purified by silica gel chromatography to give N3, N3-dimethylpyridine-2,3-diamine (24 g, 169.5 mmol, 94.46% yield, 96.9% purity).

2-Isothiocyanato-N,N-dimethylpyridin-3-amine. To mixture of thiophosgene (7.54 g, 65.6 mmol) in dichloromethane (50 mL) was added N3,N3-dimethylpyridine-2,3-diamine (3 g, 21.9 mmol) at 0 °C under N_2. The mixture was stirred at 20 °C for 3 h. The mixture was poured into saturated sodium bicarbonate and extracted with dichloromethane. The combined organic phase was washed with saturated sodium bicarbonate, dried with anhydrous sodium sulfate, filtered and concentrated in vacuum to give 2-isothiocyanato-N,N-dimethylpyridin-3-amine (2.3 g, crude).

N2-(3-(5-Cyclopropoxypyridin-2-yl)-1,2,4-thiadiazol-5-yl)-N3,N3-dimethylpyridine-2,3-diamine. To a mixture of 2-isothiocyanato-N,N-dimethylpyridin-3-amine (1.01 g, 5.64 mmol and diisopropylethylamine (1.46 g, 11.29 mmol) in dichloromethane (20 mL) and acetone (20 mL) was added 5-cyclopropoxypicolinimidamide (1 g, 5.64 mmol) under N_2. The mixture was stirred at 20 °C for 10 h. and then poured into water and extracted with dichloromethane. The combined organic phase was washed with water, dried with anhydrous sodium sulfate, filtered and concentrated. The residue was purified by prep-HPLC to give N2-[3-[5-(cyclopropoxy)-2-pyridyl]-1,2,4-thiadiazol-5-yl]-N3,N3-dimethyl-pyridine-2,3-diamine (277 mg, 0.709 mmol, 12.6% yield, 100% purity, HCl). ^{1}H NMR (400MHz, DMSO-d_6) δ 10.33 (s, 1H), 8.33 (d, J = 2.8 Hz, 1H), 8.07 (d, J = 8.8 Hz, 1H), 7.61 (dd, J_1 = 2.8 Hz, J_2 = 8.8 Hz, 1H), 7.25 (dd, J_1 = 6.8 Hz, J_2 = 8.4 Hz, 1H), 6.92-6.87 (m, 2H), 4.01-3.98 (m, 1H), 2.72 (s, 6H), 0.86-0.72 (m, 2H), 0.72 (m, 2H). MS (ESI): m/z 372.1 [M+1]$^+$.

N2-(3-(5-isopropoxy-4-(trifluoromethyl)pyridin-2-yl)-1,2,4-thiadiazol-5-yl)-N3,N3-dimethylpyridine-2,3-diamine (34)

Methyl 5-chloro-4-(trifluoromethyl)picolinate. To a mixture of 2,5-dichloro-4-(trifluoromethyl)pyridine (5 g, 23.15 mmol) in methanol (100 mL) were added bis(diphenylphosphino)ferrocene]dichloropalladium (846.9 mg, 1.16 mmol) and triethylamine (7.03 g, 69.45 mmol, 9.67 mL) under nitrogen. The mixture was stirred at 60 °C for 3 h under carbon monoxide (50 psi). The mixture was filtered and the filtrate was concentrated. The residue was purified by silica gel chromatography to give methyl 5-chloro-4-(trifluoromethyl)picolinate (4.5 g, 18.78 mmol, 81.14% yield).

5-Chloro-4-(trifluoromethyl)picolinamide. To a mixture of methyl 5-chloro-4-(trifluoromethyl)picolinate (4.5 g, 17.78 mmol) in methanol (100 mL) was added ammonia / methanol (29 M, 45.34 mL) at 0 °C. The mixture was stirred at 15 °C for 16 h. and then concentrated to give 5-chloro-4-(trifluoromethyl)picolinamide (4 g, 17.81 mmol, 94.8% yield).

5-Chloro-4-(trifluoromethyl)picolinonitrile. To a mixture of 5-chloro-4-(trifluoromethyl)picolinamide (4 g, 17.81 mmol) and triethylamine (9.01 g, 148.06 mmol) in dichloromethane (200 mL) was added trifluoroacetic anhydride (18.66 g, 88.84 mmol) at 0 °C. The mixture was stirred at 25 °C for 3 h. The mixture was poured into ice-water (150 mL). The organic phase was separated, and the aqueous phase was extracted with dichloromethane. The combined organic phases were concentrated under vacuum and the residue was purified by silica gel chromatography to give 5-chloro-4-(trifluoromethyl)picolinonitrile (3.6 g, 17.43 mmol, 97.9% yield).

5-Isopropoxy-4-(trifluoromethyl)picolinonitrile. To a mixture of sodium hydride (1.74 g, 43.57 mmol, 60% purity) in DMF (40 mL) was added propan-2-ol (2.09 g, 34.86 mmol) at -20

°C under nitrogen. The mixture was stirred at -10 °C for 0.5 h under nitrogen. Then 5-chloro-4-(trifluoromethyl)picolinonitrile (3.6 g, 17.43 mmol) in DMF (10 mL) was added into the above mixture at -20 °C. The mixture was stirred at -20 °C for 1 h. The mixture was poured into cold saturated ammonium chloride (100 mL) slowly and then diluted with 300 mL of ethyl acetate. The organic phase was washed with brine, dried over anhydrous sodium sulfate, filtered and concentrated under vacuum. The residue was purified by silica gel chromatography to give 5-isopropoxy-4-(trifluoromethyl)picolinonitrile (3 g, 12.38 mmol, 71.0% yield).

5-Isopropoxy-4-(trifluoromethyl)picolinimidamide hydrochloride. Sodium (25 mg, 1.09 mmol) was added into methanol (10 mL) followed by5-isopropoxy-4-(trifluoromethyl)picolinonitrile (0.5 g, 2.17 mmol). The mixture was stirred at 15 °C for 3 h. and then ammonium chloride (174.28 mg, 3.26 mmol) was added and stirring continued at 70 °C for 2 h. The hot mixture was filtered and the filtrate was concentrated to give a residue. The residue was triturated with petroleum ether/ ethyl acetate (2/1, 15 mL) to give 5-isopropoxy-4-(trifluoromethyl)picolinimidamide hydrochloride (0.6 g, crude).

N-((3-(dimethylamino)pyridin-2-yl)carbamothioyl)-5-isopropoxy-4-(trifluoromethyl)picolinimidamide. To a mixture of 2-isothiocyanato-N,N-dimethylpyridin-3-amine (0.38 g, 2.12 mmol) and 5-isopropoxy-4-(trifluoromethyl)picolinimidamide hydrochloride (601 mg, crude) in dichloromethane (15 mL) and acetone (15 mL) was added triethylamine (2.15 g, 21.20 mmol, 2.95 mL). The mixture was stirred at 15 °C for 16 h under nitrogen and then concentrated and the residue was poured into water. The aqueous phase was extracted with ethyl acetate and the combined organic phases were dried over anhydrous sodium sulfate, filtered and concentrated under vacuum to give N-((3-(dimethylamino)pyridin-2-yl) carbamothioyl)-5-isopropoxy-4-(trifluoromethyl)picolinimidamide (1 g, 1.34 mmol, 63.1% yield).

N2-(3-(5-isopropoxy-4-(trifluoromethyl)pyridin-2-yl)-1,2,4-thiadiazol-5-yl)- N3,N3-dimethylpyridine-2,3-diamine. To a mixture of N-((3-(dimethylamino)pyridin-2-yl)carbamothioyl)-5-isopropoxy-4-trifluoromethyl)picolinimidamide (1g, 1.34 mmol, 57% purity) in ethanol (20 mL) were added iodine (67.85 mg, 0.267 mmol) and hydrogen peroxide (454.57 mg, 4.01 mmol, 30% purity) at 0 °C. The mixture was stirred at 15 °C for 0.5 h. The mixture was quenched with saturated sodium sulfite (30 mL). The mixture was concentrated to remove the organic solvent. The aqueous phase was diluted with water (100 mL) and extracted with ethyl acetate. The combined organic phases were dried with anhydrous sodium sulfate, filtered and concentrated. The residue was purified by prep-HPLC (column: Phenomenex Synergi C18 150*30mm*4um;mobile phase: [water(0.225%FA)-ACN];B%: 60%-90%,10min) followed by lyopilization to give N2-(3-(5-isopropoxy-4-(trifluoromethyl)pyridin-2-yl)-1,2,4-thiadiazol-5-yl)-N3,N3-dimethylpyridine-2,3-diamine (431.76 mg, 1.01 mmol, 75.9% yield, 99.77% purity) as a solid; ^{1}H NMR (400MHz, DMSO-d_6) 11.50 (br s, 1H), 8.82 (s, 1H), 8.39 (s, 1H), 8.15 (dd, J_1 =5.0, J_2 =1.2, 1H), 7.64-7.55 (m, 1H), 7.09 (dd, J_1 =7.7, J_2 =4.9, 1H), 5.15-5.08 (m, 1H), 2.68 (s, 6H), 1.37 (d, J = 6.0 Hz, 6H); MS (ESI) m/z 425.1 $[M+1]^+$.

3-(5-Isopropoxy-2-pyridyl)-N-[5-isopropyl-4-(trifluoromethyl)-2-pyridyl]-1,2,4-thiadiazol-5-amine (37)

5-Bromo-4-(trifluoromethyl)pyridin-2-amine. To a mixture of N-bromosuccinimide (45 g, 250 mmol) in THF (350 mL) was added a solution of 4-(trifluoromethyl)pyridin-2-amine (38.7 g,

239 mmol) in THF (200 mL) at 0 °C over a period of 0.5 h. The mixture was stirred at 25 °C for 1 h. The reaction mixture was filtered and the filtrate was concentrated. The residue was purified by column chromatography to give 5-bromo-4-(trifluoromethyl)pyridin-2-amine (34 g, 141 mmol, 59.1% yield) and 5-bromo-4-(trifluoromethyl)pyridin-2-amine (21 g, 66 mmol, 27.7% yield, 76% purity). ^{1}H NMR (400MHz, DMSO-d_6) δ 8.18 (t, J = 3.3 Hz, 1H), 6.86 (s, 1H), 6.73 (br s, 2H), 4.05-3.96 (m, 2H), 1.96 (t, J = 2.9 Hz, 3H), 1.19-1.12 (m, 3H).

5-(Prop-1-en-2-yl)-4-(trifluoromethyl)pyridin-2-amine. To a mixture of 5-bromo-4-(trifluoromethyl)pyridin-2-amine (22 g, 69 mmol), potassium trifluoro(prop-1-en-2-yl)borate (15.40 g, 104 mmol) and cesium carbonate (45 g, 139 mmol) in dioxane (400 mL) and water (40 mL) was added Pd(dppf)Cl_2 (2.5 g, 3.5 mmol) under nitrogen. The mixture was then stirred at 80 °C for 5 h. and then filtered and the filtrate was concentrated. The residue was purified by column chromatography to give 5-isopropenyl-4-(trifluoromethyl)pyridin-2-amine (14 g, crude).

5-Isopropyl-4-(trifluoromethyl)pyridin-2-amine. To a mixture of 5-isopropenyl-4-(trifluoromethyl)pyridin-2-amine (14 g) in methanol (180 mL) was added Pd/C (1 g, 10% purity) and $Pd(OH)_2$ (1 g, 7 mmol) under nitrogen. The reaction mixture was degassed under vacuum and purged with hydrogen for 3 times. The reaction mixture was then stirred at 25 °C under hydrogen for 16 hr. and then filtered and the filtrate was concentrated. The residue was purified by column chromatography to give 5-isopropyl-4-(trifluoromethyl)pyridin-2-amine (7.8 g, 38.2 mmol). ^{1}H NMR (400MHz, $CDCl_3$) δ 8.28-8.16 (m, 1H), 6.72-6.62 (m, 1H), 4.58 (br s, 2H), 3.21-3.10 (m, 1H), 1.27 (d, J = 6.9 Hz, 6H), MS (ESI): m/z 205.1$[M+1]^+$

5-Isopropyl-2-isothiocyanato-4-(trifluoromethyl)pyridine. To a solution of thiophosgene (4.50 g, 39 mmol, 300 mL) in dichloromethane (30 mL) was added 5-isopropyl-4-(trifluoromethyl)pyridin-2-amine (4 g, 19.6 mmol) in dichloromethane (30 mL) at 0 °C. The mixture was stirred at 0 °C for 1 h. and then diluted with sodium bicarbonate (50 mL). The resulting mixture was extracted with ethyl acetate, and the organic phase was concentrated. The residue was purified by column chromatography to give 5-isopropyl-2-isothiocyanato-4-(trifluoromethyl)pyridine (0.7 g, 2.7 mmol, 13.8% yield, 95% purity) and 5-isopropyl-2-isothiocyanato-4-(trifluoromethyl)pyridine (3 g, 8 mmol, 41.1% yield, 66% purity).

1-(5-Isopropoxypyridine-2-carboximidoyl)-3-[5-isopropyl-4-(trifluoromethyl)-2-pyridyl]thiourea. To a solution of 5-isopropyl-2-isothiocyanato-4-(trifluoromethyl)pyridine (0.4 g, 1.6 mmol) and 5-isopropoxypyridine-2-carboxamidine (290 mg, 1.6 mmol) in dichloromethane (10 mL) and acetone (10 mL) was added triethylamine (492 mg, 4.8 mmol). The mixture was stirred at 25 °C for 3 h. and then concentrated to give 1-(5-isop ropoxypyridine-2-carboximidoyl)-3-[5-isopropyl-4-(trifluoromethyl)-2-pyridyl]thiourea (0.6 g, 1.4 mmol, 87.1% yield). MS (ESI): m/z 426.0$[M+1]^+$

3-(5-Isopropoxy-2-pyridyl)-N-[5-isopropyl-4-(trifluoromethyl)-2-pyridyl]-1,2,4-thiadiazol-5-amine. To a mixture of 1-(5-isopropoxypyridine-2-carboximidoyl)-3-[5-isopropyl- 4-(trifluoromethyl)-2-pyridyl]thiourea (0.6 g, 1.4 mmol) in ethanol (10 mL) were added iodine (71.6 mg, 0.282 mmol) and hydrogen peroxide (96 mg, 0.846 mmol, 30% purity). The mixture was stirred at 25 °C for 12 h. and then quenched with saturated sodium sulfite, concentrated, and extracted with ethyl acetate. The organic phase was dried with anhydrous sodium sulfate, filtered and concentrated to give crude product. The crude product was triturated with acetonitrile (5 mL) to give 3-(5-isopropoxy-2-pyridyl)-N-[5-isopropyl-4-(trifluoromethyl)- 2-pyridyl]-1,2,4-

thiadiazol-5-amine (387.83 mg, 0.870 mmol, 61.7% yield, 95% purity). ^{1}H NMR (400MHz, DMSO-*d6*) δ 12.55 (br s, 1H), 8.80 (s, 1H), 8.34 (d, J = 2.8 Hz, 1H), 8.14 (d, J = 8.7 Hz, 1H), 7.51 (dd, J_1 = 8.8, J_2 = 2.9 Hz, 1H), 7.37 (s, 1H), 4.83-4.74 (m, 1H), 3.21-3.10 (m, 1H), 1.32 (d, J = 6.0 Hz, 12H); MS (ESI): m/z 424.1 $[M+1]^+$

3-(5-Isopropoxypyridin-2-yl)-N-isopropyl-N-(3-methylpyridin-2-yl)-1,2,4-thiadiazol-5-amine (43)

N-Isopropyl-3-methylpyridin-2-amine. To a solution of 3-methylpyridin-2-amine (3 g, 27.7 mmol) in THF (60 mL) was added butyl lithium (2.5 M, 13.3 mL) slowly at -70 °C under nitrogen. The mixture was stirred at 0 °C for 30 min. Then 2-iodopropane (5.19 g, 30.5 mmol) in THF (5 mL) was added slowly at 0 °C and the mixture was stirred at 25 °C for 16 h. The mixture was quenched with saturated ammonium chloride (50 mL) at 0 °C and the resulting mixture was extracted with ethyl acetate. The combined organic phase was washed with brine, dried over sodium sulfate, filtered and concentrated at reduced pressure. The residue was purified by silica gel chromatography to give N-isopropyl-3-methyl-pyridin-2-amine (1.3 g, 8.65 mmol, 31.20% yield). ^{1}H NMR (400 MHz, $CDCl_3$) δ 8.02-8.01 (m, 1H), 7.20-7.18 (m, 1H), 6.50-6.47 (m, 1H), 4.32-4.28 (m, 1H), 3.91 (brs, 1H), 2.06 (s, 3H), 1.28 (s, 3H), 1.25 (s, 3H).

O-Phenyl isopropyl(3-methylpyridin-2-yl)carbamothioate. To a mixture of N-isopropyl-3-methyl-pyridin-2-amine (1.20g, 7.9 mmol) and potassium carbonate (3.3 g, 23.9 mmol) in THF (20 mL) was added O-phenyl carbonochloridothioate (2.7 g, 15.9 mmol) which was dissolved with THF (5 mL) slowly at 0°C under nitrogen. The mixture was filtered and the filtrate was concentrated at reduced pressure. The residue was purified by silica gel chromatography to give O-phenyl isopropyl(3-methylpyridin-2-yl)carbamothioate (2.2 g, 7.37 mmol, 92.3% yield, 96% purity).

5-Isopropoxy-N-(isopropyl(3-methylpyridin-2-yl)carbamothioyl) picolin- imidamide and O-phenyl isopropyl(3-methylpyridin-2-yl)carbamothioate. To a mixture of O-phenyl isopropyl(3-methylpyridin-2-yl)carbamothioate (1.5 g, 5.4 mmol) and 5-isopropoxypicolinimidamide (1.15 g, 6.4 mmol) in anhydrous dimethyl sulfoxide (15 mL) was added potassium tert-butoxide (1 M, 6.4 mL) slowly. The mixture was stirred at 25 °C for 16 h. and then poured into water (100 mL). The resulting mixture was extracted with ethyl acetate and the combined organic phase was washed with brine, dried over sodium sulfate, filtered and concentrated at reduced pressure. The residue was purified by silica gel chromatography to give 5-isopropoxy-N-(isopropyl(3-methylpyridin-2-yl)carbamothioyl)picolinimidamide (0.42 g, 1.13 mmol, 21% yield) and and O-phenyl isopropyl(3-methylpyridin-2-yl)carbamothioate (1 g, 3.5 mmol, 65% yield).

3-(5-isopropoxypyridin-2-yl)-N-isopropyl-N-(3-methylpyridin-2-yl)-1,2,4-thiadiazol-5-amine. To a mixture of 5-isopropoxy-N-(isopropyl(3-methylpyridin-2-yl)carbamothioyl) picolinimidamide (0.42 g, 1.13 mmol) and hydrogen peroxide (256 mg, 2.26 mmol, 30% purity) in ethanol (10 mL) was added iodine (57.39 mg, 0.226 mmol). The mixture was stirred at 25 °C for 1 h. The mixture was quenched with saturated sodium sulfite at 0 °C and the resulting mixture was concentrated at reduced pressure. The residue was diluted with water and the aqueous phase was extracted with ethyl acetate, the combined organic phase was washed with brine, dreid over sodium sulfate, filtered and concentrated at reduced pressure to give a residue.

The residue was purified by prep-HPLC (column: Phenomenex Synergi C18 150*30mm*4um;mobile phase: [water(0.225%FA)-ACN];B%: 48%-78%,10.5min) to give 3-(5-isopropoxypyridin-2-yl)-N-isopropyl-N-(3-methylpyridin-2-yl)-1,2,4-thiadiazol-5-amine (254.92 mg, 0.61 mmol, 54% yield, 99% purity, formic acid). ^{1}H NMR (400 MHz, DMSO-d6) δ 8.47 (d, J = 3.2 Hz, 1H), 8.31 (d, J = 2.8 Hz, 1H), 8.17 (s, 1H), 8.04 (d, J = 8.8 Hz, 1H), 7.48-7.45 (m, 2H), 4.79-4.73 (m, 1H), 4.57-4.52 (m, 1H), 2.27 (s, 3H), 1.37 (s, 3H), 1.35 (s, 3H), 1.32 (s, 3H), 1.30 (s, 3H); MS (ESI): m/z 370.1 $[M+1]^+$.

***N*-(2-((3-(5-isopropoxypyridin-2-yl)-1,2,4-thiadiazol-5-yl)amino)pyridin-3-yl)acetamide (46)**

2-Isothiocyanato-3-nitropyridine. To a mixture of thiophosgene (8.27 g, 71.88 mmol, 5.51 mL) in dichloromethane (50 mL) was added a solution of 3-nitropyridin-2-amine (5 g, 35.94 mmol) in dichloromethane (50 mL) at 0 °C under nitrogen. The mixture was stirred at 0 °C for 1 h. and additional thiophosgene (8.27 g, 71.88 mmol, 5.51 mL) was added and stirred at 20 °C for another 16 h. The reaction mixture was filtered and the filtrate was quenched with saturated sodium bicarbonate at 0 °C. The organic phase was separated and dried over anhydrous sodium sulfate, filtered and concentrated under reduced pressure to give a residue. The residue was purified by column chromatography to give 2-isothiocyanato- 3-nitro-pyridine (1.6 g, 8.83 mmol, 24.6% yield). ^{1}H NMR (400MHz, $CDCl_3$) 8.68 (dd, J_1 = 4.8, J_2 = 1.7 Hz, 1H), 8.41 (dd, J_1 = 8.1, J_2 =1.7 Hz, 1H), 7.41 (dd, J_1 = 8.1, J_2 = 4.7 Hz, 1H).

3-(5-Isopropoxypyridin-2-yl)-*N*-(3-nitropyridin-2-yl)-1,2,4-thiadiazol-5-amine. To the mixture 5-isopropoxypicolinimidamide (791.37 mg, 4.42 mmol) and 2-isothiocyanato-3-nitropyridine (800 mg, 4.42 mmol) in dichloromethane (5 mL) and acetone (5 mL) was added triethylamine (1.34 g, 13.26 mmol, 1.84 mL). The mixture was stirred at 20 °C for 2 h. and then concentrated under reduced pressure.The residue was diluted with water , saturated sodium bicarbonate (10 mL) and the resulting mixture was extracted with ethyl acetate. The combined organic phase was dried over sodium sulfate, filtered and concentrated at reduced pressure to give a residue. The residue was purified by column chromatography to give 3-(5-isopropoxypyridin-2-yl)-*N*-(3-nitropyridin-2-yl)-1,2,4- thiadiazol-5 -amine (0.5 g, 1.40 mmol, 31.6% yield). ^{1}H NMR (400MHz, $CDCl_3$) 11.47 (s, 1H), 8.79 (dd, J_1 =4.8, J_2 = 1.6 Hz, 1H), 8.71 (dd, J_1 = 8.3, J_2 = 1.6 Hz, 1H), 8.44 (d, J=2.7 Hz, 1H), 8.28 (d, J = 8.8 Hz, 1H), 7.31 (dd, J_1 =8.7, J_2 =2.9 Hz, 1H), 7.23 (dd, J_1 = 8.3, J_2 = 4.7 Hz, 1H), 4.73-4.66 (m, 1H), 1.41 (d, J = 6.1 Hz, 6H).

***N^2*-(3-(5-isopropoxypyridin-2-yl)-1,2,4-thiadiazol-5-yl)pyridine-2,3-diamine.** To the mixture 3-(5-isopropoxypyridin-2-yl)-*N*-(3-nitropyridin-2-yl)-1,2,4- thiadiazol-5-amine (470 mg, 1.31 mmol) in methanol (30 mL) and THF (15 mL) was added dry palladium on carbon (100 mg, 10% purity) and hydroxide palladium on carbon (100 mg, 20% purity) under nitrogen. The solution was degassed under vacuum and purged with hydrogen several times. The mixture was stirred under hydrogen (15 psi) at 20 °C for 1 hour. The mixture was stirred under hydrogen (15 psi) at 20 °C for another 2 hours. and then under hydrogen (15 psi) at 20 °C for another 2 h. The reaction mixture was filtered and filtrate was concentrated to give a mixture of 3-(5-isopropoxypyridin-2-yl)-*N*-(3-nitropyridin-2-yl)-1,2,4-thiadiazol-5-amine and N^2-(3-(5-isopropoxypyridin-2-yl)-1,2,4-thiadiazol-5-yl)pyridine-2,3-diamine (425 mg crude).

***N^2*-(3-(5-isopropoxypyridin-2-yl)- 1,2,4-thiadiazol-5-yl)pyridine-2,3-diamine.** To a mixture of 3-(5-isopropoxypyridin- 2-yl)-*N*-(3-nitropyridin-2-yl)-1,2,4-thiadiazol-5-amine and N^2-(3-(5-

isopropoxy- pyridin-2-yl)1,2,4-thiadiazol-5-yl)pyridine-2,3-diamine (425 mg, crude) in methanol (30 mL) and THF (15 mL) was added dry palladium on carbon (200 mg, 10% purity) and hydroxide palladium on carbon (200 mg, 20% purity) under nitrogen. The solution was degassed under vacuum and purged with hydrogen several times and stirred under hydrogen (15 psi) at 20 °C for 1 h. The reaction mixture was filtered and filtrate was concentrated. The residue was purified by column chromatography to give N^2-(3-(5-isopropoxypyridin-2-yl)- 1,2,4-thiadiazol-5-yl)pyridine-2,3-diamine (230 mg, 0.7 mmol). ^{1}H NMR (400MHz, DMSO-d_6) 11.61 (s, 1H), 8.32 (d, J = 2.6 Hz, 1H), 8.14 (d, J = 8.7 Hz, 1H), 7.72 (d, J = 4.0 Hz, 1H), 7.50 (dd, J_1 = 8.7, J_2 = 2.7 Hz, 1H), 7.06 (d, J =7.6 Hz, 1H), 6.87 (dd, J_1 = 7.5, J_2 = 5.0 Hz, 1H), 5.55 (s, 2H), 4.79-4.75 (m, 1H), 1.32 (d, J = 5.9 Hz, 6H).

3-Isopropylpyridin-2-amine. To a solution of N^2-(3-(5-isopropoxypyridin-2-yl)-1,2,4-thiadiazol-5-yl)- pyridine-2,3-diamine (210 mg, 0.64 mmol) and acetic anhydride (65.28 mg, 0.64 mmol) in acetonitrile (20 mL) was added triethylamine (647.09 mg, 6.39 mmol, 0.89 mL). The mixture was stirred at 60 °C for 16 h. To the mixture was added acetic anhydride (65.28 mg, 0.64 mmol) and the mixture was stirred at 60 °C for another 16 h. and acetic anhydride (65.28 mg, 0.64 mmol) was added. and stirred at 60 °C for another 5 h. The mixture was concentrated to give crude product which was purified by flash silica gel chromatography (column: Luna C18 150*25 5u;mobile phase: [water(0.225%FA)-ACN];B%: 33%-53%,7.8min) to give 200 mg crude product. The 200 mg of crude product was further purified by prep-TLC to give *N*-[2-[acetyl-[3-(5-isopropoxy-2-pyridyl)-1,2,4-thiadiazol-5-yl] amino]-3-pyridyl]acetamide or *N*-[2-[(Z)-[4-acetyl-3-(5-isopropoxy-2-pyridyl)-1,2,4- thiadiazol-5-ylidene]amino]-3-pyridyl]acetamide (37.5 mg, 0.039 mmol, 6.97% yield, 98% purity) 70.0 mg of crude desired product. The 70.0 mg desired product was purified by prep-HPLC (column: Phenomenex Synergi C18 150*30mm*4um;mobile phase: [water(0.225%FA)-ACN];B%: 35%-59%,10min) to give *N*-[2-[[3-(5-isopropoxy-2-pyridyl)-1,2,4 -thiadiazol-5-yl] amino]-3-pyridyl] acetamide (57.18 mg, 0.150 mmol, 11.8% yield, 98% purity). ^{1}H NMR (400MHz, $CDCl_3$) 9.63 (s, 1H), 8.21 (s, 1H), 8.10-8.04 (m, 2H), 7.74 (s, 1H), 7.16 (dd, J_1 = 8.7, J_2 = 2.4 Hz, 1H), 7.00 (dd, J_1 = 7.3, J_2 = 5.3 Hz, 1H), 4.50-4.47 (m, 1H), 1.91 (s, 3H), 1.29 (d, J = 5.9 Hz, 6H); MS (ESI): m/z 371.3 [M+1]+.

N-(2-((3-(5-isopropoxy-4-(trifluoromethyl)pyridin-2-yl)-1,2,4-thiadiazol-5-yl)amino)pyridin-3-yl)-n-methylacetamide (48)

tert-Butyl methyl(2-nitropyridin-3-yl)carbamate. To a mixture of tert-butyl methyl(2-nitropyridin-3-yl)carbamate (8.5 g, 35.53 mmol) in DMF (100 mL) was added sodium hydride (2.13 g, 53.30 mmol, 60% purity) at 0 °C and the mixture was stirred at 20 °C for 30 min under nitrogen. To the mixture was added iodomethane (5.55 g, 39.08 mmol) at 0°C and stirred at 20 °C for 2 h under nitrogen. The mixture was cooled to 0 °C and poured into ice-water.
The resulting mixture was extracted with ethyl acetate. The combined organic phase was washed with brine, dried with anhydrous sodium sulfate, filtered and concentrated in vacuum. The residue was purified by silica gel chromatography to give tert-butyl methyl(2-nitropyridin-3-yl)carbamate (8.5 g, 33.56 mmol, 94.5% yield). ^{1}H NMR (400MHz, $CDCl_3$) 8.44 (d, J = 3.7 Hz, 1H), 7.83 (d, J = 7.8 Hz, 1H), 7.64 (dd, J_1 = 7.9, J_2 = 4.5 Hz, 1H), 3.33 (s, 3H), 1.55-1.30 (m, 9H).

tert-Butyl (2-aminopyridin-3-yl)(methyl)carbamate. To a solution of tert-butyl methyl(2-nitropyridin-3-yl)carbamate (8.5 g, 33.56 mmol) in methanol (100 mL) were added palladium/carbon (0.4 g, 10 %) and hydroxide palladium /carbon (0.4 g, 20 %). The mixture was stirred at 20 °C for 3 h under hydrogen (15 psi) and then the reaction mixture was filtered and the filtrate was concentrated under vacuum to give tert-butyl (2-aminopyridin-3-yl)(methyl)carbamate (7.3 g, 32.7 mmol, 97.4% yield). ^{1}H NMR (400 MHz, $CDCl_3$) δ7.98 (dd, $J_1 = 5.0, J_2 = 1.5$ Hz, 1H), 7.33 - 7.18 (m, 1H), 6.66 (dd, $J_1 = 7.5, J_2 = 5.0$ Hz, 1H), 4.62 (br s, 2H), 3.13 (s, 3H), 1.40 (br s, 9H).

tert-Butyl (2-isothiocyanatopyridin-3-yl)(methyl)carbamate. To a solution of thiocarbonyl dichloride (3.40 g, 29.56 mmol) in dichloromethane (30 mL) was added a solution of tert-butyl (2-aminopyridin-3-yl)(methyl)carbamate (3.3 g, 14.78 mmol) in dichloromethane (30 mL) dropwise at 0 °C. The mixture was stirred at 0 °C for 1 h. To the mixture was added saturated sodium bicarbonate aqueous and the layers were separated. The aqueous phase was extracted with dichloromethane and the combined organic phases were concentrated under vacuum to give a residue. The residue was purified by silica gel column chromatography to give tert-butyl (2-isothiocyanatopyridin-3-yl)(methyl)carbamate (3 g, 10.85 mmol, 73.4% yield, 96% purity).

tert-Butyl (2-(3-(imino(5-isopropoxy-4-(trifluoromethyl)pyridin-2-yl)methyl)thioureido)pyridin-3-yl)(methyl)carbamate. To a solution of tert-butyl (2-isothiocyanatopyridin-3-yl)(methyl)carbamate (1.2 g, 4.34 mmol) and 5-isopropoxy-4-(trifluoromethyl)picolinimidamide (1.23 g, 4.34 mmol, HCl) in dicholomethane (30 mL) and acetone (30 mL) was added triethylamine (4.39 g, 43.42 mmol). The mixture was stirred at 20 °C for 3 h under nitrogen. The mixture was concentrated under vacuum. The residue was poured into water and the aqueous phase was extracted with ethyl acetate. The combined organic phase was dried over anhydrous sodium sulfate, filtered and concentrated under vacuum to give tert-butyl (2-(3-(imino(5-isopropoxy-4-(trifluoromethyl)pyridin-2-yl)methyl)thioureido)pyridin-3-yl)(methyl)carbamate (2.4 g, crude).

tert-Butyl (2-((3-(5-isopropoxy-4-(trifluoromethyl)pyridin-2-yl)-1,2,4-thiadiazol-5-yl)amino)pyridin-3-yl)(methyl)carbamate. To a solution of tert-butyl (2-(3-(imino(5-isopropoxy-4-(trifluoromethyl)pyridin-2-yl)methyl)thioureido)pyridin-3-yl)(methyl)carbamate (2.4 g, crude) in ethanol (40 mL) were added iodine (237.69 mg, 0.936 mmol) and hydrogen peroxide (1.59 g, 14.05 mmol, 1.35 mL, 30% purity) at 0 °C. The mixture was stirred at 15 °C for 0.5 h. The mixture was quenched with saturated sodium sulfite (50 mL) at 0 °C. The mixture was concentrated to remove the organic solvent. The aqueous phase was diluted with water and extracted with ethyl acetate. The combined organic phase was dried with anhydrous sodium sulfate, filtered and concentrated under vacuum. The residue was purified by silica gel chromatography to give tert-butyl (2-((3-(5-isopropoxy-4-(trifluoromethyl)pyridin-2-yl)-1,2,4-thiadiazol-5-yl)amino)pyridin-3-yl)(methyl)carbamate (2.2 g, 3.96 mmol, 84.7% yield, 92% purity). ^{1}H NMR (400MHz, $CDCl_3$) 8.54 (s, 1H), 8.48 (s, 1H), 8.39 (dd, $J_1 = 5.0, J_2 = 1.5$ Hz, 1H), 7.56 (d, $J = 7.3$ Hz, 1H), 7.07 (dd, $J_1 = 7.7, J_2 = 5.0$ Hz, 1H), 4.95-4.85 (m, 1H), 3.22 (s, 3H), 1.50-1.37 (m, 15H).

tert-Butyl (2-((3-(5-isopropoxy-4-(trifluoromethyl)pyridin-2-yl)-1,2,4-thiadiazol-5-yl)amino)pyridin-3-yl)(methyl)carbamate. To a solution of tert-butyl (2-((3-(5-isopropoxy-4-(trifluoromethyl)pyridin-2-yl)-1,2,4-thiadiazol-5-yl)amino)pyridin-3-yl)(methyl)carbamate (2.2 g, 3.96 mmol) in ethyl acetate (15 mL) was added hydrochloride/ ethyl acetate (4 M, 15 mL) at 0

°C. The mixture was stirred at 25 °C for 2 h. and then concentrated under vacuum to give N2-(3-(5-isopropoxy-4-(trifluoromethyl)pyridin-2-yl)-1,2,4-thiadiazol-5-yl)-N3-methylpyridine-2,3-diamine (1.7 g, 3.69 mmol, 93.1% yield, 97% purity, HCl).

N-(2-((3-(5-Isopropoxy-4-(trifluoromethyl)pyridin-2-yl)-1,2,4-thiadiazol-5-yl)amino)pyridin-3-yl)-N-methylacetamide. To a solution of N2-(3-(5-isopropoxy-4-(trifluoromethyl)pyridin-2-yl)-1,2,4-thiadiazol-5-yl)-N3-methylpyridine-2,3-diamine (0.25 g, 0.543 mmol, HCl, two batches) in acetonitrile (20 mL) were added ethylamine (549.11 mg, 5.43 mmol) and acetic anhydride (66.48 mg, 0.651 mmol). The mixture was stirred at 30 °C for 16 h. The mixture was concentrated under vacuum. The residue was purified by prep-HPLC (column: Phenomenex Synergi C18 150*30mm*4um;mobile phase: [water(0.225%FA)-ACN];B%: 50%-80%,10min) followed by lyophilization to give N-(2-((3-(5-isopropoxy-4-(trifluoromethyl)pyridin-2-yl)-1,2,4-thiadiazol-5-yl)amino)pyridin-3-yl)-N-methylacetamide (129.76 mg, 0.287 mmol, 26.4% yield, 99.9% purity). ^{1}H NMR (400MHz, DMSO-d_6) δ 12.69-11.96 (m, 1H), 8.81 (s, 1H), 8.51-8.35 (m, 2H), 7.93-7.71 (m, 1H), 7.20-7.14 (m, 1H), 5.16-5.06 (m, 1H), 3.28 (s, 1.3H), 3.09 (s, 1.8H), 2.23 (s, 1.1H), 1.70 (s, 1.9H), 1.36 (d, J = 6.0 Hz, 6H); MS (ESI): m/z 453.1 [M+1]$^+$.

N-(6-((3-(5-Isopropoxypyridin-2-yl)-1,2,4-thiadiazol-5-yl)amino)-5-methylpyridin-3-yl)-N-methylacetamide (51)

tert-Butyl (6-bromo-5-methylpyridin-3-yl)carbamate. This reaction was paralleled for two batches: To a solution of 6-bromo-5-methylpyridin- 3-amine (9.5 g, 50.79 mmol) and di-tert-butyl dicarbonate (11.09 g, 50.79 mmol, 11.67 mL) in acetonitrile (120 mL) was added N,N-dimethylpyridin-4-amine (6.21 g, 50.79 mmol) and triethylamine (10.3 g, 101.58 mmol, 14.14 mL). The mixture was stirred at 80 °C for 12 h. Two batches of parallel reactions were combined. The mixture was concentrated and purified by flash silica gel chromatography to give tert-butyl (6-bromo-5-methylpyridin-3-yl)carbamate (22.3 g, crude).

tert-Butyl (6-bromo-5-methylpyridin-3-yl)(methyl)carbamate. This reaction was paralleled for two batches: To a solution of tert-butyl (6-bromo-5-methylpyridin-3-yl)carbamate (7.75 g, crude) in DMF (100 mL) was added sodium hydride (1.51 g, 37.78 mmol, 60% purity) at 0 °C and the mixture was stirred at 25 °C for 0.5 h. Iodomethane (4.98 g, 35.09 mmol, 2.18 mL) was added at 0 °C and the mixture was stirred at 25 °C for 1 h. Two batches of parallel reactions were combined and the mixture was diluted with cold saturated ammonium chloride and extracted with ethyl acetate. The combined organic layers were washed with brine, dried over anhydrous sodium sulfate and filtered. The filtrate was concentrated under reduced pressure and purified by column chromatography to give tert-butyl (6-bromo-5-methylpyridin-3-yl)(methyl)carbamate (20.4 g, crude). MS (ESI): m/z 301.0 [M+1]$^+$.

tert-Butyl (6-((ethoxycarbonyl)amino)-5-methylpyridin-3-yl)(methyl)carbamate. This reaction was paralleled for two batches: A mixture of tert-butyl (6-bromo-5-methylpyridin-3-yl)(methyl)carbamate (2.5 g, 8.30 mmol), ethyl carbamate (1.85 g, 20.75 mmol), sodium 2-methylpropan-2-olate (2 M, 8.30 mL) in THF (50 mL) was degassed and purged with nitrogen 3 times. To the mixture was added [2-(2-aminophenyl)phenyl]-methylsulfonyloxy-palladium;dicyclohexyl-[3,6-dimethoxy-2- (2,4,6-triisopropylphenyl)phenyl]phosphane (376.23 mg, 0.42 mmol) and the mixture was stirred at 100 °C for 12 h under nitrogen atmosphere. Two

batches of parallel reactions were combined and the mixture was diluted with water and extracted with ethyl acetate. The combined organic layers were washed with brine, dried over anhydrous sodium sulfate and filtered. The filtrate was concentrated under reduced pressure and purified by flash silica gel chromatography to give tert-butyl (6-((ethoxycarbonyl)amino)-5-methylpyridin-3-yl)(methyl)carbamate (3.5 g, crude). MS (ESI): m/z 310.1 $[M+1]^+$.

tert-Butyl (6-amino-5-methylpyridin-3-yl)(methyl)carbamate. To a solution of tert-butyl (6-((ethoxycarbonyl)amino)-5-methylpyridin-3-yl)(methyl) carbamate (3.85 g, crude) in ethanol (40 mL) and water (8 mL) was added lithium hydroxide hydrate (2.61 g, 62.23 mmol). The mixture was stirred at 90 °C for 2 h. and then concentrated. The reaction mixture was diluted with water and extracted with ethyl acetate. The combined organic layers were washed with brine, dried over anhydrous sodium sulfate and filtered. The filtrate was concentrated under reduced pressure and purified by flash silica gel chromatography to give tert-butyl (6-amino-5-methylpyridin -3-yl)(methyl)carbamate (1.57 g, 6.62 mmol, 53.2% yield). ^1H NMR (400 MHz, $CDCl_3$) δ 7.83 (s, 1H), 7.24-7.19 (m, 1H), 4.43-4.27 (m, 2H), 3.20 (s, 3H), 2.13 (s, 3H), 1.44 (s, 9H). MS (ESI): m/z 238.1 $[M+1]^+$

tert-Butyl (6-isothiocyanato-5-methylpyridin-3-yl)(methyl)carbamate. To a solution of thiophosgene (1.99 g, 17.32 mmol, 1.33 mL) in dichloromethane (20 mL) was added tert-butyl (6-amino-5-methylpyridin-3-yl)(methyl)carbamate (1.37 g, 5.77 mmol) in dichloromethane (15 mL) at 0 °C. The mixture was stirred at 25 °C for 12 h and then poured into saturated sodium bicarbonate aqueous at 0 °C. The mixture was extracted with dichloromethane, combined organic layers were concentrated under reduced pressure and purified by column chromatography to give tert-butyl (6-isothiocyanato-5-methylpyridin-3-yl)(methyl)carbamate (1.08 g, crude) and recovered tert-butyl (6-amino-5-methylpyridin-3-yl)(methyl)carbamate (170 mg, crude). MS (ESI): m/z 280.1 $M+1]^+$.

tert-Butyl (6-(3-(imino(5-isopropoxypyridin-2-yl)methyl)thioureido)-5-methylpyridin-3-yl)(methyl)carbamate. To a solution of tert-butyl (6-isothiocyanato-5-methylpyridin-3-yl)(methyl)carbamate (857.31 mg, 3.07 mmol) and 5-isopropoxypicolinimidamide (500 mg, 2.79 mmol) in dichloromethane (50mL) and acetone (50 mL) was added triethylamine (1.41 g, 13.95 mmol, 1.94 mL). The mixture was stirred at 25 °C for 12 h and concentrated to give tert-butyl (6-(3-(imino(5-isopropoxypyridin-2-yl)methyl) thioureido)-5-methylpyridin-3-yl)(methyl)carbamate (1.28 g, crude). MS (ESI): m/z 459.2 $[M+1]^+$.

tert-Butyl (6-((3-(5-isopropoxypyridin-2-yl)-1,2,4-thiadiazol-5-yl)amino)-5-methylpyridin-3-yl)(methyl)carbamate. To a solution of tert-butyl (6-(3-(imino(5-isopropoxypyridin-2-yl)methyl) thioureido)-5 -methylpyridin-3-yl)(methyl)carbamate (1.28 g, 2.79 mmol) in ethanol (20 mL) was added iodine (141.69 mg, 0.56 mmol) and hydrogen peroxide (632.96 mg, 5.58 mmol, 0.54 mL, 30% purity) at 0 °C. The mixture was stirred at 25 °C for 40 min and then quenched by addition of saturated sodium sulfite aqueous at 0 °C. The mixture was concentrated and the aqueous phase was extracted with ethyl acetate. The combined organic phase was washed with brine, dried with anhydrous sodium sulfate, filtered and concentrated to give the residue under vacuum. The residue was triturated with petroleum ether : ethyl acetate (10 : 1, 50 mL) for 10 min, then filtered and the filter cake was collected and concentrated under reduced pressure to give tert-butyl (6-((3-(5-isopropoxypyridin-2-yl)-1,2,4-thiadiazol-5-yl) amino)-5-methylpyridin -3-yl)(methyl)carbamate (855 mg, crude). MS (ESI): m/z 457.1 $[M+1]^+$.

N^2-(3-(5-isopropoxypyridin-2-yl)-1,2,4-thiadiazol-5-yl)-N^5,3-dimethylpyridine-2,5-diamine. To a solution of tert-butyl (6-((3-(5-isopropoxypyridin-2-yl)-1,2,4-thiadiazol -5-yl)amino)-5-methylpyridin-3-yl)(methyl)carbamate (805 mg, crude) in ethyl acetate (10 mL) was added hydrogen chloride/ethyl acetate (20 mL,4 M) and the mixture was stirred at 25 °C for 2 h. The mixture was concentrated to give N^2-(3-(5-isopropoxypyridin-2-yl)-1,2,4-thiadiazol-5-yl)-N^5,3-dimethylpyridine-2,5-diamine (692 mg, crude, hydrochloride). MS (ESI): m/z 357.1 $[M+1]^+$.

N-(6-((3-(5-isopropoxypyridin-2-yl)-1,2,4-thiadiazol-5-yl)amino)-5-methylpyridin-3-yl)-N-methylacetamide. To a solution of N^2-(3-(5-isopropoxypyridin-2-yl)-1,2,4-thiadiazol-5-yl)-N^5,3- dimethylpyridine-2,5-diamine (692 mg, 1.76 mmol, hydrochloride) in DMF (15 mL) was added triethylamine (891.10 mg, 8.81 mmol, 1.23 mL) and acetic anhydride (233.74 mg, 2.29 mmol, 0.21 mL). The mixture was stirred at 40 °C for 12 h and then concentrated and purified by prep-HPLC (column: Phenomenex Luna PFP(2) 150*21.2mm 5u;mobile phase: [water(0.2%FA)-ACN];B%: 30%-60%,10min) to give N-(6-((3-(5-isopropoxypyridin-2-yl)-1,2,4-thiadiazol-5-yl)amino)-5-methylpyridin-3- yl)-N-methylacetamide (249.2 mg, 0.621 mmol, 35.3% yield, 99.3% purity). ^{1}H NMR (400 MHz, DMSO-d_6) δ 11.93 (s, 1H), 8.34-8.33 (m, 2H), 8.17 (d, J = 8.8 Hz, 1H), 7.75 (s, 1H), 7.52 (dd, J_1 =8.9, J_2 = 2.9 Hz, 1H), 4.82-4.76 (m, 1H), 3.15 (s, 3H), 2.42 (s, 3H), 1.80 (s, 3H), 1.33 (d, J = 6.0 Hz, 6H). MS (ESI): m/z 399.1 $[M+1]^+$.

2-((3-(5-Isopropoxypyridin-2-yl)-1,2,4-thiadiazol-5-yl)amino)-n,n-dimethyl-5-(trifluoromethyl)nicotinamide (57)

Lithium 2-amino-5-(trifluoromethyl)nicotinate. To a solution of methyl 2-amino-5-(trifluoromethyl)nicotinate (3.8 g, crude) in methanol (40 mL) and water (4 mL) was added lithium hydroxide monohydrate (759.93 mg, 18.11 mmol), and the mixture was stirred at 25 °C for 16 h. The mixture was concentrated under high vacuum to give lithium 2-amino-5-(trifluoromethyl)nicotinate (3.7 g, crude). The crude product was used directly in the next step.

2-Amino-N,N-dimethyl-5-(trifluoromethyl)nicotinamide. To a mixture of lithium 2-amino-5-(trifluoromethyl)nicotinate (3.7 g, 17.45 mmol) and dimethylamine hydrochloride (2.85 g, 34.90 mmol) in DMF (40 mL) was added HATU (8.67 g, 22.79 mmol) and diisopropylethylamine (9.02 g, 69.79 mmol, 12.16 mL) at 0 °C, the mixture was stirred at 25 °C for 16 h. To the mixture was added water (100 mL) and ethyl acetate (50 mL), the aqueous phase was extracted with ethyl acetate, the combined organic phase was washed with brine and concentrated under vacuum to give a residue. The residue was purified by silica gel column chromatography to give 2-amino-N,N-dimethyl-5- (trifluoromethyl)nicotinamide (4.5 g, crude); ^{1}H NMR (400MHz, DMSO-d_6) δ 8.31 (d, J = 1.3 Hz, 1H), 7.65 (d, J = 2.3 Hz, 1H), 6.76 (s, 2H), 2.93-2.89 (m, 6H).

2-Isothiocyanato-N,N-dimethyl-5-(trifluoromethyl)nicotinamide. To a solution of thiophosgene (5.25 g, 45.66 mmol, 3.5 mL) in dichloromethane (40 mL) was added a solution of 2-amino-N,N-dimethyl-5-(trifluoromethyl)nicotinamide (4.5 g, crude) in dichloromethane (20 mL) at 0 °C. The mixture was stirred at 0 °C for 2 h under nitrogen and then saturated sodium bicarbonate was added. The aqueous phase was extracted with dichloromethane and the combined organic phase was concentrated under vacuum to a residue which was purified by silica gel column chromatography to give 2-isothiocyanato-N,N-dimethyl-5-(trifluoromethyl)nicotinamide (1 g, 3.63 mmol, 18.8% yield) and 2-amino-N,N-dimethyl-5-

(trifluoromethyl)nicotinamide (1 g, crude). ^{1}H NMR (400MHz, $CDCl_3$-*d*) δ 8.71 (dd, J_1 = 2.3 Hz, J_2 = 0.7 Hz, 1H), 7.95 (d, J = 2.0 Hz, 1H), 3.18 (s, 3H), 2.95 (s, 3H).

2-(3-(Imino(5-isopropoxypyridin-2-yl)methyl)thioureido)-N,N-dimethyl-5-(trifluoromethyl)nicotinamide. To a solution of 5-isopropoxypicolinimidamide (0.27 g, 1.51 mmol) in dichloromethane (40 mL) and acetone (40 mL) was added triethylamine (2.18 g, 21.55 mmol, 3 mL) and 2-isothiocyanato-N,N-dimethyl-5-(trifluoromethyl) nicotinamide (0.4 g, 1.45 mmol). The mixture was stirred at 25°C for 3 h under nitrogen and then concentrated under vacuum to give 2-(3-(imino(5-isopropoxypyridin-2-yl)methyl)thioureido)-N,N- dimethyl-5-(trifluoromethyl)nicotinamide (0.7 g, crude) which was used directly in the next step.

2-((3-(5-Isopropoxypyridin-2-yl)-1,2,4-thiadiazol-5-yl)amino)-N,N-dimethyl-5-(trifluoromethyl)nicotinamide. To a solution of 2-(3-(imino(5-isopropoxypyridin-2-yl)methyl)thioureido)- N,N-dimethyl-5-(trifluoromethyl)nicotinamide (0.7 g, crude) in ethanol (20 mL) was added hydrogen peroxide (0.4 g, 3.53 mmol, 0.338 mL, 30% purity) and a solution of iodine (90 mg, 0.354 mmol) in ethanol (5 mL) at 0 °C. The mixture was stirred at 25 °C for 2 h and then saturated sodium sulfite was added. and the mixture was concentrated under vacuum. the remaining aqueous phase was extracted with dichloromethane, the combined organic phase was concentrated under vacuum to give a residue. The residue was purified by prep-HPLC (column: Shim-pack C18 150*25*10um;mobile phase: [water(0.225%FA)-ACN];B%: 42%-72%,10min) followed by lyophilization to give 2-((3-(5-isopropoxypyridin-2-yl)-1,2,4-thiadiazol-5-yl)amino)-N,N-dimethyl-5-(trifluoromethyl)nicotinamide (141.3 mg, 0.309 mmol, 20.1% yield, 99.2% purity) as white solid; ^{1}H NMR (400MHz, DMSO-d_6) δ 12.55 (s, 1H), 8.94 (s, 1H), 8.34 (d, J = 2.8 Hz, 1H), 8.21-8.13 (m, 2H), 7.53 (dd, J_1 = 8.7 Hz, J_2 = 2.9 Hz, 1H), 4.83-4.77 (m, 1H), 3.04 (s, 3H), 2.89 (s, 3H), 1.34 (d, J = 6.0 Hz, 6H); MS (ESI): m/z 453.1 $[M+1]^+$.

Experimental Procedures for Synthesis of Compound 1 [1].

1-(5-Methoxypyridin-2-yl)thiourea : A mixture of benzoyl isothiocyanate (3 g, 1.0 mol) and 5-methoxypyridin-2-amine (2.2 g, 1.0 mol) in ethanol (50 mL) under nitrogen atmosphere was stirred at 80 °C for 12 h. After that reaction mixture was cool to 25 °C, poured into ice/water, and stirred for an additional 30 min. The benzoyl thiourea precipitate was collected by filtration and washed with water. The crude material was dissolved in MeOH and treated with 1 N NaOH and the reaction mixture was refluxed for 1 h. After cooling to 25 °C the reaction mixture was poured into ice/water and aqueous 1 N HCl was added to produce pH of 3-4. The reaction was stirred for 30 min and then basified to pH of 8-9 using saturated Na_2CO_3 to give a precipitate that was collected via filtration. The obtained solid was washed with water and dried to give the title compound 1-(5-methoxypyridin-2-yl)thiourea (2.5 g, 74%). LC-MS (ESI): m/z 184.2 $[M+H]^+$.

N-(5-Methoxypyridin-2-yl)-4-(pyridin-2-yl)thiazol-2-amine: To a mixture of 1-(5-methoxypyridin-2-yl)thiourea (2 g, 1.0 mol) and 2-bromo-1-(pyridin-2-yl)ethanone (2.18 g, 1.0 mol) in ethanol (40 mL) under nitrogen atmosphere was stirred at 80 °C for 3 h. The mixture was cool to 25 °C and precipitate was collected by filtration, washed with ethanol, and dried to give the title compounds (1.6 g, 74%). ^{1}H NMR (400 MHz, DMSO-d_6) δ 8.59-8.57 (m, 1H), 8.05-8.04 (m, 1H), 7.99-7.97 (d, 1H), 7.91-7.87 (t, 1H), 7.58 (m, 1H), 7.47-7.44 (dd, 1H), 7.34-7.31 (t, 1H), 7.13-7.11 (d, 1H), 3.81 – 3.79 (s, 3H). LC-MS (ESI): m/z 285.3 $[M+H]^+$.

¹H NMR Spectra for Key Compounds Data S4

Compound 2

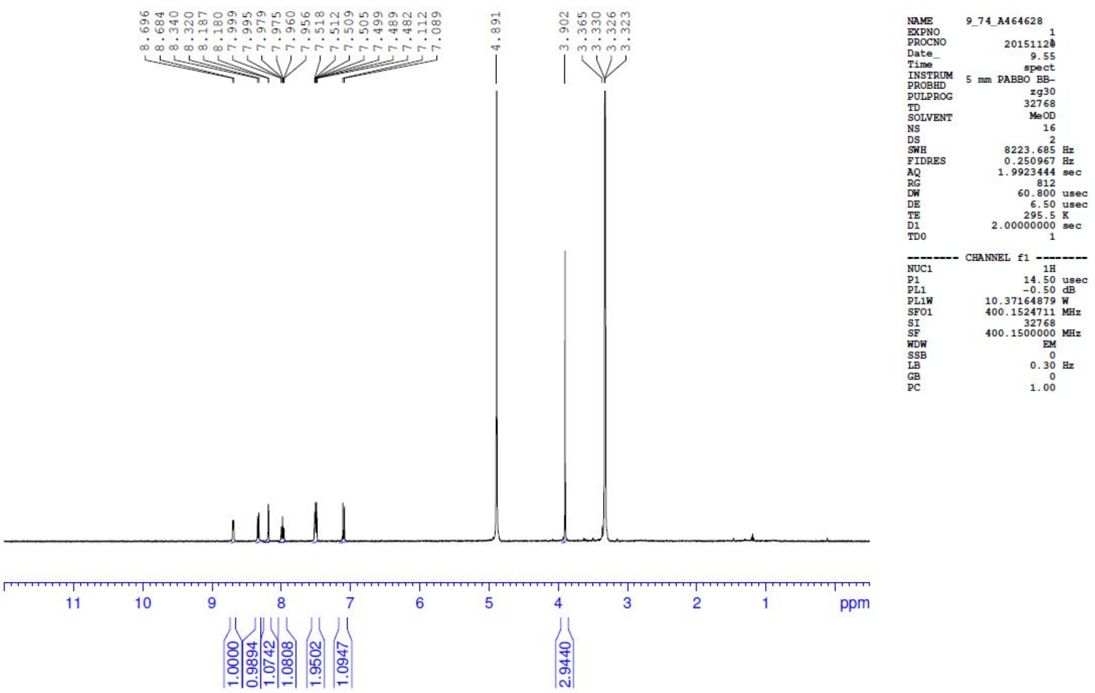

Compound ID: 3

DMSO Bruker_D_400MHz

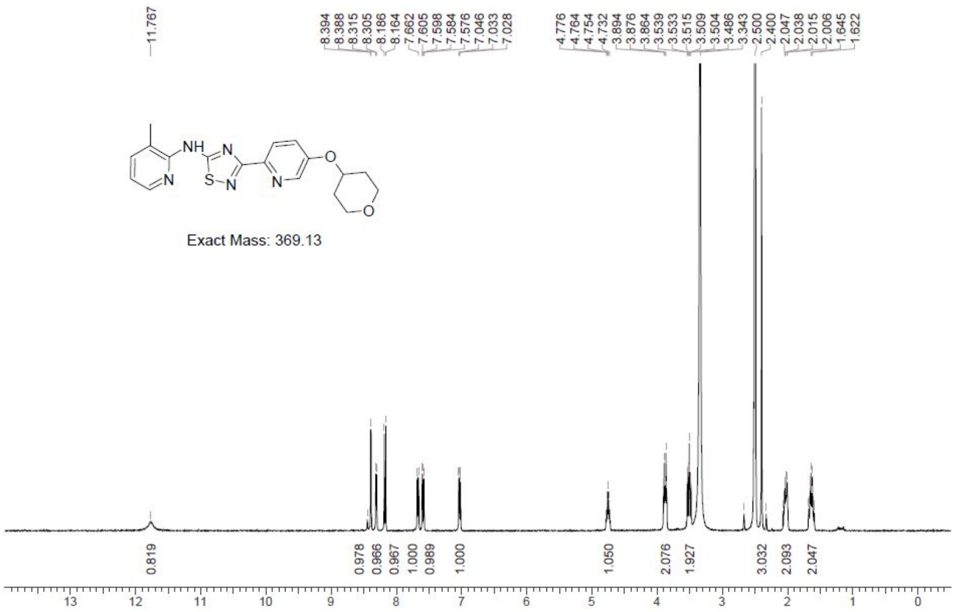

Acquisition Time (sec)	2.9999
Comment	0-P1A DMSO Bruker_D_ 400MHz
Date	20 May 2016 10:28:23
Frequency (MHz)	400.1300
Nucleus	1H
Number of Transients	8
Origin	spect
Original Points Count	24670
Owner	nmrsu
Points Count	65536
Pulse Sequence	zg30
Receiver Gain	117.79
SW(cyclical) (Hz)	8223.68
Solvent	DMSO-d6
Spectrum Offset (Hz)	2467.4072
Spectrum Type	standard
Sweep Width (Hz)	8223.56
Temperature (degree C)	24.045

Operator: Date:

Confidential, for research only not for regulatory filing

Compound ID: 5

DMSO Bruker_E_400MHz

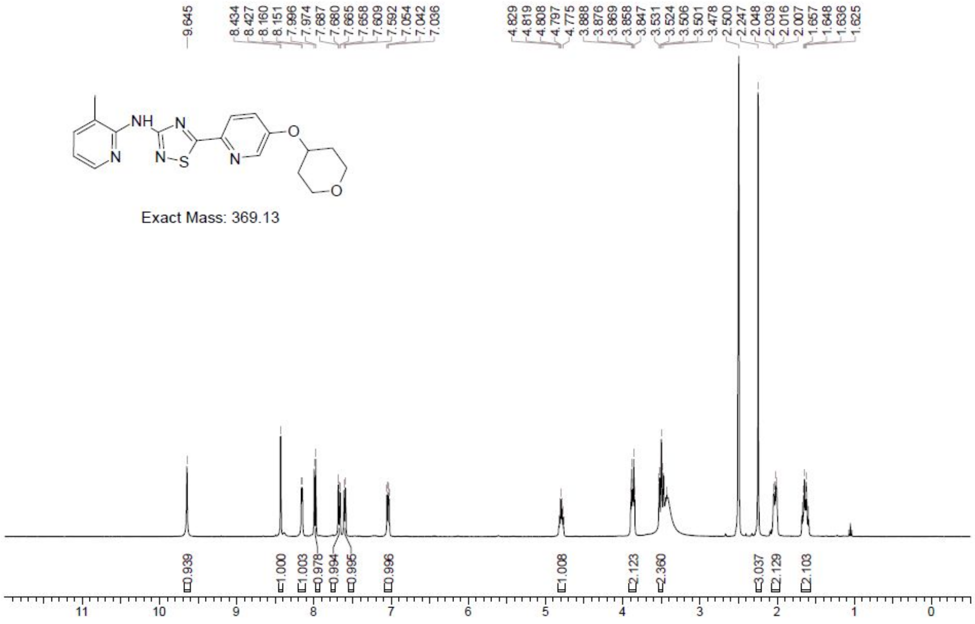

Acquisition Time (sec)	3.0671
Comment	W-A-CRO -201334-6- P1A DMSO Bruker_E_ 400MHz
Date	25 May 2020 10:26:03
Frequency (MHz)	400.1500
Nucleus	1H
Number of Transients	8
Origin	spect
Original Points Count	24576
Owner	nmrsu
Points Count	65536
Pulse Sequence	zg30
Receiver Gain	113.51
SW(cyclical) (Hz)	8012.82
Solvent	DMSO-d6
Spectrum Offset (Hz)	2397.5784
Spectrum Type	standard
Sweep Width (Hz)	8012.70
Temperature (degree C)	25.144

Operator: Date:

Confidential, for research only not for regulatory filing

Compound ID: 6

DMSO Bruker_D_400MHz

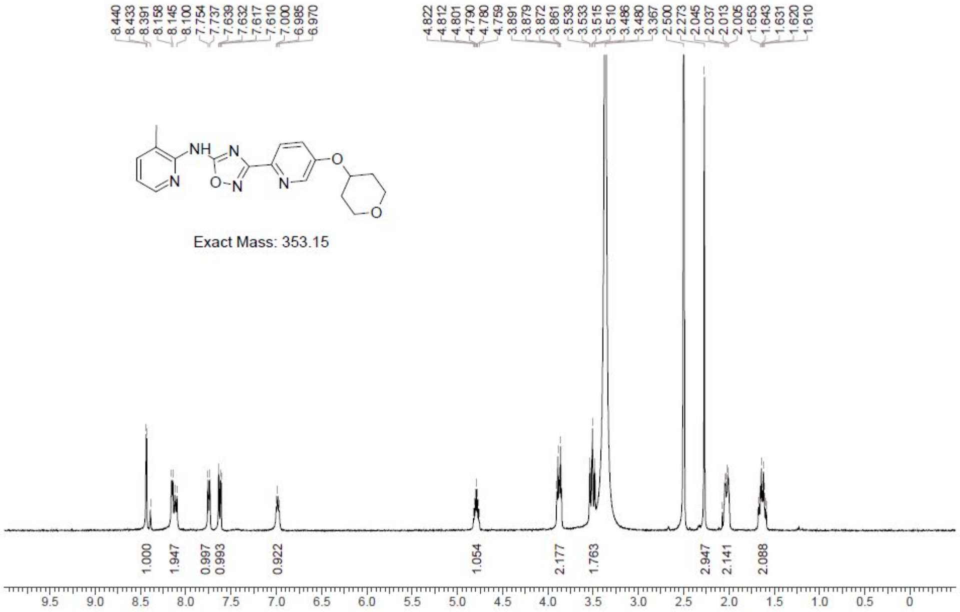

Acquisition Time (sec)	2.9999
Comment	1-P1A DMSO Bruker_D_ 400MHz
Date	27 May 2016 11:09:52
Frequency (MHz)	400.1300
Nucleus	1H
Number of Transients	8
Origin	spect
Original Points Count	24670
Owner	nmrsu
Points Count	65536
Pulse Sequence	zg30
Receiver Gain	125.63
SW(cyclical) (Hz)	8223.68
Solvent	DMSO-d6
Spectrum Offset (Hz)	2467.9272
Spectrum Type	standard
Sweep Width (Hz)	8223.56
Temperature (degree C)	22.851

Operator: Date:

Confidential, for research only not for regulatory filing

Compound ID: 10

DMSO Bruker_A_400MHz

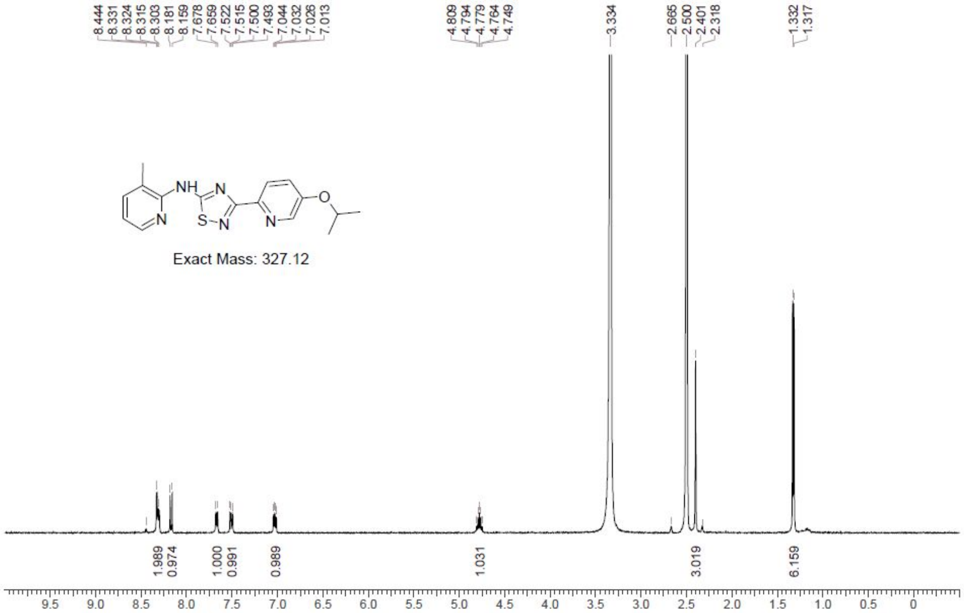

Acquisition Time (sec)	2.9999
Comment	2-P1A1 DMSO Bruker_A_ 400MHz
Date	12 Jun 2016 11:49:12
Frequency (MHz)	400.1300
Nucleus	1H
Number of Transients	8
Origin	spect
Original Points Count	24670
Owner	nmrsu
Points Count	65536
Pulse Sequence	zg30
Receiver Gain	175.36
SW(cyclical) (Hz)	8223.68
Solvent	DMSO-d6
Spectrum Offset (Hz)	2467.2869
Spectrum Type	standard
Sweep Width (Hz)	8223.56
Temperature (degree C)	27.082

Operator: Date:

Confidential, for research only not for regulatory filing

Compound ID: 13

DMSO Bruker_E_400MHz

Exact Mass: 294.07

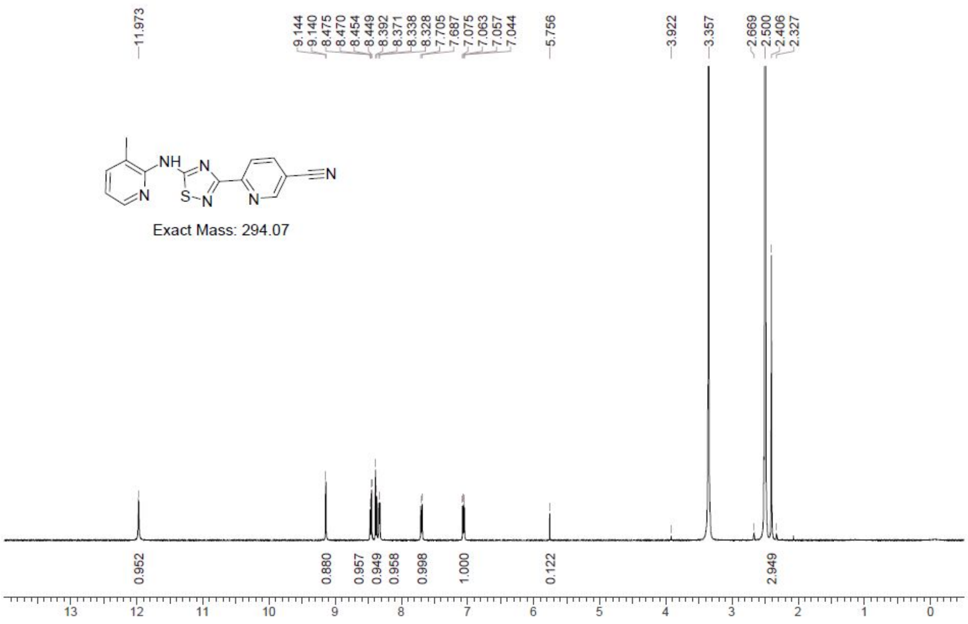

Acquisition Time (sec)	3.0788
Comment	00-P1B DMSO Bruker_E_ 400MHz
Date	09 Oct 2016 10:03:32
Frequency (MHz)	400.1500
Nucleus	1H
Number of Transients	8
Origin	spect
Original Points Count	24670
Owner	nmrsu
Points Count	65536
Pulse Sequence	zg30
Receiver Gain	90.22
SW(cyclical) (Hz)	8012.82
Solvent	DMSO-d6
Spectrum Offset (Hz)	2467.7683
Spectrum Type	standard
Sweep Width (Hz)	8012.70
Temperature (degree C)	20.370

Operator: Date:

Compound ID: 15

DMSO Bruker_F_400MHz

Exact Mass: 352.11

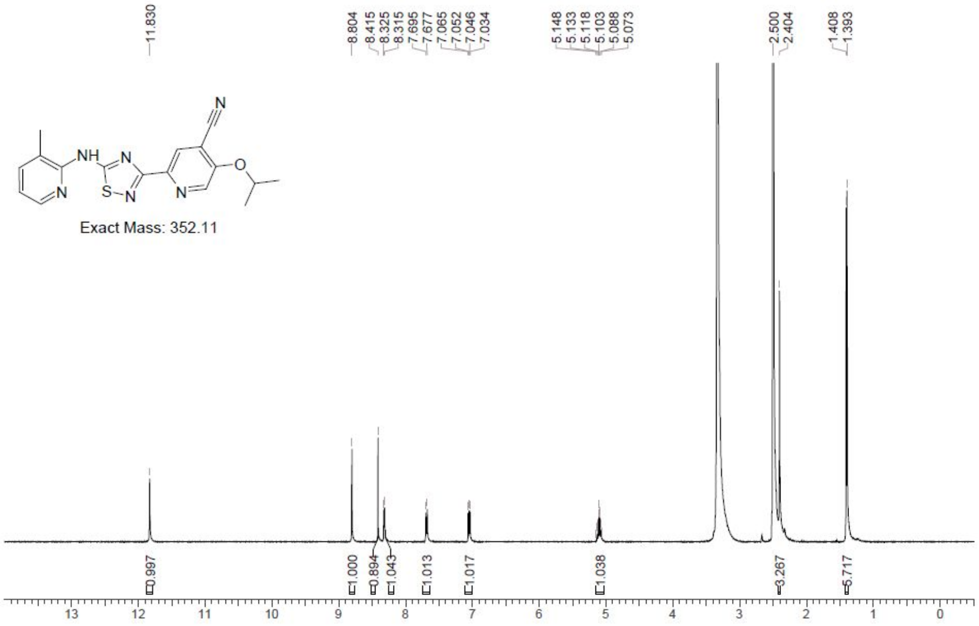

Acquisition Time (sec)	3.0671
Comment	118-P1A DMSO Bruker_F_ 400MHz
Date	24 May 2018 09:32:48
Frequency (MHz)	400.1700
Nucleus	1H
Number of Transients	8
Origin	spect
Original Points Count	24576
Owner	nmrsu
Points Count	65536
Pulse Sequence	zg30
Receiver Gain	73.25
SW(cyclical) (Hz)	8012.82
Solvent	DMSO-d6
Spectrum Offset (Hz)	2397.4988
Spectrum Type	standard
Sweep Width (Hz)	8012.70
Temperature (degree C)	27.137

Operator: Date:

Compound ID: 19

DMSO Bruker_E_400MHz

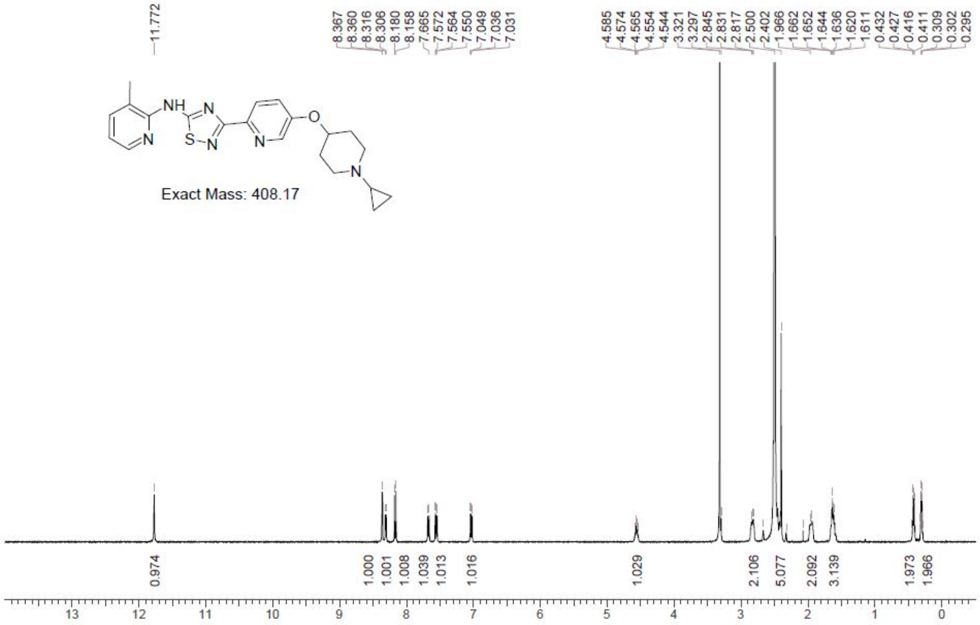

Acquisition Time (sec)	3.0788
Comment	34-P1A3 DMSO Bruker_E_ 400MHz
Date	14 Sep 2016 11:03:05
Frequency (MHz)	400.1500
Nucleus	1H
Number of Transients	8
Origin	spect
Original Points Count	24670
Owner	nmrsu
Points Count	65536
Pulse Sequence	zg30
Receiver Gain	160.91
SW(cyclical) (Hz)	8012.82
Solvent	DMSO-d6
Spectrum Offset (Hz)	2467.6084
Spectrum Type	standard
Sweep Width (Hz)	8012.70
Temperature (degree C)	24.264

Operator: Date:

Compound ID: 21

MeOD Bruker_E_400MHz

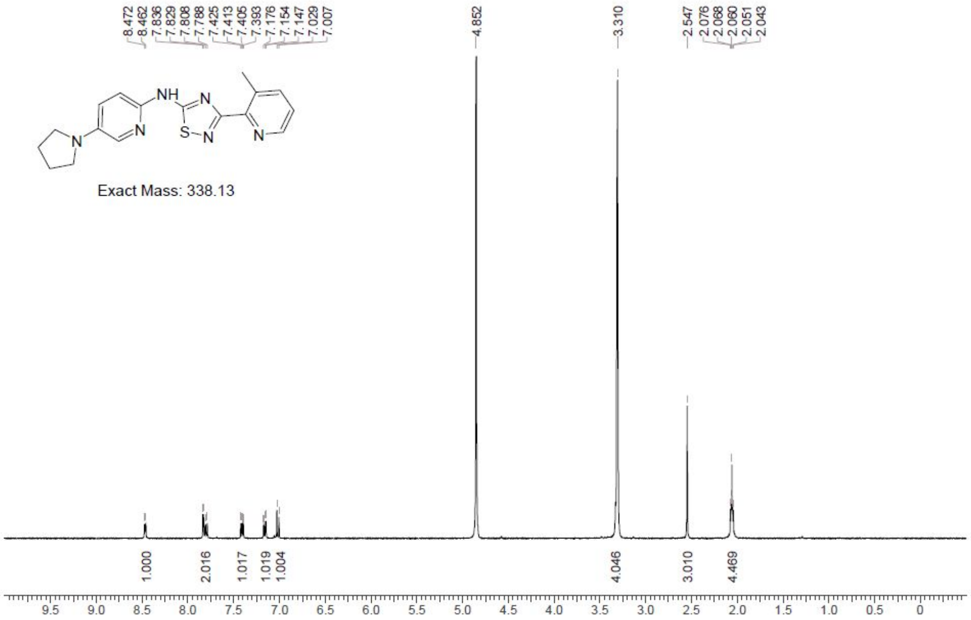

Acquisition Time (sec)	3.0788
Comment	56-P1A2 MeOD Bruker_E_ 400MHz
Date	10 Oct 2016 13:28:51
Frequency (MHz)	400.1500
Nucleus	1H
Number of Transients	8
Origin	spect
Original Points Count	24670
Owner	nmrsu
Points Count	65536
Pulse Sequence	zg30
Receiver Gain	202.23
SW(cyclical) (Hz)	8012.82
Solvent	METHANOL-d4
Spectrum Offset (Hz)	2463.3267
Spectrum Type	standard
Sweep Width (Hz)	8012.70
Temperature (degree C)	25.713

Operator: Date:

Compound ID: 23

DMSO Bruker_D_400MHz

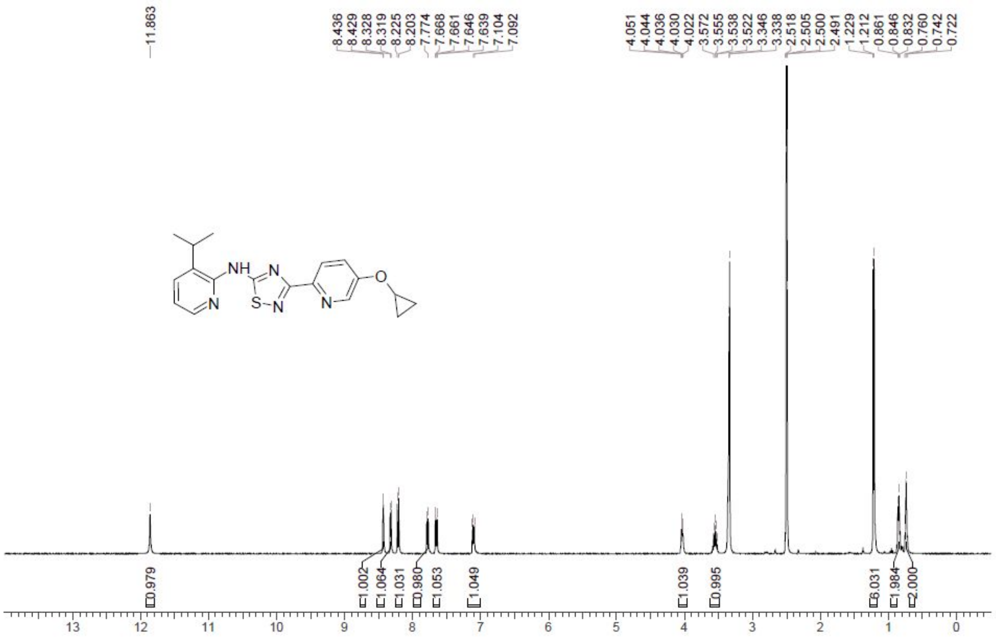

Acquisition Time (sec)	2.9999
Comment	-P1D DMSO Bruker_D_ 400MHz
Date	27 Mar 2017 18:40:45
Frequency (MHz)	400.1300
Nucleus	1H
Number of Transients	8
Origin	spect
Original Points Count	24670
Owner	nmrsu
Points Count	65536
Pulse Sequence	zg30
Receiver Gain	82.86
SW(cyclical) (Hz)	8223.68
Solvent	DMSO-d6
Spectrum Offset (Hz)	2467.8071
Spectrum Type	standard
Sweep Width (Hz)	8223.56
Temperature (degree C)	25.876

Operator: Date:

Confidential, for research only not for regulatory filing

Compound ID: 24

DMSO Bruker_D_400MHz

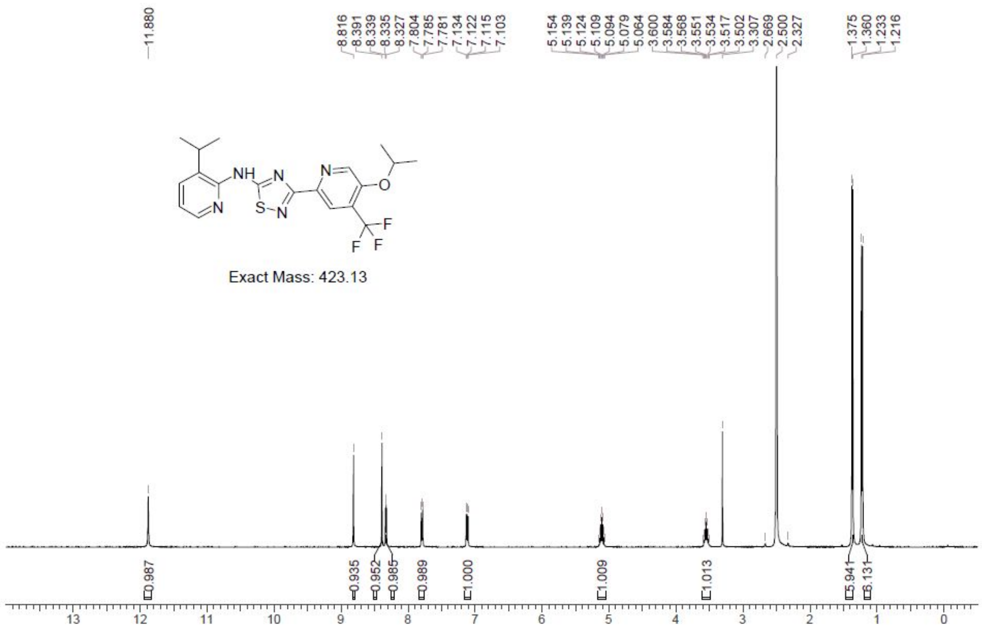

Acquisition Time (sec)	3.0671
Comment	76-P1A DMSO Bruker_D_ 400MHz
Date	19 Jan 2018 11:31:06
Frequency (MHz)	400.1300
Nucleus	1H
Number of Transients	8
Origin	spect
Original Points Count	24576
Owner	nmrsu
Points Count	65536
Pulse Sequence	zg30
Receiver Gain	71.54
SW(cyclical) (Hz)	8012.82
Solvent	DMSO-d6
Spectrum Offset (Hz)	2397.2583
Spectrum Type	standard
Sweep Width (Hz)	8012.70
Temperature (degree C)	29.030

Operator: Date:

Confidential, for research only not for regulatory filing

Compound ID: 25

CDCl3 Bruker_D_400MHz

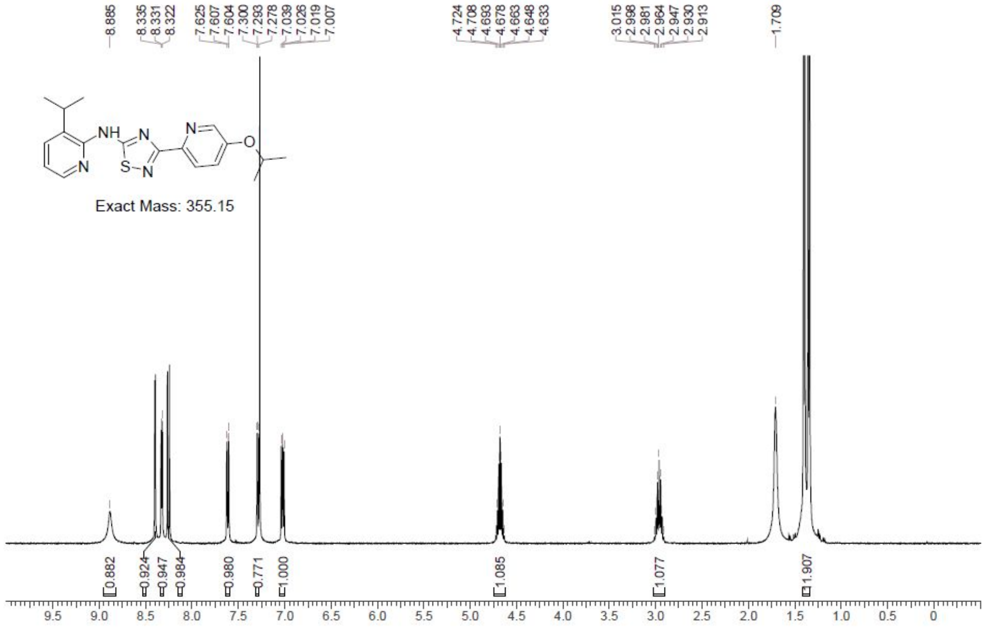

Acquisition Time (sec)	2.9999
Comment	3-P1A CDCl3 Bruker_D_ 400MHz
Date	07 Aug 2017 16:19:41
Frequency (MHz)	400.1300
Nucleus	1H
Number of Transients	8
Origin	spect
Original Points Count	24670
Owner	nmrsu
Points Count	65536
Pulse Sequence	zg30
Receiver Gain	103.63
SW(cyclical) (Hz)	8223.68
Solvent	CHLOROFORM-d
Spectrum Offset (Hz)	2464.9263
Spectrum Type	standard
Sweep Width (Hz)	8223.56
Temperature (degree C)	21.440

Operator: Date:

Compound ID: 30

MeOD Bruker_E_400MHz

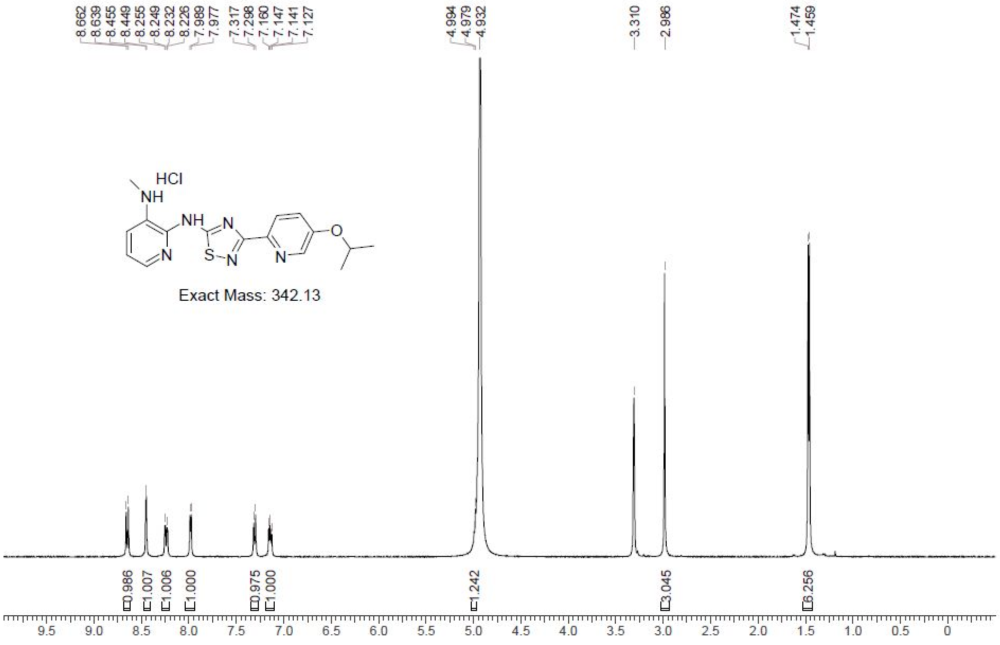

Acquisition Time (sec)	3.0788
Comment	2-P1A MeOD Bruker_E_ 400MHz
Date	13 Jul 2017 10:29:15
Frequency (MHz)	400.1500
Nucleus	1H
Number of Transients	8
Origin	spect
Original Points Count	24670
Owner	nmrsu
Points Count	65536
Pulse Sequence	zg30
Receiver Gain	90.22
SW(cyclical) (Hz)	8012.82
Solvent	METHANOL-d4
Spectrum Offset (Hz)	2463.3267
Spectrum Type	standard
Sweep Width (Hz)	8012.70
Temperature (degree C)	23.718

Operator: Date:

Confidential, for research only not for regulatory filing

Compound ID: 31

MeOD Bruker_E_400MHz

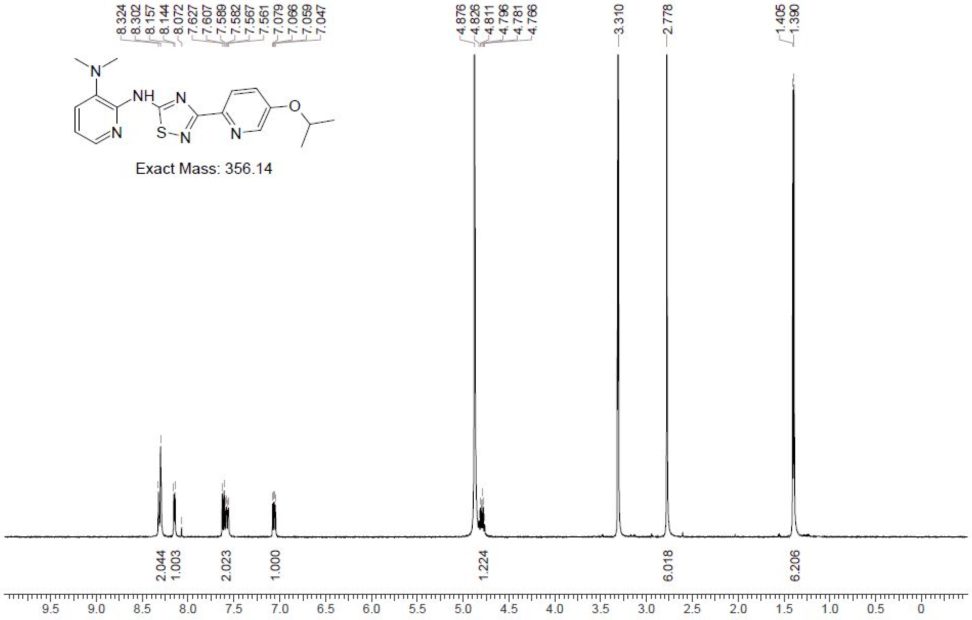

Acquisition Time (sec)	3.0788
Comment	23-P1C MeOD Bruker_E_ 400MHz
Date	10 Nov 2016 11:27:51
Frequency (MHz)	400.1500
Nucleus	1H
Number of Transients	8
Origin	spect
Original Points Count	24670
Owner	nmrsu
Points Count	65536
Pulse Sequence	zg30
Receiver Gain	179.84
SW(cyclical) (Hz)	8012.82
Solvent	METHANOL-d4
Spectrum Offset (Hz)	2463.6067
Spectrum Type	standard
Sweep Width (Hz)	8012.70
Temperature (degree C)	23.170

Operator: Date:

Compound ID: 33

DMSO Bruker_F_400MHz

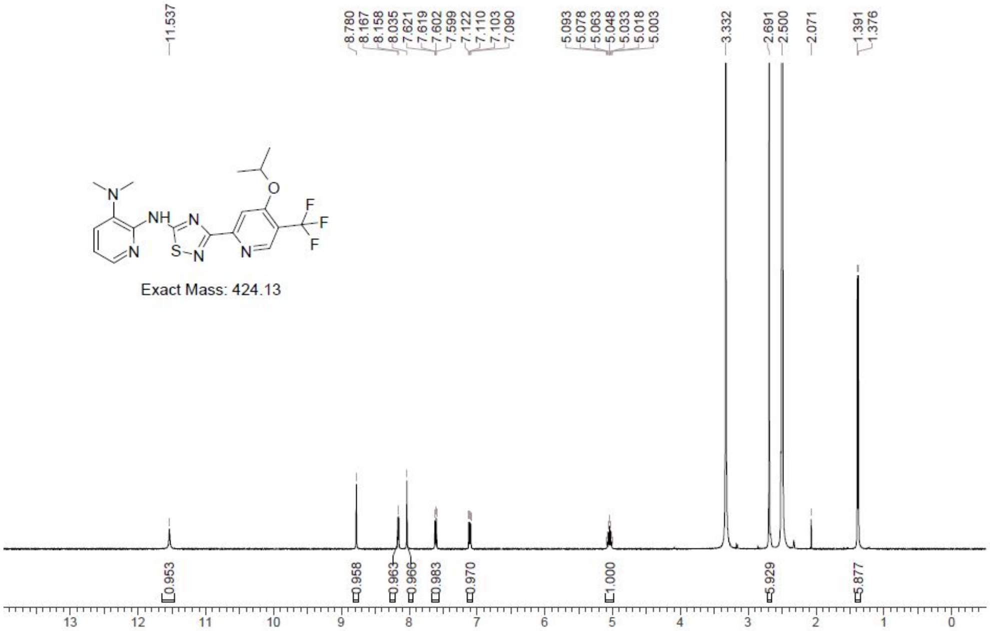

Acquisition Time (sec)	3.0671
Comment	93-P1A DMSO Bruker_F_ 400MHz
Date	15 Nov 2017 10:39:49
Frequency (MHz)	400.1700
Nucleus	1H
Number of Transients	8
Origin	spect
Original Points Count	24576
Owner	nmrsu
Points Count	65536
Pulse Sequence	zg30
Receiver Gain	112.48
SW(cyclical) (Hz)	8012.82
Solvent	DMSO-d6
Spectrum Offset (Hz)	2467.9282
Spectrum Type	standard
Sweep Width (Hz)	8012.70
Temperature (degree C)	25.153

Operator: Date:

Compound ID: 34

DMSO Bruker_F_400MHz

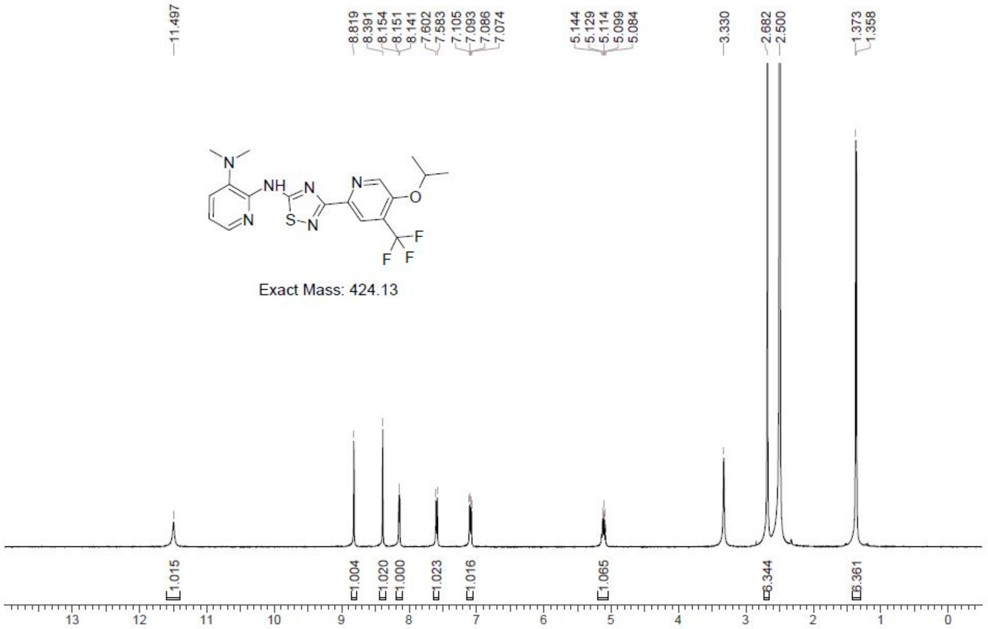

Acquisition Time (sec)	3.0671
Comment	20-P1A DMSO Bruker_F_400MHz
Date	08 Dec 2017 12:08:00
Frequency (MHz)	400.1700
Nucleus	1H
Number of Transients	8
Origin	spect
Original Points Count	24576
Owner	nmrsu
Points Count	65536
Pulse Sequence	zg30
Receiver Gain	90.28
SW(cyclical) (Hz)	8012.82
Solvent	DMSO-d6
Spectrum Offset (Hz)	2398.2190
Spectrum Type	standard
Sweep Width (Hz)	8012.70
Temperature (degree C)	22.569

Operator: Date:

Confidential, for research only not for regulatory filing

Compound ID: 39

DMSO Bruker_E_400MHz

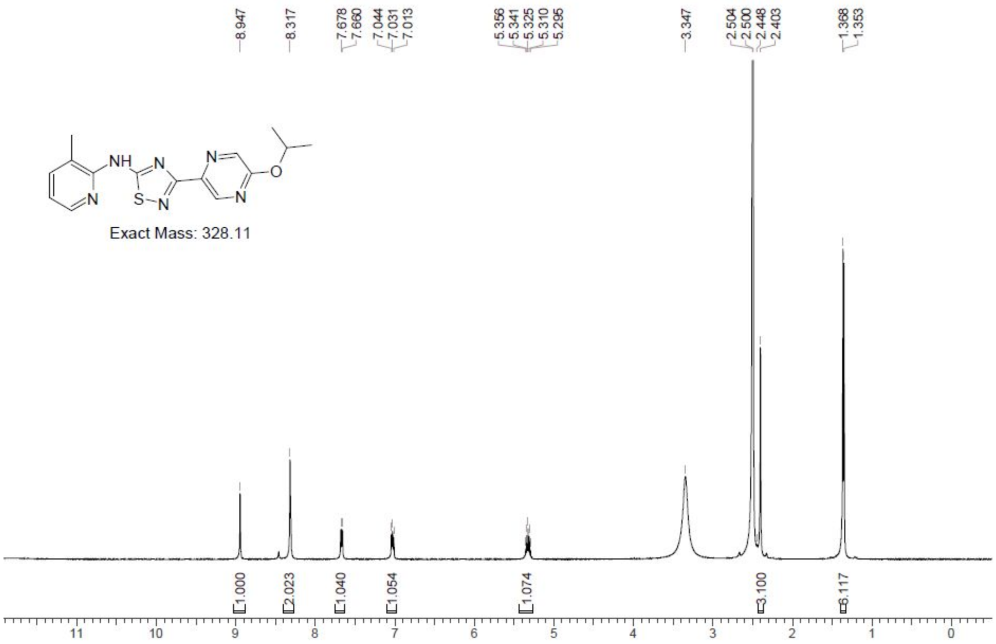

Acquisition Time (sec)	3.0788
Comment	3-P1A DMSO Bruker_E_400MHz
Date	23 Aug 2017 09:09:44
Frequency (MHz)	400.1500
Nucleus	1H
Number of Transients	8
Origin	spect
Original Points Count	24670
Owner	nmrsu
Points Count	65536
Pulse Sequence	zg30
Receiver Gain	90.22
SW(cyclical) (Hz)	8012.82
Solvent	DMSO-d6
Spectrum Offset (Hz)	2467.8884
Spectrum Type	standard
Sweep Width (Hz)	8012.70
Temperature (degree C)	27.147

Operator: Date:

Confidential, for research only not for regulatory filing

Compound ID: 40

DMSO Bruker_D_400MHz

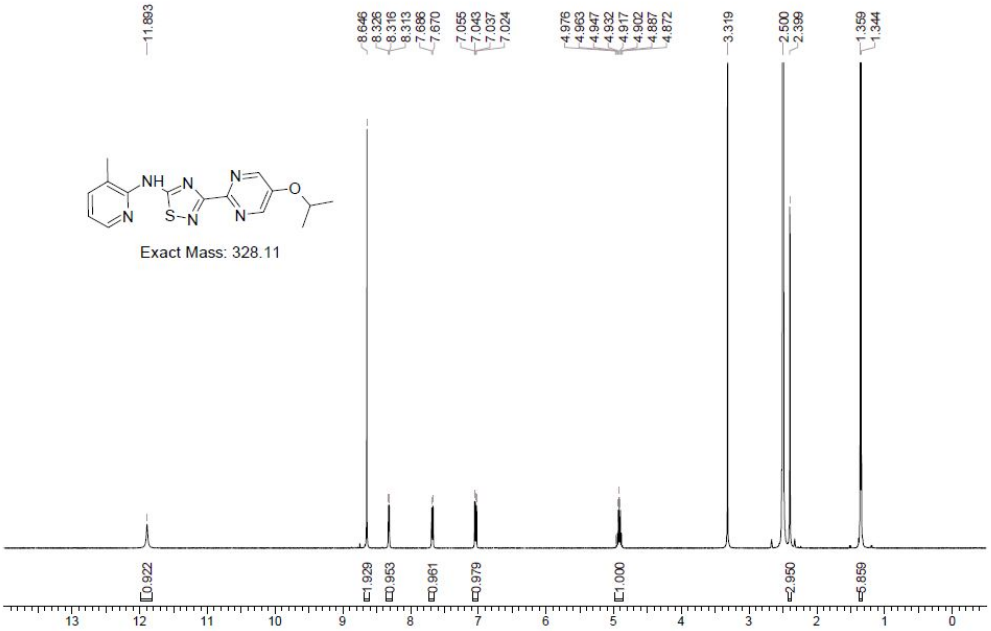

Acquisition Time (sec)	2.9999
Comment	5-P1A DMSO Bruker_D_ 400MHz
Date	08 Apr 2019 10:38:22
Frequency (MHz)	400.1300
Nucleus	1H
Number of Transients	8
Origin	spect
Original Points Count	24670
Owner	nmrsu
Points Count	65536
Pulse Sequence	zg30
Receiver Gain	93.97
SW(cyclical) (Hz)	8223.68
Solvent	DMSO-d6
Spectrum Offset (Hz)	2467.8071
Spectrum Type	standard
Sweep Width (Hz)	8223.56
Temperature (degree C)	25.147

Operator: Date:

Confidential, for research only not for regulatory filing

Compound ID: 43

DMSO Bruker_E_400MHz

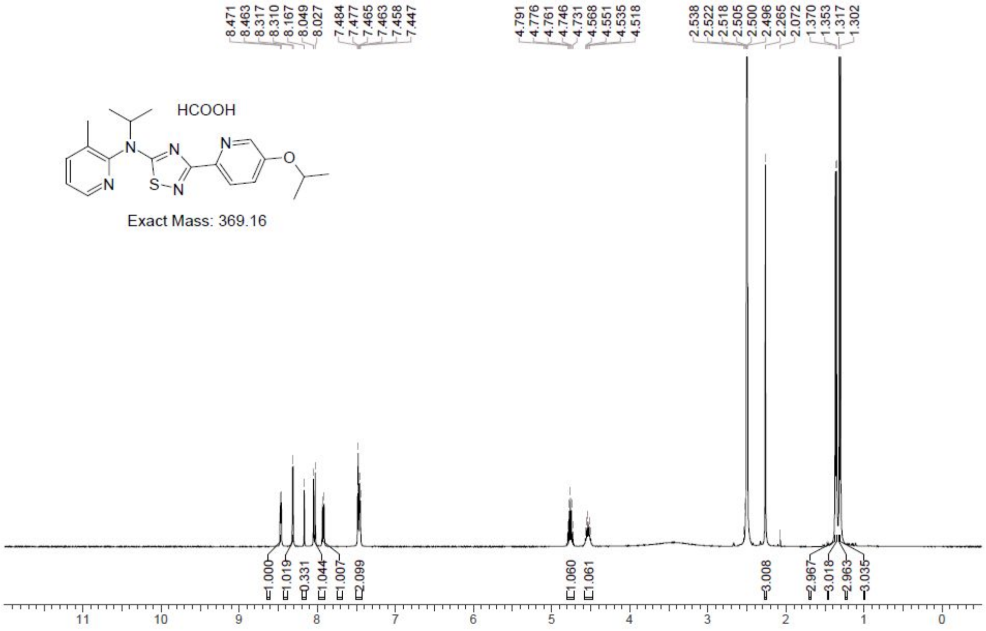

Acquisition Time (sec)	3.0788
Comment	07-P1A DMSO Bruker_E_ 400MHz
Date	18 Sep 2017 09:48:47
Frequency (MHz)	400.1500
Nucleus	1H
Number of Transients	8
Origin	spect
Original Points Count	24670
Owner	nmrsu
Points Count	65536
Pulse Sequence	zg30
Receiver Gain	90.22
SW(cyclical) (Hz)	8012.82
Solvent	DMSO-d6
Spectrum Offset (Hz)	2467.7683
Spectrum Type	standard
Sweep Width (Hz)	8012.70
Temperature (degree C)	26.462

Operator: Date:

Compound ID: 44

MeOD Bruker_F_400MHz

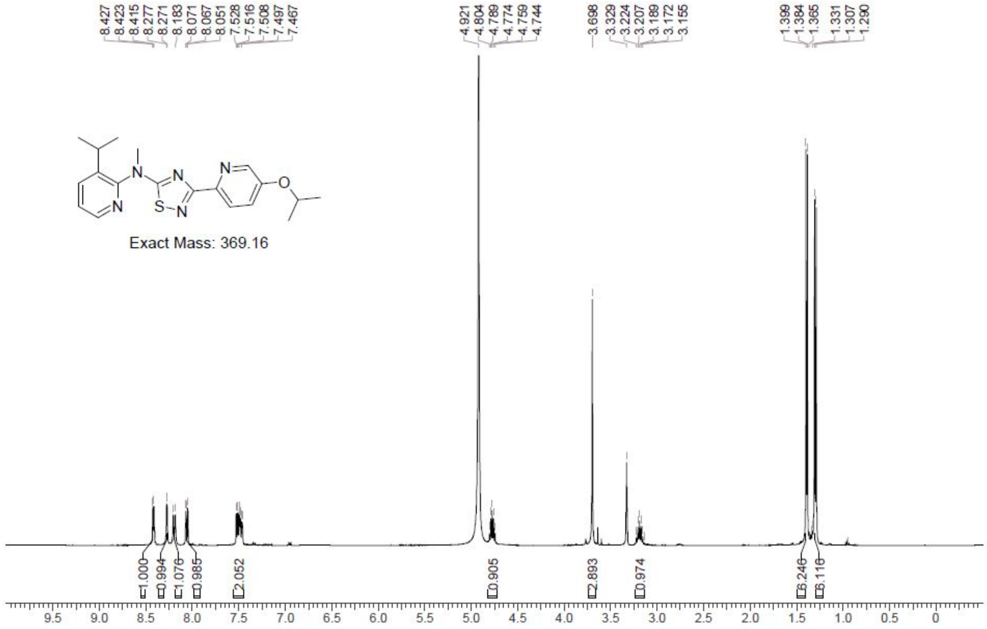

Acquisition Time (sec)	3.0671
Comment	8-P1A MeOD Bruker_F_ 400MHz
Date	13 Sep 2017 11:35:39
Frequency (MHz)	400.1700
Nucleus	1H
Number of Transients	8
Origin	spect
Original Points Count	24576
Owner	nmrsu
Points Count	65536
Pulse Sequence	zg30
Receiver Gain	73.25
SW(cyclical) (Hz)	8012.82
Solvent	METHANOL-d4
Spectrum Offset (Hz)	2471.0496
Spectrum Type	standard
Sweep Width (Hz)	8012.70
Temperature (degree C)	20.971

Operator: Date:

Confidential, for research only not for regulatory filing

Confidential. For research only, Not for regulatory filing.

Compound ID: 47

MeOD Varian_Y_400MHz

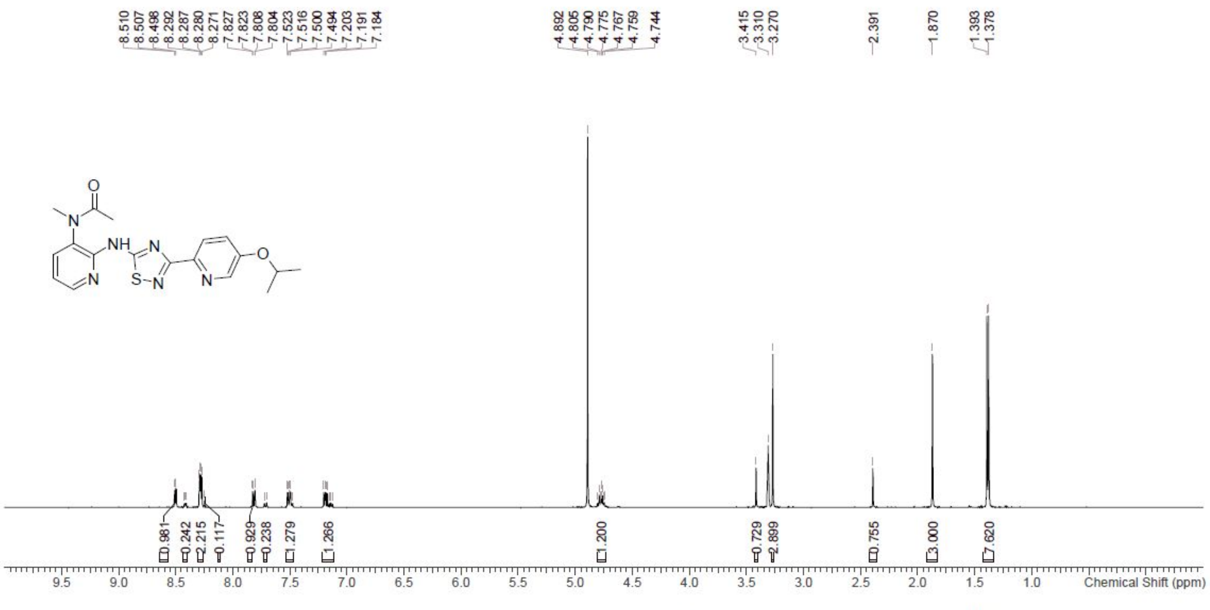

Operator: Date:

Compound ID: 52

DMSO Bruker_F_400MHz

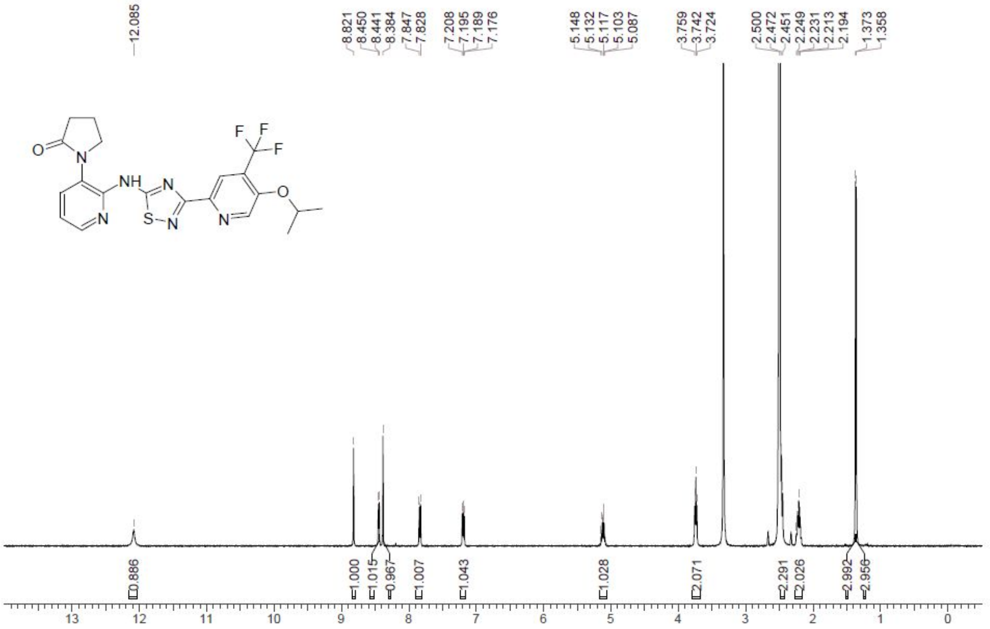

Acquisition Time (sec)	3.0671
Comment	41-P1B DMSO Bruker_F_ 400MHz
Date	04 Apr 2018 11:31:08
Frequency (MHz)	400.1700
Nucleus	1H
Number of Transients	8
Origin	spect
Original Points Count	24576
Owner	nmrsu
Points Count	65536
Pulse Sequence	zg30
Receiver Gain	90.28
SW(cyclical) (Hz)	8012.82
Solvent	DMSO-d6
Spectrum Offset (Hz)	2398.3391
Spectrum Type	standard
Sweep Width (Hz)	8012.70
Temperature (degree C)	22.350

Operator: Date:

Compound ID: 54

DMSO Bruker_E_400MHz

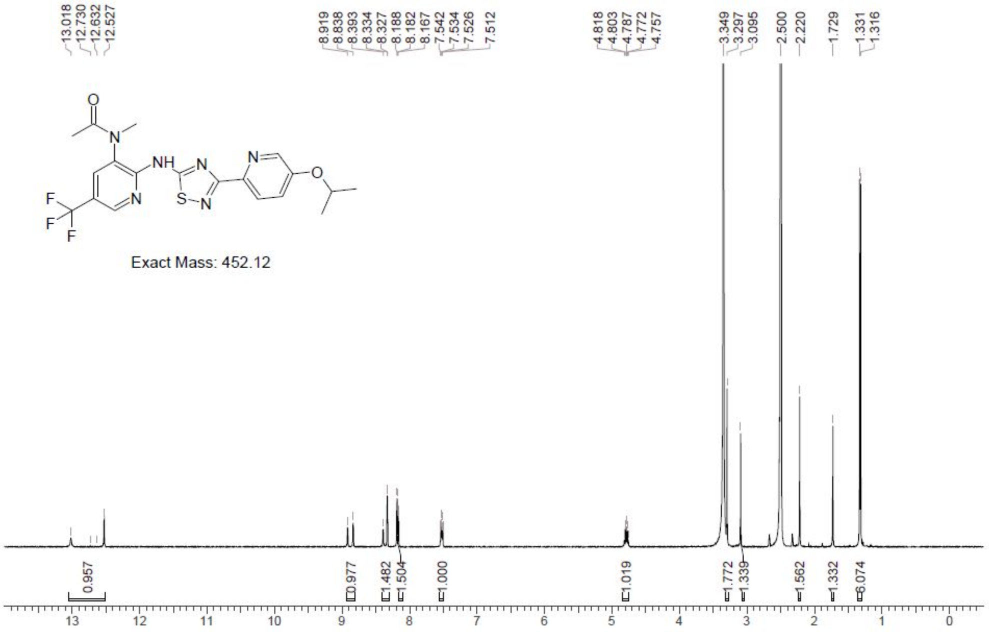

Acquisition Time (sec)	3.0671
Comment	303-P1AA DMSO Bruker_E_ 400MHz
Date	26 Oct 2018 12:51:47
Frequency (MHz)	400.1500
Nucleus	1H
Number of Transients	8
Origin	spect
Original Points Count	24576
Owner	nmrsu
Points Count	65536
Pulse Sequence	zg30
Receiver Gain	90.22
SW(cyclical) (Hz)	8012.82
Solvent	DMSO-d6
Spectrum Offset (Hz)	2398.1787
Spectrum Type	standard
Sweep Width (Hz)	8012.70
Temperature (degree C)	-273.000

Operator: Date:

Confidential, for research only not for regulatory filing

Compound ID: 57

DMSO Bruker_F_400MHz

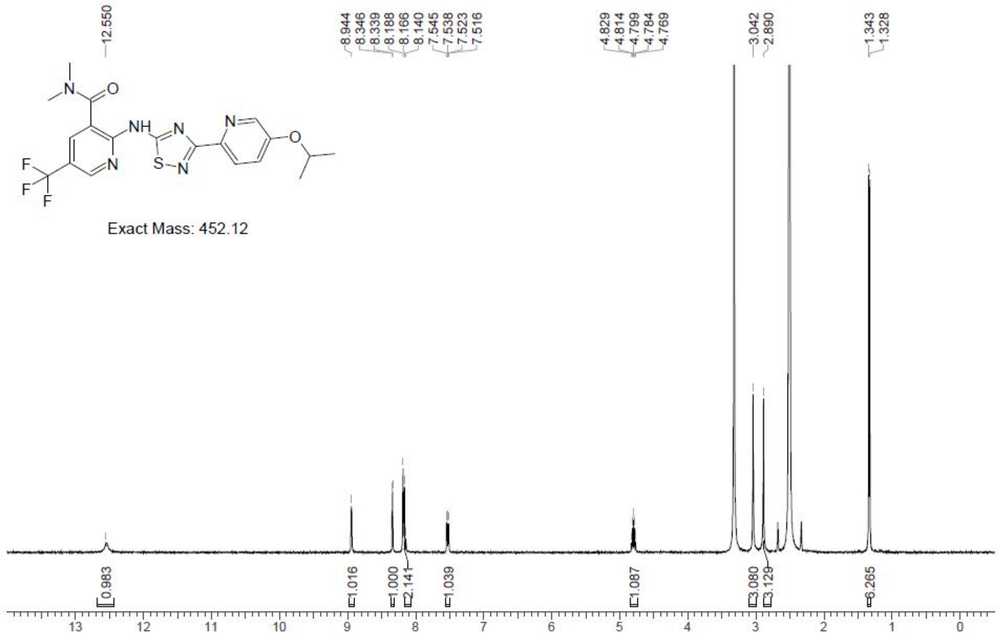

Acquisition Time (sec)	4.0894
Comment	227-P1A DMSO Bruker_F_400MHz
Date	05 Sep 2018 10:25:33
Frequency (MHz)	400.1700
Nucleus	1H
Number of Transients	8
Origin	spect
Original Points Count	32768
Owner	nmrsu
Points Count	65536
Pulse Sequence	zg30
Receiver Gain	90.28
SW(cyclical) (Hz)	8012.82
Solvent	DMSO-d6
Spectrum Offset (Hz)	2401.0203
Spectrum Type	standard
Sweep Width (Hz)	8012.70
Temperature (degree C)	27.180

Operator: Date:

Confidential, for research only not for regulatory filing

Compound ID: 59

DMSO Bruker_D_400MHz

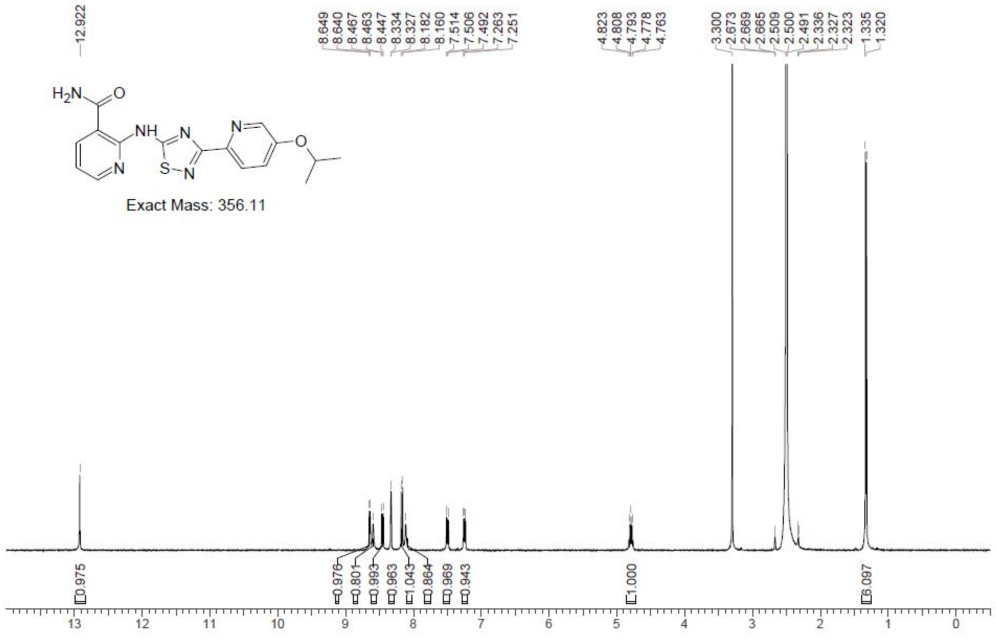

Acquisition Time (sec)	3.0671
Comment	40-P1A DMSO Bruker_D_400MHz
Date	28 Jan 2018 14:19:33
Frequency (MHz)	400.1300
Nucleus	1H
Number of Transients	8
Origin	spect
Original Points Count	24576
Owner	nmrsu
Points Count	65536
Pulse Sequence	zg30
Receiver Gain	125.63
SW(cyclical) (Hz)	8012.82
Solvent	DMSO-d6
Spectrum Offset (Hz)	2397.7786
Spectrum Type	standard
Sweep Width (Hz)	8012.70
Temperature (degree C)	28.687

Operator: Date:

Confidential, for research only not for regulatory filing

Compound ID: 61

DMSO Bruker_D_400MHz

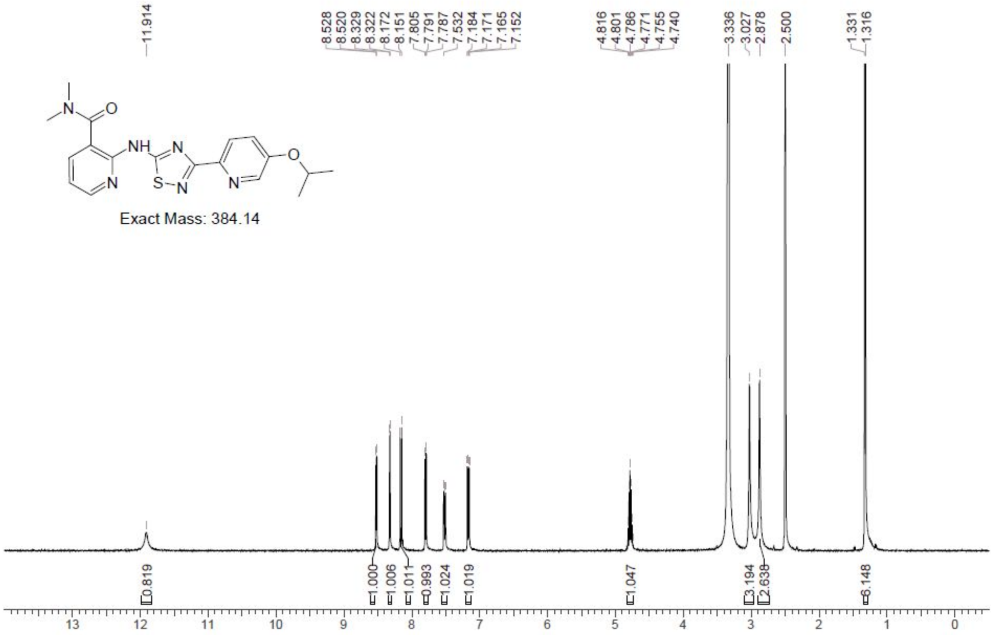

Acquisition Time (sec)	3.0671
Comment	22-P1A DMSO Bruker_D_ 400MHz
Date	23 Apr 2018 11:24:17
Frequency (MHz)	400.1300
Nucleus	1H
Number of Transients	8
Origin	spect
Original Points Count	24576
Owner	nmrsu
Points Count	65536
Pulse Sequence	zg30
Receiver Gain	125.63
SW(cyclical) (Hz)	8012.82
Solvent	DMSO-d6
Spectrum Offset (Hz)	2397.3784
Spectrum Type	standard
Sweep Width (Hz)	8012.70
Temperature (degree C)	27.150

Operator: Date:

Confidential, for research only not for regulatory filing

LCMS Traces for Key Compounds Data S5

Compound 2

```
==========================================================================================
Data file        :  E:\DATA\NOV 15\A463128.D
Vial No.         :  P1-B-09
Injection Date   :  19 -Nov-2015
Injection vol    :  1.000        ul
Sample Name      :  FS-SY15000429-74
Acq Method       :  C:\CHEM32\1\METHODS\PHG_595FAD.M
==========================================================================================
Method info   :A-0.1%HCOOH;B-0.075% HCOOH IN ACN  Flow: 1.0ml/min,
               Column-PHENOMENEX GEMINI NX C18 (50X4.6mm-3μm, )Pos  and Neg  mode
               TIME (MIN)  :  0--0.25     0.25--3.5     3.5--5.25     5.25--5.26     5.26-
               -6.0
                    %B               5             5-95             95               95-5
               5
```

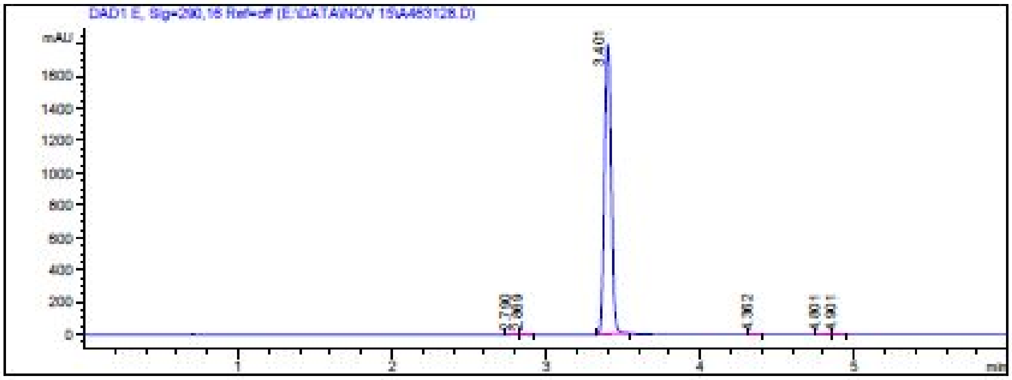

Peak No	RT min	Area	Area %
1	2.790	1.535e+001	0.281
2	2.869	7.601e+000	0.139
3	3.401	5.443e+003	99.469
4	4.362	1.538e+000	0.028
5	4.801	2.490e+000	0.046
6	4.901	2.059e+000	0.038

Compound 3   MW:369.4

```
==============================================
Injection Date    : Thu, 26. May. 2016
Acq Operator      : ADMIN
Location          : P1-F-05
Inj. Vol.         : 3.0   ul
Acq   Method      : D:\METHODS\CLG_A.M
Data Filename     : D:\DATA\160526FG\FGEQ1FE1.D
LCMS-F
```

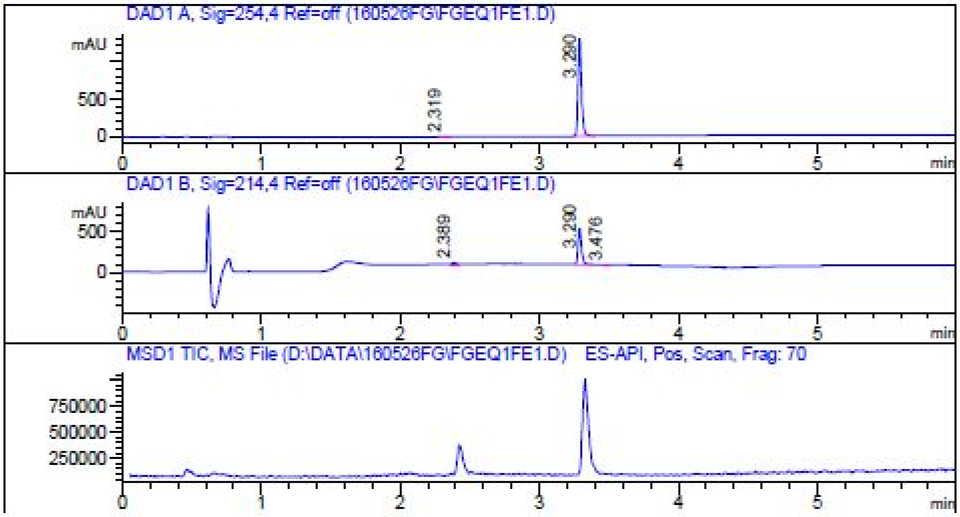

Report

Signal 1 : DAD1 A, Sig=254,4 Ref=off

Peak #	RT [min]	Area	Height	Height %	Width [min]	Area %
1	2.319	2.499	1.435	0.111	0.029	0.109
2	3.290	2286.960	1289.904	99.889	0.027	99.891

Signal 2 : DAD1 B, Sig=214,4 Ref=off

Peak #	RT [min]	Area	Height	Height %	Width [min]	Area %
1	2.389	26.226	16.463	3.558	0.027	3.207
2	3.290	787.527	443.882	95.926	0.030	96.291
3	3.476	4.107	2.387	0.516	0.029	0.502

Compound 6   MW:353.4

==
Injection Date   : Mon, 30. May. 2016
Acq Operator   : ADMIN
Location   : P1-A-07
Inj. Vol.   : 0.8   ul
Acq   Method   : D:\METHODS\CLG_A.M
Data Filename   : D:\DATA\160530FG\FGEU1AG1.D
LCMS-F

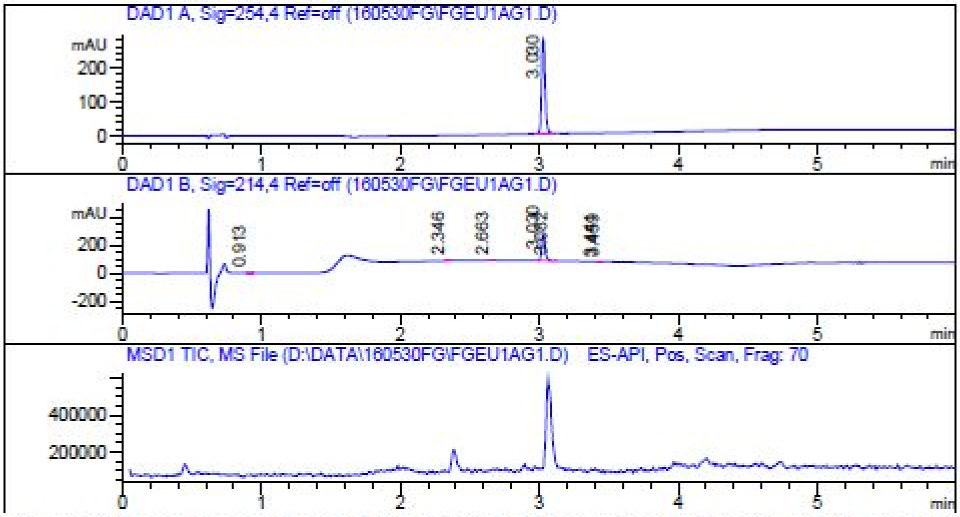

Report

Signal 1 : DAD1 A, Sig=254,4 Ref=off

Peak #	RT [min]	Area	Height	Height %	Width [min]	Area %
1	3.030	470.591	276.397	100.000	0.026	100.000

Signal 2 : DAD1 B, Sig=214,4 Ref=off

Peak #	RT [min]	Area	Height	Height %	Width [min]	Area %
1	0.913	3.108	1.411	0.728	0.037	0.963
2	2.346	4.378	3.410	1.760	0.021	1.357
3	2.663	0.786	0.832	0.429	0.016	0.243
4	3.030	308.168	182.699	94.284	0.026	95.510
5	3.082	3.361	2.732	1.410	0.015	1.042
6	3.441	2.419	1.816	0.937	0.019	0.750
7	3.459	0.434	0.876	0.452	0.008	0.134

Compound 10   MW:327.4

Injection Date   : Mon, 13. Jun. 2016
Acq Operator   : ADMIN
Location   : P1-C-01
Inj. Vol.   : 0.8   ul
Acq   Method   : D:\METHODS\CLG_A.M
Data Filename   : D:\DATA\160613FG\FGFD1CA1.D
LCMS-F

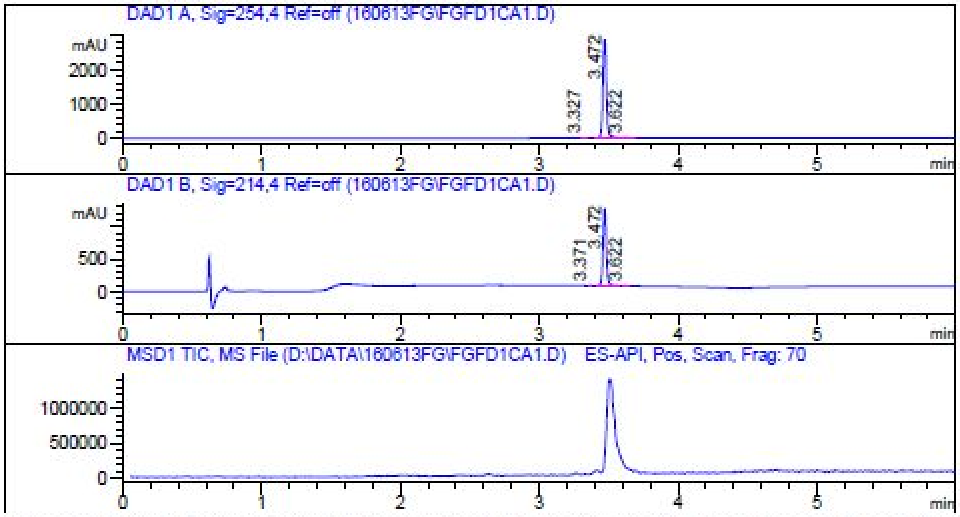

Report

Signal 1 : DAD1 A, Sig=254,4 Ref=off

Peak #	RT [min]	Area	Height	Height %	Width [min]	Area %
1	3.327	1.288	1.009	0.035	0.020	0.026
2	3.472	5007.042	2863.424	99.522	0.027	99.340
3	3.622	31.966	12.733	0.443	0.035	0.634

Signal 2 : DAD1 B, Sig=214,4 Ref=off

Peak #	RT [min]	Area	Height	Height %	Width [min]	Area %
1	3.371	1.392	1.450	0.123	0.016	0.073
2	3.472	1887.006	1175.340	99.462	0.027	99.538
3	3.622	7.359	4.910	0.416	0.025	0.388

Compound 15 MW:352.4

==
Injection Date : Fri, 25- May- 2018 8:52:04 PM
Sample ID : EW11299-118-P1EP
Location : P1-E-07
Inj. Vol. : 1.0 ul
Acq Method : D:\METHODS\CLG_A.M
Data Filename : D:\2018\201805\180525LCMS-F1\LCMS-F1EP1EG1.D
Instrument : CAS-WH-LCMS-F

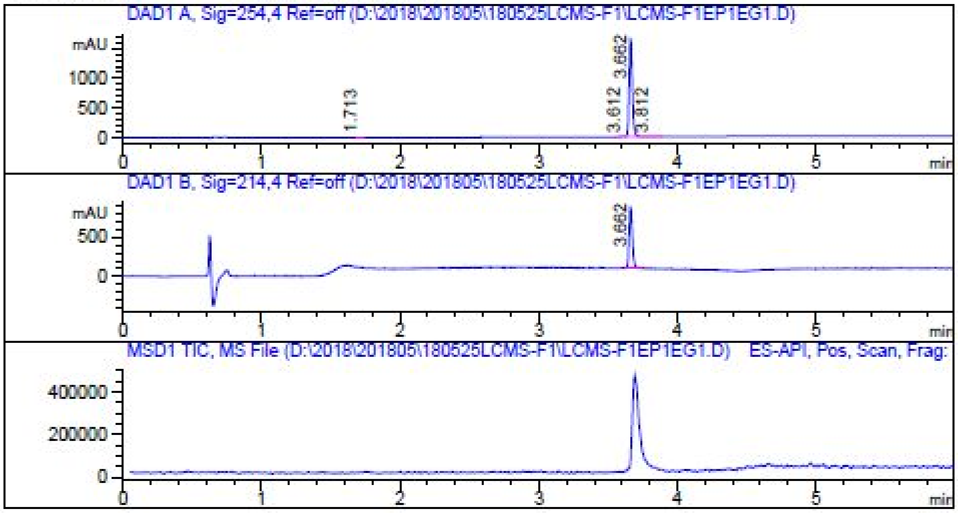

===
Report
===

Signal 1 : DAD1 A, Sig=254,4 Ref=off

Peak #	RT [min]	Height	Height %	Width [min]	Area	Area %
1	1.713	3.096	0.186	0.020	4.0669	0.150
2	3.612	5.158	0.309	0.031	10.0533	0.370
3	3.662	1653.419	99.202	0.025	2690.4185	98.998
4	3.812	5.041	0.302	0.036	13.1109	0.482

Signal 2 : DAD1 B, Sig=214,4 Ref=off

Peak #	RT [min]	Height	Height %	Width [min]	Area	Area %
1	3.662	780.428	100.000	0.026	1308.8074	100.000

Compound 19 MW:408.5

==
Injection Date : Tue, 13. Sep. 2016 11:20:44
Acq Operator : ADMIN
Location : P1-A-09
Inj. Vol. : 3.0 ul
Acq Method : D:\METHODS\CLG_A.M
Data Filename : D:\DATA\160913FG\FGID1AI1.D
LCMS-F

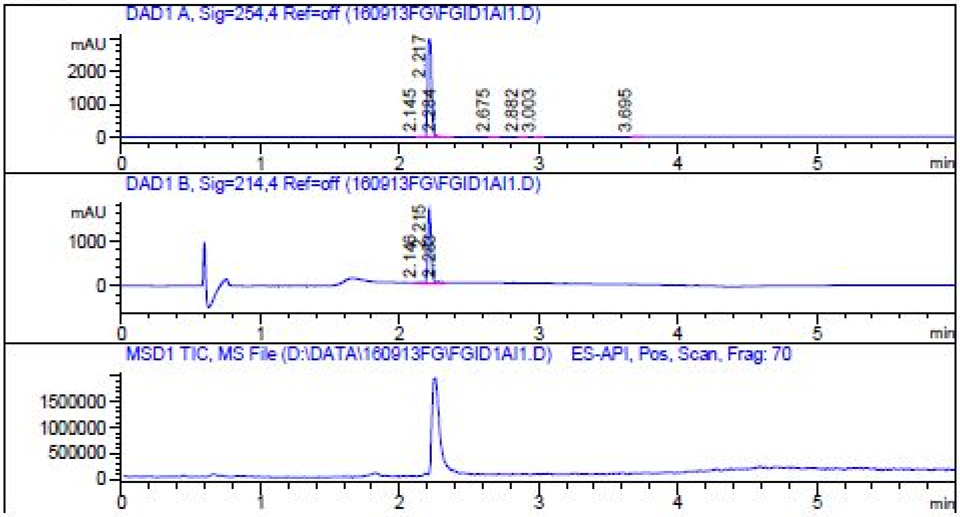

==

Report

==

Signal 1 : DAD1 A, Sig=254,4 Ref=off

Peak #	RT [min]	Area	Height	Height %	Width [min]	Area %
1	2.145	18.545	13.665	0.448	0.021	0.318
2	2.217	5743.161	3004.089	98.458	0.031	98.621
3	2.284	40.007	20.930	0.686	0.026	0.687
4	2.675	2.150	1.246	0.041	0.026	0.037
5	2.882	3.810	1.842	0.060	0.029	0.065
6	3.003	4.435	3.178	0.104	0.022	0.076
7	3.695	11.378	6.200	0.203	0.028	0.195

Signal 2 : DAD1 B, Sig=214,4 Ref=off

Peak #	RT [min]	Area	Height	Height %	Width [min]	Area %
1	2.146	8.509	5.520	0.334	0.024	0.328
2	2.215	2571.619	1639.974	99.108	0.024	99.014
3	2.283	17.098	9.237	0.558	0.026	0.658

Compound 21   MW:338.4

===
Injection Date   : Wed, 28. Sep. 2016   23:01:56
Acq Operator   : ADMIN
Location   : P1-A-09
Inj. Vol.   : 0.5   ul
Acq Method   : D:\METHODS\CLG_A.M
Data Filename   : D:\DATA\160928FG\FGIS1AI2.D
LCMS-F

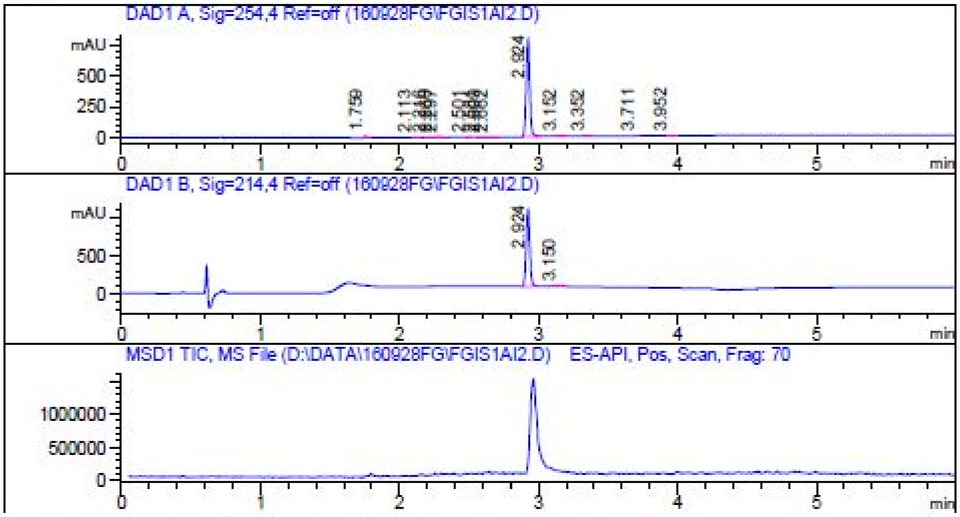

===

Report

===

Signal 1 : DAD1 A, Sig=254,4 Ref=off

Peak #	RT [min]	Area	Height	Height %	Width [min]	Area %
1	1.759	13.888	10.980	1.321	0.019	0.970
2	2.113	2.801	2.265	0.273	0.019	0.196
3	2.216	2.845	1.606	0.193	0.026	0.199
4	2.260	2.462	1.678	0.202	0.022	0.172
5	2.297	2.545	1.557	0.187	0.023	0.178
6	2.501	1.614	1.437	0.173	0.018	0.113
7	2.581	1.816	1.430	0.172	0.020	0.127
8	2.609	2.281	1.623	0.195	0.021	0.159
9	2.662	1.747	1.232	0.148	0.022	0.122
10	2.924	1389.359	801.676	96.471	0.026	97.055
11	3.152	2.746	1.584	0.191	0.026	0.192
12	3.352	2.448	1.068	0.129	0.038	0.171
13	3.711	1.375	0.813	0.098	0.028	0.096
14	3.952	3.582	2.054	0.247	0.025	0.250

Signal 2 : DAD1 B, Sig=214,4 Ref=off

Compound 23 MW:353.4

==

Injection Date : Tue, 28- Mar- 2017 15:20:26

Acq Operator : ADMIN

Location : P1-B-08

Inj. Vol. : 3.0ul

Acq Method : D:\METHODS\CLG_A.M

Data Filename : D:\DATA\170328LCMS-F1\LCMS-F1CS1BH1.D

Instrument : F

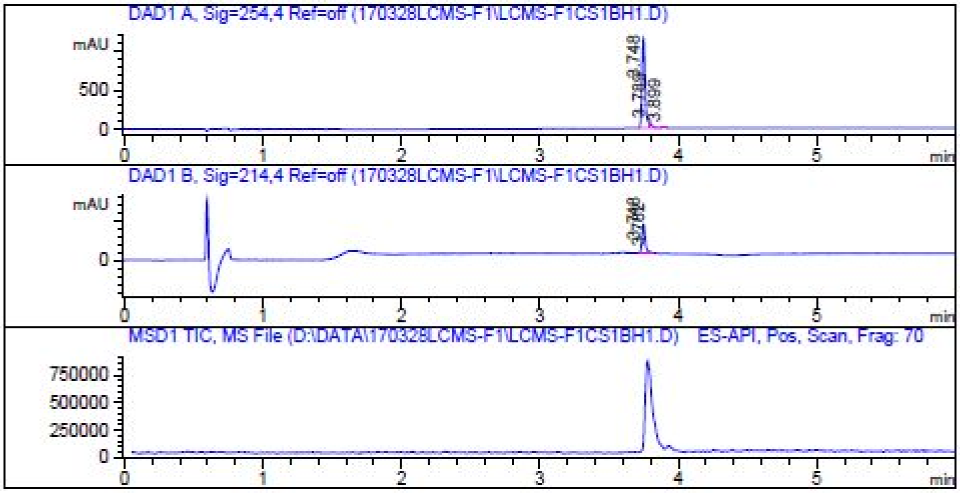

===

Report

===

Signal 1 : DAD1 A, Sig=254,4 Ref=off

Peak #	RT [min]	Height	Height %	Width [min]	Area	Area %
1	3.748	1146.553	93.670	0.027	1842.049	95.415
2	3.789	71.154	5.813	0.019	79.834	4.135
3	3.899	6.332	0.517	0.023	8.680	0.450

Signal 2 : DAD1 B, Sig=214,4 Ref=off

Peak #	RT [min]	Height	Height %	Width [min]	Area	Area %
1	3.748	362.011	94.253	0.026	557.693	96.183
2	3.782	22.075	5.747	0.017	22.129	3.817

Compound 24 MW:423.5

===

Injection Date	: Fri, 19- Jan- 2018	4:02:27 PM
Sample ID	: EW8002-276-P1AJ	
Location	: P1-B-07	
Inj. Vol.	: 1.0 ul	
Acq Method	: D:\METHODS\CLG_A.M	
Data Filename	: D:\2018\201801\180119LCMS-F1\LCMS-F1AJ1BG1.D	
Instrument	: LCMS-F	

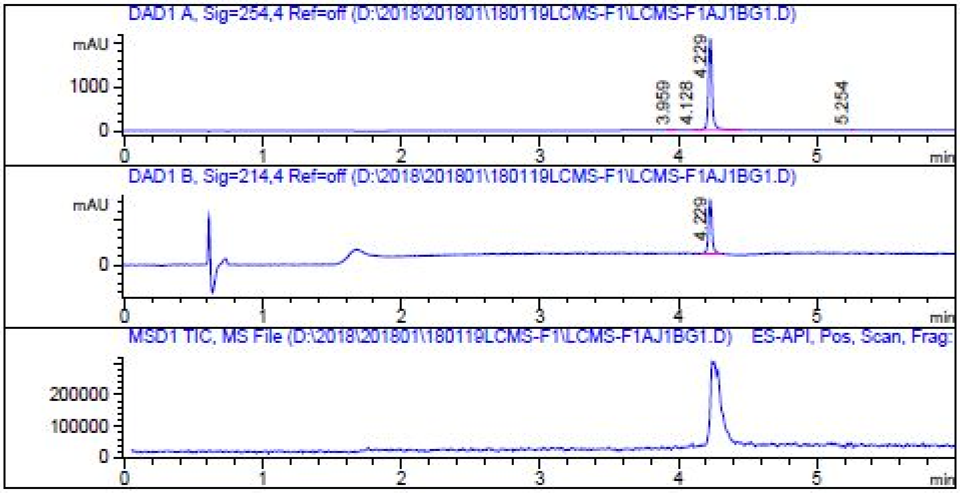

===

Report

===

Signal 1 : DAD1 A, Sig=254,4 Ref=off

Peak #	RT [min]	Height	Height %	Width [min]	Area	Area %
1	3.959	2.625	0.125	0.019	3.424	0.087
2	4.128	8.802	0.419	0.025	15.102	0.384
3	4.229	2089.396	99.429	0.028	3913.128	99.519
4	5.254	0.572	0.027	0.010	0.382	0.010

Signal 2 : DAD1 B, Sig=214,4 Ref=off

Peak #	RT [min]	Height	Height %	Width [min]	Area	Area %
1	4.229	589.182	100.000	0.030	1043.994	100.000

Compound 25 MW:355.5

===

Injection Date : Tue, 8- Aug- 2017 19:59:21

Sample ID : EW7924-43-P1H8

Location : P1-B-07

Inj. Vol. : 0.5ul

Acq Method : D:\METHODS\CLG_A.M

Data Filename : D:\DATA\170808LCMS-F1\LCMS-F1H81BG1.D

Instrument : F

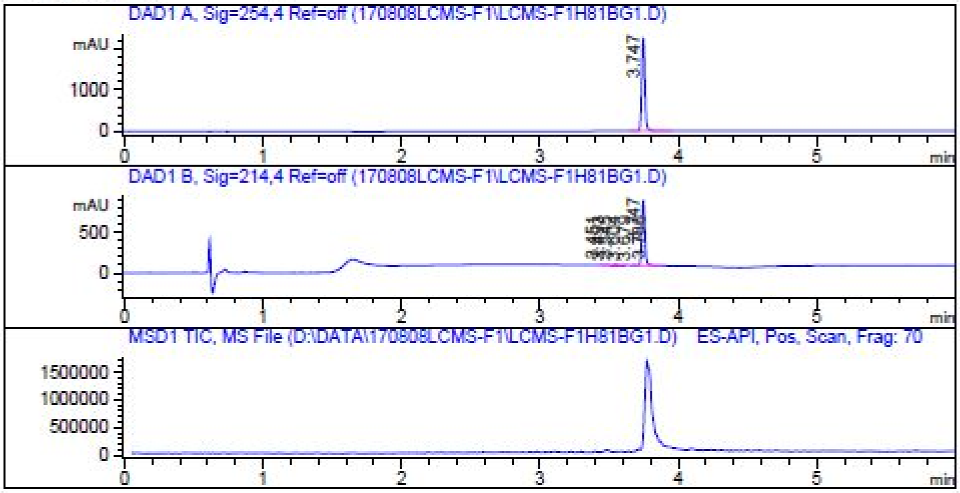

===

Report

===

Signal 1 : DAD1 A, Sig=254,4 Ref=off

Peak #	RT [min]	Height	Height %	Width [min]	Area	Area %
1	3.747	2244.346	100.000	0.024	3556.507	100.000

Signal 2 : DAD1 B, Sig=214,4 Ref=off

Peak #	RT [min]	Height	Height %	Width [min]	Area	Area %
1	3.451	3.268	0.398	0.022	4.355	0.345
2	3.493	1.927	0.235	0.016	1.820	0.144
3	3.545	3.727	0.454	0.041	9.238	0.732
4	3.604	0.998	0.121	0.010	0.788	0.062
5	3.679	2.491	0.303	0.019	3.645	0.289
6	3.747	794.477	96.705	0.026	1222.174	96.859
7	3.786	14.656	1.784	0.023	19.792	1.569

Compound 30 MW:342.4

```
=============================================
Injection Date     : Thu, 13- Jul- 2017              14:46:06
Sample ID          : EW8002-32-P1GD
Location           : P1-B-04
Inj. Vol.          : 1.0ul
Acq   Method       : D:\METHODS\CLG_A.M
Data Filename      : D:\DATA\170713LCMS-F1\LCMS-F1GD1BD1.D
Instrument         : F
```

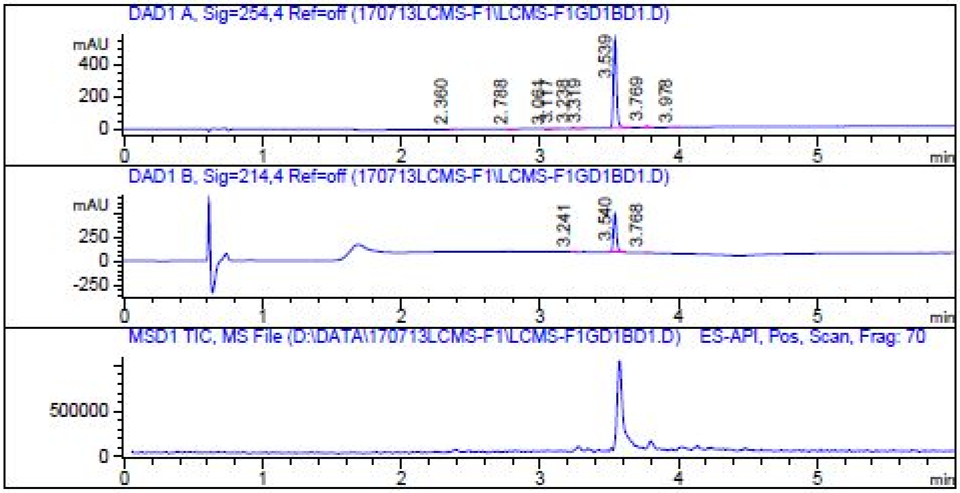

Report

Signal 1 : DAD1 A, Sig=254,4 Ref=off

Peak #	RT [min]	Height	Height %	Width [min]	Area	Area %
1	2.360	1.936	0.340	0.020	2.272	0.253
2	2.788	0.358	0.063	0.019	0.408	0.045
3	3.061	0.294	0.052	0.027	0.479	0.053
4	3.117	0.453	0.079	0.030	0.811	0.090
5	3.238	3.983	0.699	0.026	6.260	0.697
6	3.319	0.697	0.122	0.024	1.000	0.111
7	3.539	553.035	97.001	0.026	870.976	97.035
8	3.769	8.522	1.495	0.027	13.906	1.549
9	3.978	0.853	0.150	0.029	1.480	0.165

Signal 2 : DAD1 B, Sig=214,4 Ref=off

Peak #	RT [min]	Height	Height %	Width [min]	Area	Area %
1	3.241	3.121	0.763	0.023	4.346	0.678
2	3.540	402.676	98.384	0.026	630.991	98.513
3	3.768	3.492	0.853	0.025	5.180	0.809

Compound 31 MW:356.4

==

Injection Date	: Thu, 10- Nov- 2016	16:29:42
Acq Operator	: ADMIN	
Location	: P1-B-08	
Inj. Vol.	: 0.6 ul	
Acq Method	: D:\METHODS\CLG_A.M	
Data Filename	: D:\DATA\161110FG\FGKA1BH2.D	
Instrument	: LCMS-F	

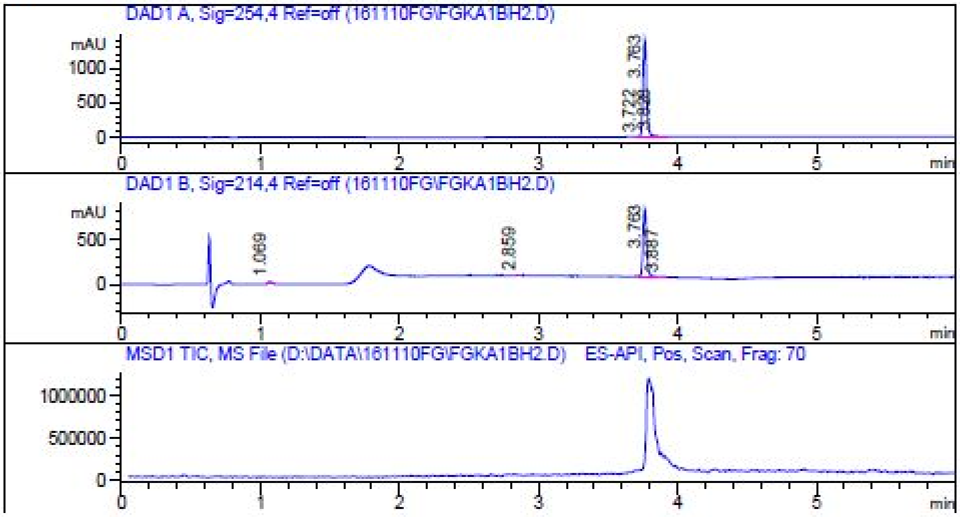

==

Report

==

Signal 1 : DAD1 A, Sig=254,4 Ref=off

Peak #	RT [min]	Area	Height	Height %	Width [min]	Area %
1	3.722	1.538	1.590	0.111	0.016	0.062
2	3.763	2463.062	1419.834	99.191	0.029	99.077
3	3.828	21.396	9.995	0.698	0.036	0.861

Signal 2 : DAD1 B, Sig=214,4 Ref=off

Peak #	RT [min]	Area	Height	Height %	Width [min]	Area %
1	1.069	51.684	26.683	3.411	0.032	3.779
2	2.859	2.954	1.960	0.251	0.025	0.216
3	3.763	1310.073	751.283	96.044	0.029	95.779
4	3.887	3.098	2.306	0.295	0.022	0.226

Compound 33 MW:424.4

```
==============================================
Injection Date    : Wed, 15- Nov- 2017              18:55:30
Sample ID         : EW8002-193-P1KF
Location          : P1-C-06
Inj. Vol.         : 1.0     ul
Acq   Method      : D:\METHODS\CLG_A.M
Data Filename     : D:\2017\201711\171115LCMS-F1\LCMS-F1KF1CF1.D
Instrument        : F
```

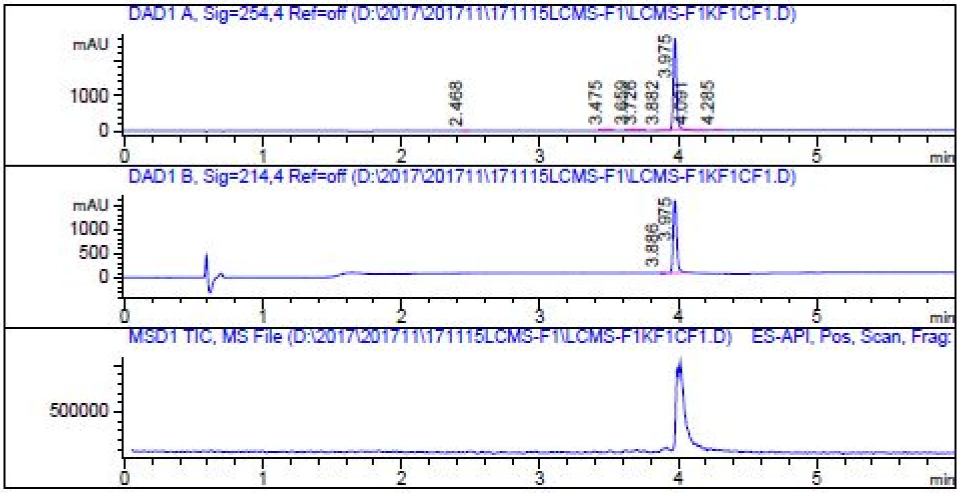

Report

Signal 1 : DAD1 A, Sig=254,4 Ref=off

Peak #	RT [min]	Height	Height %	Width [min]	Area	Area %
1	2.468	2.124	0.081	0.018	2.543	0.059
2	3.475	2.792	0.106	0.036	7.450	0.172
3	3.659	2.560	0.097	0.031	5.174	0.119
4	3.726	1.649	0.063	0.027	2.968	0.068
5	3.882	7.199	0.273	0.027	13.025	0.300
6	3.975	2603.759	98.899	0.027	4277.922	98.486
7	4.091	11.759	0.447	0.047	33.184	0.764
8	4.285	0.911	0.035	0.021	1.399	0.032

Signal 2 : DAD1 B, Sig=214,4 Ref=off

Peak #	RT [min]	Height	Height %	Width [min]	Area	Area %
1	3.886	14.138	0.931	0.016	13.570	0.499
2	3.975	1504.163	99.069	0.030	2704.240	99.501

Compound 34 MW:424.4

```
=================================================
Injection Date     : Fri, 8- Dec- 2017             4:53:46 PM
Sample ID          : EW8002-220-P1L8
Location           : P1-C-04
Inj. Vol.          : 2.0      ul
Acq   Method       : D:\METHODS\CLG_A.M
Data Filename      : D:\2017\201712\171208LCMS-F1\LCMS-F1L81CD1.D
Instrument         : F
```

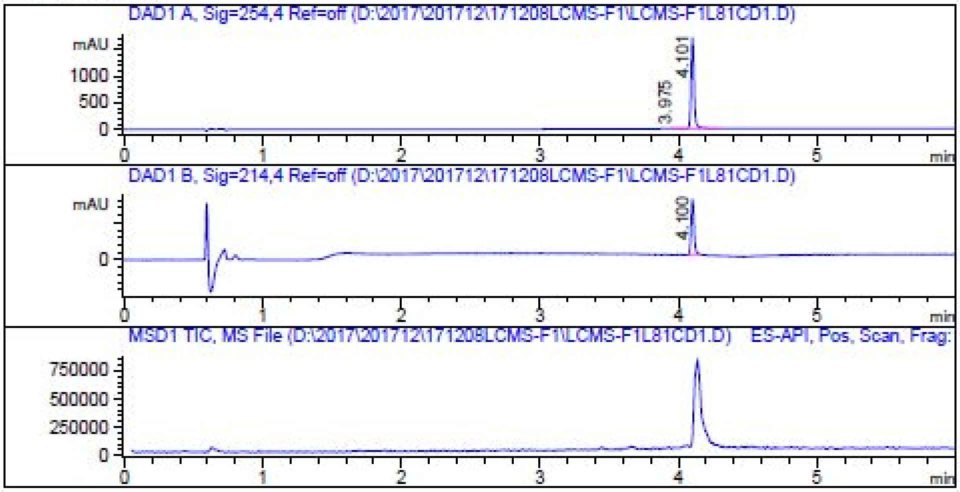

Report

Signal 1 : DAD1 A, Sig=254,4 Ref=off

Peak #	RT [min]	Height	Height %	Width [min]	Area	Area %
1	3.975	3.408	0.201	0.026	6.062	0.230
2	4.101	1689.026	99.799	0.024	2624.467	99.770

Signal 2 : DAD1 B, Sig=214,4 Ref=off

Peak #	RT [min]	Height	Height %	Width [min]	Area	Area %
1	4.100	735.491	100.000	0.025	1094.813	100.000

Compound 40 MW:328.4

```
==============================================
Injection Date    : Mon, 8- Apr- 2019              4:32:25 PM
Sample ID         : EW17777-5-P1D8
Location          : P1-D-01
Inj. Vol.         : 1.0     ul
Acq   Method      : D:\METHODS\CLG_A.M
Data Filename     : D:\2019\201904\190408LCMS-F1\LCMS-F1D81DA1.D
Instrument        : CAS-WH-LCMS-F
```

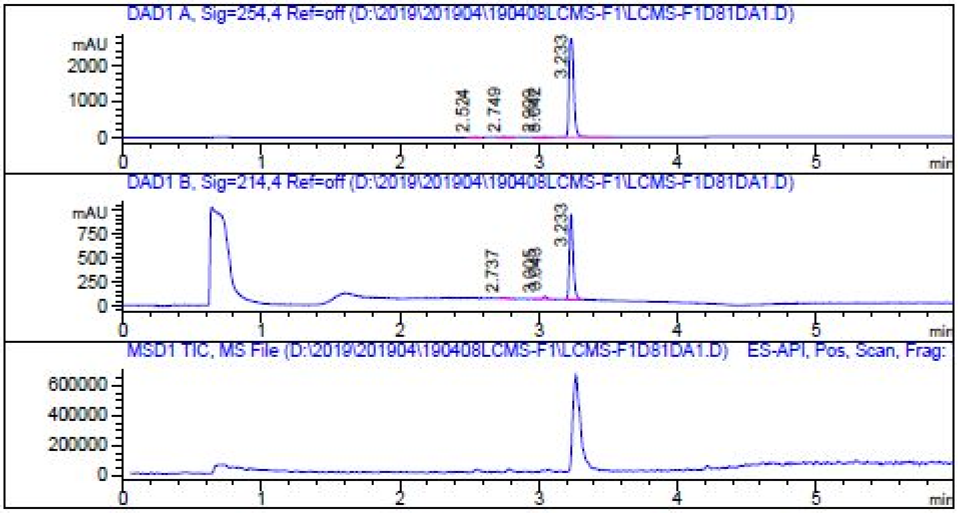

Report

Signal 1 : DAD1 A, Sig=254,4 Ref=off

Peak #	RT [min]	Height	Height %	Width [min]	Area	Area %
1	2.524	11.694	0.422	0.027	20.2426	0.308
2	2.749	17.666	0.638	0.027	31.2279	0.476
3	2.999	3.058	0.110	0.024	4.6647	0.071
4	3.042	17.707	0.639	0.026	30.9646	0.472
5	3.233	2720.719	98.191	0.038	6478.1641	98.673

Signal 2 : DAD1 B, Sig=214,4 Ref=off

Peak #	RT [min]	Height	Height %	Width [min]	Area	Area %
1	2.737	5.781	0.625	0.035	16.0584	0.901
2	3.005	6.322	0.684	0.017	8.2541	0.463
3	3.043	28.826	3.117	0.031	60.9807	3.420
4	3.233	883.901	95.574	0.029	1697.8527	95.217

Compound 43 MW:369.5

==
Injection Date : Mon, 18- Sep- 2017 18:12:34
Sample ID : EW8062-107-P1II
Location : P1-D-02
Inj. Vol. : 1.0ul
Acq Method : D:\METHODS\CLG_A.M
Data Filename : D:\2017\201709\170918LCMS-F1\LCMS-F1II1DB1.D
Instrument : F

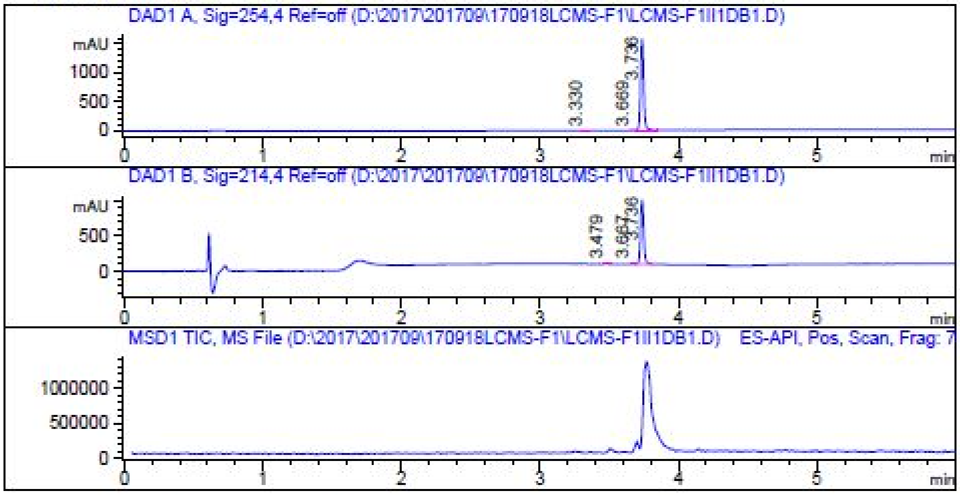

==
Report
==

Signal 1 : DAD1 A, Sig=254,4 Ref=off

Peak #	RT [min]	Height	Height %	Width [min]	Area	Area %
1	3.330	0.737	0.047	0.019	1.105	0.044
2	3.669	0.559	0.036	0.019	0.641	0.026
3	3.736	1568.791	99.917	0.025	2495.918	99.930

Signal 2 : DAD1 B, Sig=214,4 Ref=off

Peak #	RT [min]	Height	Height %	Width [min]	Area	Area %
1	3.479	6.046	0.665	0.021	7.583	0.516
2	3.667	3.152	0.347	0.011	2.106	0.143
3	3.736	900.434	98.989	0.027	1460.878	99.341

LCMS REPORT

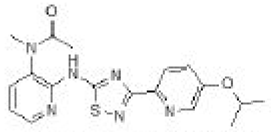

Chemical Formula: $C_{18}H_{20}N_6O_2S$
Exact Mass: 384.14

Compound ID : Compound 47
Sample ID : ET33765-33-P1A
Injection Date : 19. Mar. 2020
Inj. Vol. : 0.50 ul
Location : P2-D-07
Acq Method : D:\DATA\2003\200319 21\5_95AB_6min-220.M
Data Filename : D:\DATA\2003\200319 21\ET33765-33-P1A.D
Instrument : H

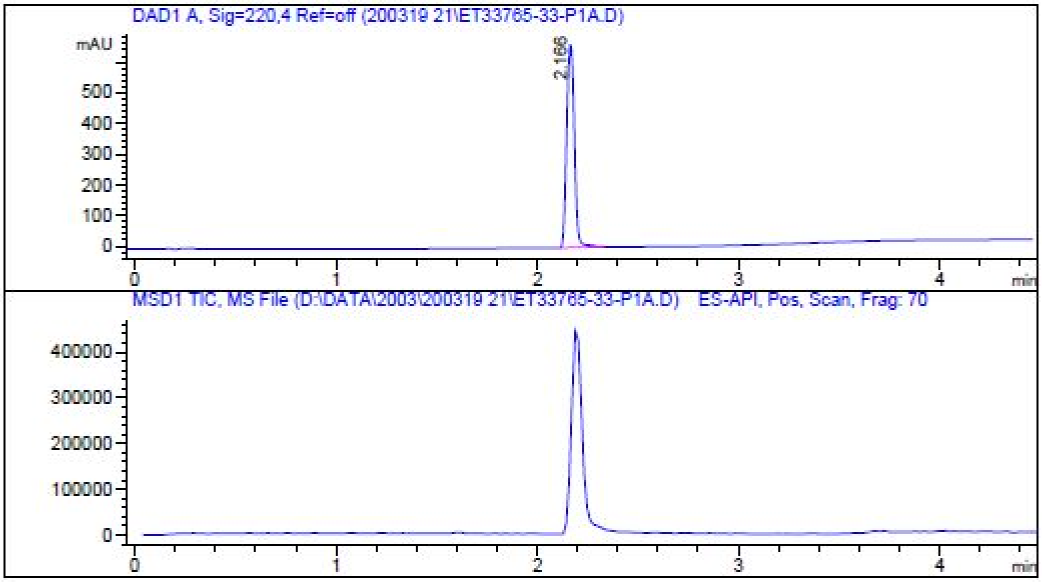

Integration Result

Signal 1 : DAD1 A, Sig=220,4 Ref=off

Peak #	RT [min]	Area	Height	Height %	Width [min]	Area %
1	2.166	1767.144	667.433	100.000	0.043	100.000

Compound 44 MW:369.5

```
===============================================
Injection Date    : Wed, 13- Sep- 2017              18:00:30
Sample ID         : EW7924-78-P1ID
Location          : P1-E-04
Inj. Vol.         : 1.0ul
Acq   Method      : D:\METHODS\CLG_A.M
Data Filename     : D:\2017\201709\170913LCMS-F1\LCMS-F1ID1ED1.D
Instrument        : F
```

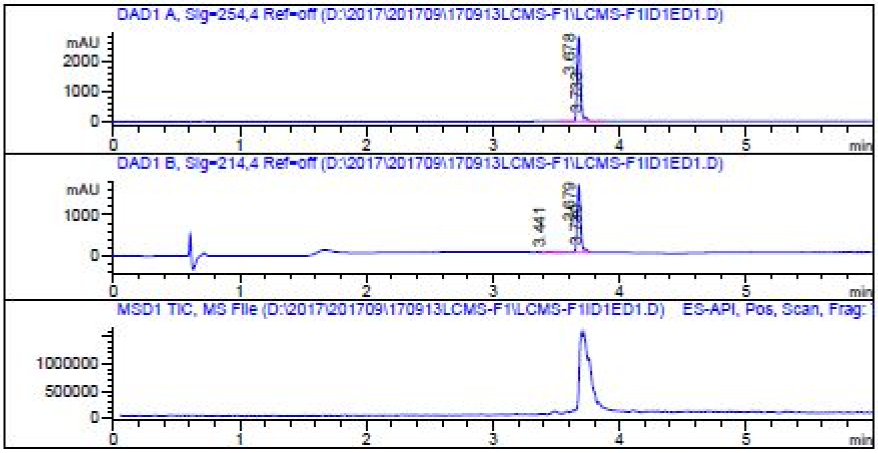


```
==========================================================================
                                  Report
==========================================================================
```

Signal 1 : DAD1 A, Sig=254,4 Ref=off

Peak #	RT [min]	Height	Height %	Width [min]	Area	Area %
1	3.678	2781.114	95.287	0.030	5177.177	95.647
2	3.733	137.545	4.713	0.025	235.604	4.353

Signal 2 : DAD1 B, Sig=214,4 Ref=off

Peak #	RT [min]	Height	Height %	Width [min]	Area	Area %
1	3.441	6.778	0.394	0.080	43.043	1.355
2	3.679	1634.375	94.990	0.031	3018.303	95.030
3	3.730	79.418	4.616	0.024	114.820	3.615

Compound 52 MW:464.5

==
Injection Date : Wed, 4- Apr- 2018 6:13:51 PM
Sample ID : EW11299-41-P1D4
Location : P2-B-05
Inj. Vol. : 1.0 ul
Acq Method : D:\METHODS\CLG_A.M
Data Filename : D:\2018\201804\180404LCMS-F1\LCMS-F1D42BE1.D
Instrument : LCMS-F

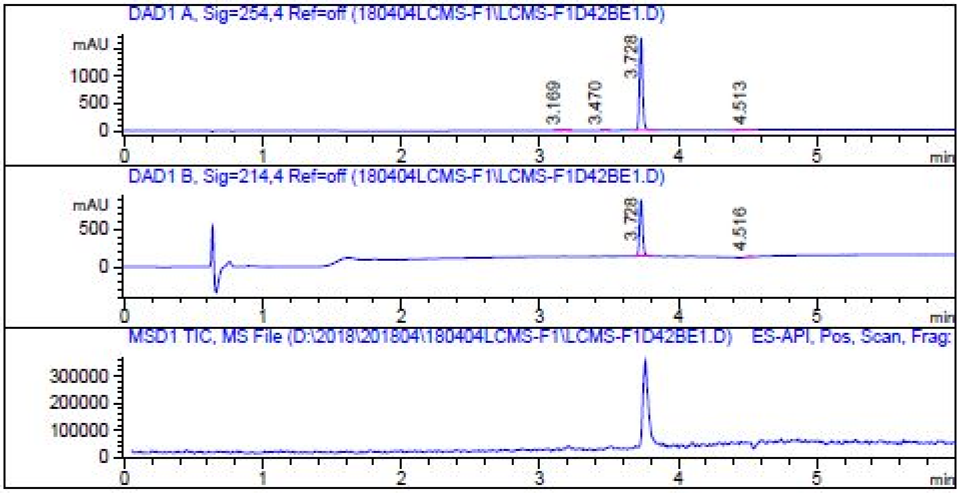

==

Report

==

Signal 1 : DAD1 A, Sig=254,4 Ref=off

Peak #	RT [min]	Height	Height %	Width [min]	Area	Area %
1	3.169	9.383	0.559	0.025	15.443	0.587
2	3.470	1.712	0.102	0.024	2.642	0.100
3	3.728	1660.179	98.940	0.024	2600.078	98.788
4	4.513	6.698	0.399	0.031	13.809	0.525

Signal 2 : DAD1 B, Sig=214,4 Ref=off

Peak #	RT [min]	Height	Height %	Width [min]	Area	Area %
1	3.728	744.112	98.439	0.024	1170.499	97.744
2	4.516	11.803	1.561	0.033	27.012	2.256

Compound 54 MW:452.5

==

Injection Date	: Fri, 26- Oct- 2018 3:11:18 PM
Sample ID	: EW11580-303-P1A2
Location	: P2-F-08
Inj. Vol.	: 2.0 ul
Acq Method	: D:\METHODS\CLG_A.M
Data Filename	: D:\2018\201810\181026LCMS-F1\LCMS-F1JQ2FH1.D
Instrument	: CAS-WH-LCMS-F

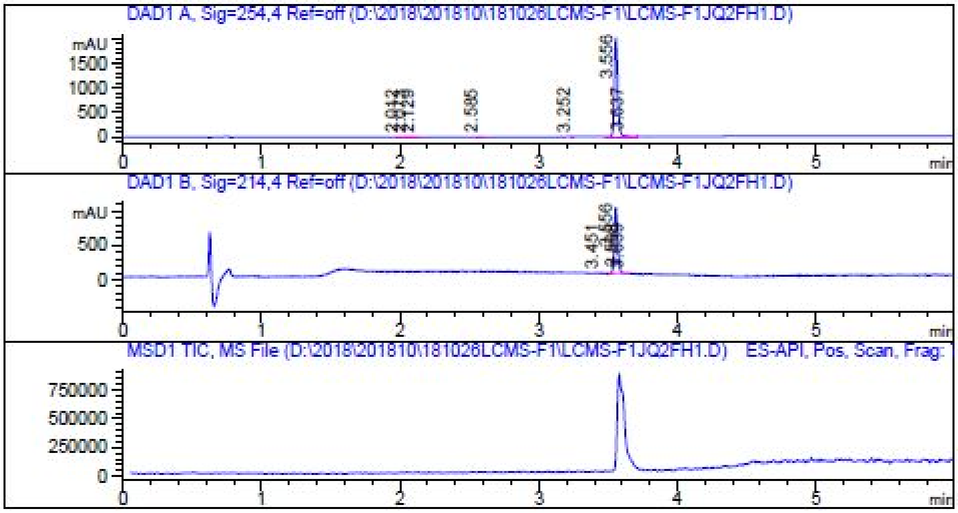

==

Report

==

Signal 1 : DAD1 A, Sig=254,4 Ref=off

Peak #	RT [min]	Height	Height %	Width [min]	Area	Area %
1	2.012	1.449	0.073	0.027	2.9662	0.085
2	2.073	0.644	0.032	0.006	0.2616	0.008
3	2.129	0.728	0.036	0.024	1.4043	0.040
4	2.585	0.714	0.036	0.019	0.9247	0.027
5	3.252	0.290	0.014	0.009	0.1978	0.006
6	3.556	1986.582	99.394	0.027	3454.4199	99.366
7	3.637	8.293	0.415	0.029	16.2896	0.469

Signal 2 : DAD1 B, Sig=214,4 Ref=off

Peak #	RT [min]	Height	Height %	Width [min]	Area	Area %
1	3.451	9.459	0.950	0.014	7.7527	0.465
2	3.556	964.841	96.854	0.028	1644.2423	98.724
3	3.598	14.660	1.472	0.007	6.0512	0.363
4	3.639	7.217	0.724	0.017	7.4498	0.447

```
Compound 57 MW:452.5

==============================================
Injection Date    : Wed, 5- Sep- 2018              11:23:07 PM
Sample ID         : EW11299-227-P1I5
Location          : P2-A-05
Inj. Vol.         : 4.0     ul
Acq   Method      : D:\METHODS\CLG_A.M
Data Filename     : D:\2018\201809\180905LCMS-F1\LCMS-F1I52AE1.D
Instrument        : CAS-WH-LCMS-F
```

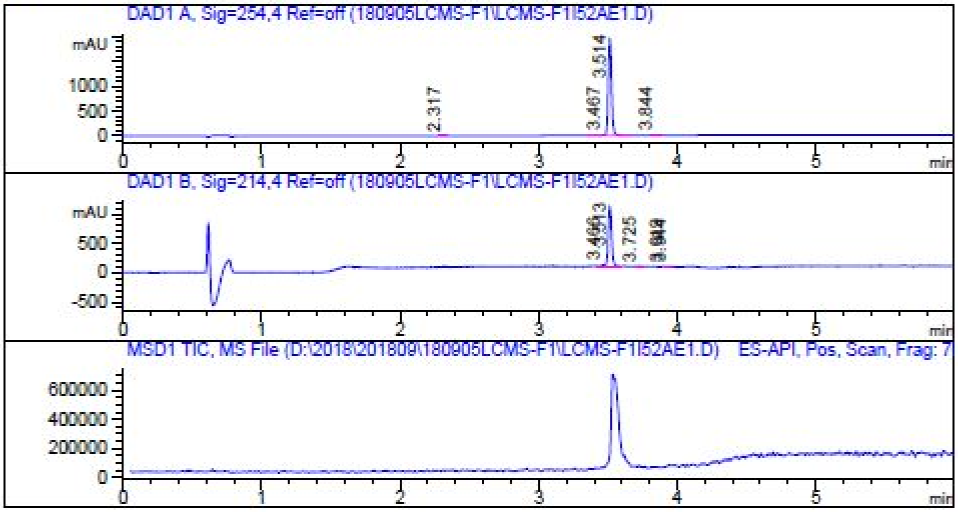


```
==========================================================================
                                  Report
==========================================================================
   Signal 1 : DAD1 A, Sig=254,4 Ref=off
   Peak    RT       Height     Height %    Width        Area      Area %
    #     [min]                            [min]
   ---------------------------------------------------------------------
     1     2.317       1.584      0.081      0.025       2.6000      0.080
     2     3.467      11.852      0.606      0.026      21.3814      0.654
     3     3.514    1939.727     99.240      0.026    3240.9424     99.191
     4     3.844       1.427      0.073      0.024       2.4633      0.075
   --- --------- ---------- --------- --------- ----------- ---------
   Signal 2 : DAD1 B, Sig=214,4 Ref=off
   Peak    RT       Height     Height %    Width        Area      Area %
    #     [min]                            [min]
   ---------------------------------------------------------------------
     1     3.466      26.894      2.511      0.023      41.2995      2.252
     2     3.513    1017.746     95.019      0.027    1763.8779     96.173
     3     3.725       9.220      0.861      0.016      10.8049      0.589
     4     3.919       8.359      0.780      0.014       7.9333      0.433
     5     3.944       8.880      0.829      0.015      10.1600      0.554
   --- --------- ---------- --------- --------- ----------- ---------
```

Reference

1. Bhuniya, D.; Mukkavilli, R.; Shivahare, R.; Launay, D.; Dere, R. T.; Deshpande, A.; Verma, A.; Vishwakarma, P.; Moger, M.; Pradhan, A.; Pati, H.; Gopinath, V. S.; Gupta, S.; Puri, S. K.; Martin, D., Aminothiazoles: Hit to lead development to identify antileishmanial agents. *European Journal of Medicinal Chemistry* **2015,** *102*, 582-593.
